# Effectiveness of Smartphone Application‐Based Interventions to Prevent Type 2 Diabetes Mellitus in Individuals With Prediabetes: A Systematic Review and Meta‐Analysis

**DOI:** 10.1111/obr.70028

**Published:** 2025-10-20

**Authors:** Laura Suhlrie, Nancy Abdelmalak, Jacob Burns, Hans Hauner, Niels Ole Kristiansen, Anna‐Janina Stephan, Michael Laxy

**Affiliations:** ^1^ Professorship of Public Health and Prevention TUM School of Medicine and Health, Technical University of Munich Munich Germany; ^2^ Institute for Nutritional Medicine TUM School of Medicine and Health, Technical University of Munich Munich Germany; ^3^ German Center for Diabetes Research (DZD) Neuherberg Germany

**Keywords:** app‐based intervention, meta‐analysis, prediabetes, systematic review

## Abstract

Smartphone application (app)‐based interventions to prevent Type 2 diabetes (T2D) are becoming increasingly available. A thorough summary of their effectiveness is lacking. We synthesized evidence on the effectiveness of app‐based interventions to prevent T2D targeting individuals with prediabetes. For this systematic review and meta‐analysis, we searched Web of Science, Embase, Scopus, PubMed Central, and Medline between January 1, 2013, and January 31, 2024, to identify randomized controlled trials (RCTs) that assessed the effectiveness of app‐based interventions to prevent T2D targeting individuals with prediabetes, published in English, without restrictions regarding the effectiveness outcome. We synthesized all outcomes graphically via effect directions and conducted meta‐analyses for clinical outcomes, including the Risk of Bias 2 Tool. This study was prospectively registered with PROSPERO (CRD42023491693) and OSF (DOI 10.17605/OSF.IO/B89QP). Of 9703 articles, 18 RCTs were included in the systematic review, and 15 RCTs in the meta‐analysis. We found statistically significant reductions in body weight (−1.35 kg, 95% CI: [−2.48; −0.23], *N* = 13 RCTs), body mass index (−0.53 kg/m^2^, 95% CI: [−0.97; −0.09], *N* = 11 RCTs), and glycated hemoglobin (−0.08%, 95% CI: [−0.10; −0.05], *N* = 11 RCTs) and point estimates and/or effect directions predominantly suggesting improvements for additional outcomes. Stratified meta‐analyses showed no statistically significant between‐group differences and missing evidence for long‐term effectiveness and equity‐relevant subgroups. Our study shows that app‐based interventions can improve outcomes (i.e., motivation, behavior, and clinical parameters) in individuals with prediabetes. The effect on clinical outcomes is small. Evidence on equity impacts, long‐term effectiveness, and diabetes incidence is missing and remains to be investigated.

## 1 Introduction

Diabetes has a high global prevalence of about 10% and is associated with an economic burden of USD 966 billion [1, 2]. Those numbers are expected to increase [1]. Type 2 diabetes (T2D) accounts for 90% of all diabetes cases worldwide. High‐quality RCTs and translation trials have shown that T2D can be prevented and treated with a healthy lifestyle, such as a healthy diet, regular physical activity (PA), and maintenance of healthy body weight [1, 3, 4, 5, 6, 7, 8]. Early identification of prediabetes, a condition of raised blood glucose levels preceding diabetes, creates opportunities for prevention interventions against T2D [1].

Behavioral change interventions for people with prediabetes are already partially implemented in the United States [9] and the United Kingdom [10], with about 1500 recognized programmes targeting 5%–7% weight loss and PA of 150 min/week [9, 11]. These programmes are also offered in digital formats (digital Diabetes Prevention Program, dDPP), which integrate smartphone apps to communicate with dietitians and peer groups and to track behaviors and goals [9, 10]. An observational study of dDPPs in 1817 participants in the United Kingdom observed a reduction in weight of 3.1 kg (95% confidence interval [CI]: −3.4, −2.8) after 12 months of follow‐up [12].

This illustrates that, just as for many other health challenges, digital health approaches are also gaining relevance for T2D prevention. However, to date, digital health comprises a wide range of definitions, including eHealth, mHealth, wearables, tracking devices, artificial intelligence (AI), and information systems in the healthcare sector, with no uniform consensus [13, 14]. Among the heterogeneous group of digital health therapies, especially smartphone apps have evolved rapidly with the introduction of reimbursement options for such therapies by health insurances [15]. Such reimbursement often requires proof of clinical effectiveness and comes along with a heightened interest in understanding the contribution of specific intervention characteristics such as face‐to‐face (f2f) components, which may drive costs and impede scalability, but potentially foster effect maintenance [16, 17]. Furthermore, specifically for smartphone app‐based interventions, which are heralded as potential solutions to increasing health inequalities [18], consideration of effect heterogeneity across specific health equality‐relevant characteristics such as ethnicity and education is needed to better understand the impact on health inequality. Given the pressing health burden of T2D and the potential of app‐based therapies, high‐quality evidence and synthesis of this evidence are needed to derive generalizable policy recommendations.

A previous systematic review and meta‐analysis, which assessed the effectiveness of app‐based interventions in populations with prediabetes, found significant weight and body mass index (BMI) reductions and no significant reductions in glycated hemoglobin (HbA_1c_) and waist circumference [19]. This meta‐analysis included only six RCTs, assessed only selected outcomes, and did not conduct stratified analysis nor examine effect heterogeneity [19]. In a meta‐analysis on eHealth effectiveness to prevent T2D, weight decreased by 3.98% (95% CI: [−4.49; −3.46]) over 15 months [20]. Other systematic reviews and meta‐analyses assessing the effectiveness of telemedicine interventions more broadly reported either statistically significant weight reductions [21] or inconclusive results [22, 23]. Given that previous reviews included either only a few studies and outcomes [19] or a wide range of digital, but partly already outdated intervention modes besides apps [20, 21, 22, 23], there is a need for a systematic assessment synthesizing available evidence on the effectiveness of app‐based interventions to prevent T2D in people with prediabetes. Such an assessment should also consider the complexity of these interventions by examining not only changes in health impacts but also in psychosocial and behavioral outcomes thought to be on the causal pathways from interventions to clinical outcomes, such as changes in motivation, self‐efficacy, health literacy, and lifestyle that translate to quality of life (QoL) and clinical changes.

In this systematic review and meta‐analysis, we address this research gap by (1) graphically synthesizing effect directions for all available effectiveness outcomes (i.e., motivation, self‐efficacy, health literacy, behavior, QoL, and clinical parameters) reported in RCTs, (2) conducting meta‐analyses on clinical outcomes, (3) conducting stratified meta‐analyses to explore effects related to study design characteristics (i.e., type of control, degree of behavioral support, follow‐up, inclusion criteria, and risk of bias), and (4) descriptively synthesizing subgroup analyses to explore effect heterogeneity related to equity (i.e., ethnicity, gender, education, and age).

## 2 Methods

This study follows Cochrane methodological guidance [24] and complies with the Preferred Reporting Items for Systematic Reviews and Meta‐Analyses (PRISMA) reporting statement (Appendices S1 and S2). We prospectively registered the protocol on PROSPERO (CRD42023491693) and the Open Science Framework (OSF) (DOI 10.17605/OSF.IO/B89QP). Several steps in the review, including the definition of the eligibility criteria, the search strategy, the data extraction, and the data synthesis, are based on a logic model that was developed ex ante by the authors. The logic model aims to help conceptualize and describe the complexity in how the effect of app‐based interventions to prevent T2D is established, as it moves along the causal pathway and is potentially influenced by individual and environmental factors (Appendix S3) [25].

### 2.1 Search Strategy and Selection Criteria

We searched Web of Science, Embase (via Ovid), Scopus, PubMed Central, and Medline (via PubMed) from January 1, 2013, to January 31, 2024. Search results were augmented through snowball searches within systematic reviews and meta‐analyses identified during title and abstract screening, as well as forward and backward citation searches of eligible RCTs. No substantial deviations were made from the protocol (Appendix S4). We aligned our search start date with Apple's App Store launch, which made apps broadly available [26, 27]. Inclusion criteria comprised (1) adult populations at high risk of developing T2D, defined as meeting at least one of the clinical prediabetes criteria—(i) fasting plasma glucose (FPG) of 100 mg/dL (5.6 mmol/L) to 125 mg/dL (6.9 mmol/L), (ii) 2‐h PG during 75‐g oral glucose tolerance test (OGTT) of 140 mg/dL (7.8 mmol/L) to 199 mg/dL (11.0 mmol/L), or (iii) HbA_1c_ 5.7%–6.4% (39–47 mmol/mol)—in accordance with the prediabetes definition from the American Diabetes Association (ADA) [28] or diabetes risk assessed via a score (i.e., FINDRISK); (2) the intervention being mainly app‐based and, for complex interventions with multiple components (e.g., diet assignment), that at least one of those components is delivered through an app with the explicitly or implicitly stated app purpose of preventing T2D; (3) randomized evaluation designs; and (4) peer‐reviewed reports with full text in English. Records were excluded if (1) results were not published, (2) they were conference abstracts, or (3) they included populations with diabetes yet did not report prediabetes results separately. We made no restrictions regarding outcomes (as long as they pertained to an aspect of effectiveness, i.e., motivation, self‐efficacy, health literacy, behavior, QoL, and clinical parameters) or control group. The search strategy was developed by two authors (A.J.S. and L.S.) and revised by an experienced librarian (Appendix S5).

After deduplication in EndNote [29], three authors (N.A., L.S., and N.O.K.) conducted the title and abstract screening independently and in duplicate using Rayyan [30]. Discrepancies were resolved in a consolidation meeting and, where necessary, clarified with a third investigator (A.J.S.). Full texts were screened in the same way as titles and abstracts. We calculated Cohen's kappa interrater reliability (IRR) coefficients.

### 2.2 Data Extraction

For each study, information was extracted independently and in duplicate by two out of three review authors (L.S., N.A., and N.O.K.) using a predefined Excel extraction sheet. We extracted key study information, including primary author, year of publication, study title, study design, country and region, intervention description, control group information, sample size, and population characteristics. Extractions for data synthesis included unit of measurement, type of effect measure, point estimate, 95% CI, standard error (SE), and *p*‐value. If available, coefficients from intention‐to‐treat analyses (ITT) with baseline outcome values included as a covariate in a regression model or analysis of covariance (ANCOVA) were extracted. Otherwise, we extracted unadjusted pre‐post intervention‐control difference‐in‐difference (DiD) estimates or post‐intervention differences. Where RCTs only reported mean changes within groups, we calculated the DiD estimates and pooled SE. Where RCTs reported only time‐ and group‐specific estimates, we contacted the authors and asked for DiD estimates. If we did not receive an answer, we calculated the pooled SE using a hypothetical correlation coefficient (*r*) of *r* = 0.9 (Appendix S6).

### 2.3 Risk of Bias (RoB) Analysis

Two authors (N.A. and L.S.) assessed separately each RCT using the Cochrane RoB 2 tool [31]. The tool rates bias across five domains: randomization process, deviations from the intended interventions, missing outcome data, measurement of the outcome, and selection of the reported result. Each domain is given one of three bias levels: low risk, some concerns, or high risk. The tool automatically, through an algorithm, calculates the overall risk of bias on the study level (i.e., low risk, some concerns, or high risk). Assessments from both reviewers were compared, and discrepancies were resolved in a consolidation meeting.

### 2.4 Data Analysis

We aimed to analyze the extracted effectiveness outcomes, in line with our logic model, through four consecutive steps, including (1) the graphical synthesis of effect directions for all effectiveness outcomes using a synthesis without meta‐analysis (SWiM); (2) the meta‐analysis of outcomes, where more than one RCT reported on the same outcome with sufficiently homogeneous operationalization; (3) stratified meta‐analyses to assess the influence of study characteristics; and (4) the descriptive assessment of health equity‐related effect heterogeneity. Each of these steps is described below:

#### 2.4.1 Graphical Synthesis of all Effectiveness Outcomes

We clustered the outcomes and their effect directions according to the structure of the logic model to represent the different aspects of effectiveness on lifestyle and behavioral outcomes. We synthesized all effectiveness outcomes (nonclinical and clinical outcomes) via effect directions for the longest follow‐up. In addition, we organized effect directions by follow‐up subgroups (1 to ≤ 3 months, > 3 to ≤ 6 months, and > 6 months). For the synthesis of effect directions, we drew on the SWiM in systematic reviews' guidelines, where the magnitude of the effect estimates is considered quantitatively and qualitatively [32].

#### 2.4.2 Meta‐Analysis of Clinical Outcomes

We conducted meta‐analyses for all outcomes for which at least two RCTs reported estimates. We used random‐effects models as we assumed considerable between‐study heterogeneity caused by likely context‐specific effects, differences between apps, and the pragmatic nature of such RCTs, limiting standardization. To estimate between‐study heterogeneity, we calculated tau‐squared (*τ* ^2^) with the DerSimonian–Laird procedure and Higgins & Thompson's *I* ^2^ statistic [33, 34]. We used Knapp–Hartung adjustments to control for the uncertainty of the between‐study heterogeneity [35]. As suggested by the Cochrane handbook, we meta‐analyzed the longest follow‐up time point. Also, in line with the handbook, in RCTs with two intervention groups, we aimed to ensure that the group that we included in the meta‐analysis was the one that was most closely aligned with our review scope and that the comparison remained conceptually and statistically appropriate. Specifically, in RCTs with two app‐based intervention groups, we either included the intervention group that came closest to providing participants with an app disentangled from any other intervention components or, if both intervention arms contained additional features along with the app component, we combined the intervention group effects by calculating the combined mean and SE [36]. If RCTs had two intervention groups but one without an app‐based intervention, we excluded the latter. By only including one comparison in the case of three study arms, we followed the Cochrane recommendation to avoid the unit‐of‐analysis problem, meaning that the same group of participants is included twice in the same meta‐analysis [36]. To assess robustness, we conducted influence analyses using the leave‐one‐out method, which aims to assess whether one particular study disproportionately drives the observed result [37]. We did this by re‐estimating each meta‐analysis *n* number of times, with *n* representing the number of studies contributing data to the respective meta‐analysis. In each re‐estimation, one study was left out of the sample. We then created two forest plots, one sorted by the pooled effect size and the other by the *I*² value of the leave‐one‐out meta‐analyses to allow for an assessment of each study's impact on the results of the overall meta‐analysis. In these plots, each row is labeled by the study that is left out of the respective analysis.

All analyses were conducted in RStudio (version 4.3.1) with the “meta,” “metafor,” and “metadata” packages [38].

#### 2.4.3 Stratified Meta‐Analysis to Assess the Influence of Study Characteristics

We investigated potential sources of methodological and clinical heterogeneity for outcomes for which *N* > 5 RCTs were included. Specifically, we conducted stratified meta‐analyses for the degree of behavioral support, type of control group, type of inclusion criteria, and follow‐up duration. To assess effect maintenance, we plotted effect estimates over time for each RCT that reported more than one follow‐up and conducted a post hoc subgroup analysis (< 12 vs. 12 months) [39, 40]. We conducted sensitivity analyses for each outcome stratified by the RoB subgroup of the RCT in which the outcome was measured. For this purpose, outcomes were categorized as derived from either “low,” “some,” or “high” RoB studies.

#### 2.4.4 Descriptive Assessment of Health Equity‐Related Effect Heterogeneity

We synthesized subgroup analyses descriptively, through reporting and comparing effect directions (instead of the effect magnitudes that are used in quantitative syntheses), to explore effect heterogeneity related to equity following PROGRESS‐Plus, a Cochrane framework to identify population characteristics for stratification [41].

### 2.5 Reporting Bias and Sensitivity Analyses

We explored publication bias via funnel plots and the Egger's test [39, 40] for meta‐analyses with at least 10 RCTs, as uninformative patterns are likely to be observed for fewer RCTs. In additional sensitivity analyses, we assessed the impact of the additional assumption that we had to make when studies provided raw means at baseline and follow‐up, but no mean differences for an outcome. In these cases, we carried out sensitivity analyses by replacing the assumption of autocorrelation in the respective outcome measurements between baseline and follow‐up of *r* = 0.9 with *r* = 0.5, 0.7, and 0.95 to assess the impact of this assumption on the results.

## 3 Results

### 3.1 Search Results

**FIGURE 1:**
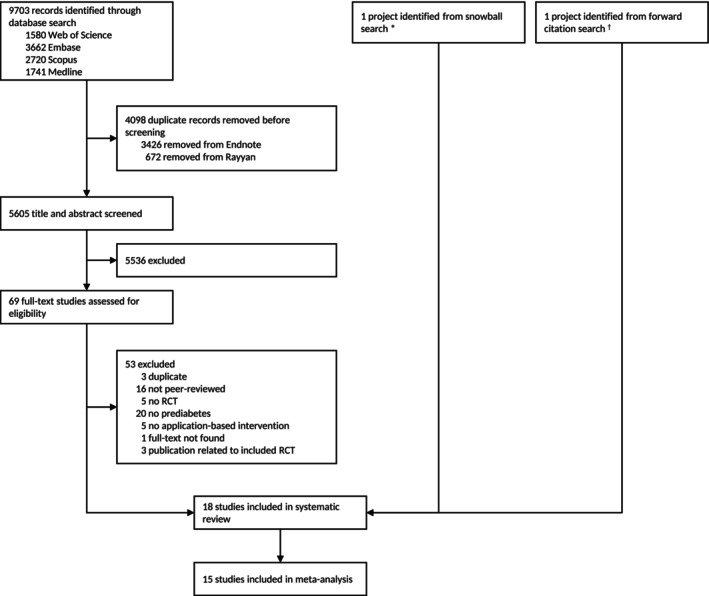
PRISMA flowchart of study search and selection strategy. *This article was not identified in the original search because it included broader terms for mobile health that we did not cover in our search strategy.†This article was not identified in the original search because it was published after the end of our search.

A total of 9703 articles were retrieved, of which 5605 remained after deduplication for title and abstract screening. Sixty‐nine articles were assessed through full‐text screening. Of these, we excluded 53 and identified two additional articles through snowball [42] and forward citation searches [43]. In total, 18 RCTs were included (Figure 1). In the Supporting Information, we provide reasons for study exclusions at full‐text screening (Appendix S7) and Cohen's kappa IRR coefficients (Appendix S8).

### 3.2 Study Characteristics

Most RCTs were conducted in the United States (*N* = 6), followed by China and the United Kingdom (*N* = 3 each). The 18 RCTs involved 6156 participants in total. The mean age was 54 years (SD = 10.0). One RCT only reported the minimum age of participation (18+ years) [44]. All RCTs included participants of both sexes, of which 64.0% (*N* = 3942) were females. Eleven RCTs provided information on the education level of study participants and showed greater representation of high education. Most RCTs had either usual care (UC) (*N* = 6) or app/device‐based (*N* = 6) comparators and no f2f support in the intervention group (*N* = 12). Nine RCTs required high BMI as an additional inclusion criterion. Intervention durations ranged from 1.5 to 13 months (mean = 7.1, SD = 3.9). The latest follow‐up measurements ranged from 1.5 to 12 months (mean = 7.1, SD = 3.8) (Table 1).

**TABLE 1:** Study characteristics.

	Study design	Country, region/city	Population	Participant characteristics		Intervention characteristics					
Authors and year of publication			Total population (N) *N* Intervention *N* Control	Sex, females, %	Age range (mean age), years	Prediabetes definition (& BMI)	Intervention description	Behavioral support in intervention group	Control description (category^f^)	Intervention duration, months	Follow‐up measurements, months
Bender et al. (2018)^a^	Pilot RCT (active wait‐list control)	USA San Francisco, California	67 33 34	52.2%	18+ (41.7)	Diabetes risk test score > 5 points, FPG 100–125 mg/dL, HbA1c > 5.6%, or OGTT 140–200 mg/dL BMI for Asians > 23 kg/m^2^	Culturally adapted weight loss lifestyle programme with mobile app/diary and Facebook group, 5 in‐person office visits (f2f) for goal setting, logging steps with Fitbit Zip min. of 10 h/day, recording food/drink intake daily, and weight weekly. Discussion topics daily on Facebook group	f2f support	Fitbit zip pedometer/accelerometer, educational materials on hepatitis A/B (1)	6 (3 intensive phase + 3 maintenance)	3, 6
Ben‐Yacov et al. (2021)^a^	Biphasic RCT, single‐blind	Israel Rehovot	225 113 112	59%	18–65 (50)	FPG 100–125 mg/dL (5.6–6.9 mmol/L) and HbA1c 5.7%–6.5% (39–48 mmol/mol)	Algorithm‐based personalized diet with real‐time feedback via app to lower postprandial glucose responses (PPGRs), eight 30‐min on‐site (f2f) dietitian consultations, phone/email support anytime, questionnaires promoted retention, CGM in months 1–6, 9, and 12. Daily dietary intake logged in real time through app	f2f support	MED diet, onsite dietitian sessions (30 min in Months 1–6, 9, and 12), contact via phone/email anytime, retention‐promoting questionnaires (Months 1–6 and 12), CGM use (Months 1–6 and 2 weeks in Months 9 and 12, blinded), daily dietary intake logging via app (3)	12 (6 intervention phase + 6 FU phase)	6, 12
Block et al. (2015)^a^	RCT (wait‐list control)	USA California, Berkeley	339 163 176	31.3%	30–69 (55)	Fasting glucose 5.55–6.94 mmol/L (100–125 mg/dL) or HbA1c 39–46 mmol/mol (5.7%–6.4%) BMI ≥ 27 kg/m^2^ (for Asian participants > 25 kg/m^2^)	Midweekly automated email and mobile reminders, weekly goal setting via web, email, voice response, phone calls, and the app, weight/diet/PA tracking, weekly health information on diabetes prevention plus quizzes. Social support via virtual teams and messaging, additional funding: extending intervention to 12 months, no f2f	No f2f support	Usual care (1)	6	3, 6
Chen et al. (2020)^a^	Pilot RCT, single‐blind	China Tianjin	138 69 69	61%	30–80 (53.6)	FPG 100–125 mg/dL (5.6–6.9 mmol/L), HbA1c 5.7%–6.4% and glucose postprandial blood 140 mg/dL (7.8 mmol/L) 1–2 h after ingestion of food	Routine health education by dietitian, initially daily, then weekly low‐carb dietary guidance by dietician, support via app and f2f session, meal/sleep/PA tracking, 2‐weeks CGM use (masked measurements)	f2f support	Routine health education by dietician for adults with prediabetes, 2 weeks app tracking, 2‐week CGM use (masked), intervention available post‐study (3)	3	3
Chung et al. (2023)^a^ ^,^ ^b^	Open‐label, parallel‐group RCT (3 groups)	Taiwan Northern Taiwan	121 41 (Ordinary mHealth Group) 42 (Traditional Chinese Medicine Group) 38	53%	20+ (58.1)	HbA1c 5.7%–6.4% or FPG 100–125 mg/dL	Ordinary mHealth Group 1: health education by GP (f2f) + app instructions, milestones in app, personal and group chats with feedback and encouragement, weight/BMI/blood sugar/diet/PA tracking. Info on prediabetes, DASH diet, PA, weekly text reminders, quizzes Traditional Chinese Medicine Group 2: all from group 1 plus education on body constitution and meridian energy, diet, and PA advice based on body constitution, Qigong videos, pictures, illustrations	f2f support	Usual care (15–20 min health education by GP) (1)	3	3, 4
Fukuoka et al. (2015)^a^	Feasibility RCT	USA California: San Francisco and Berkeley	61 30 31	77%	35+ (55.2)	Diabetes risk test score ≥ 5 points, FPG 100–125 mg/dL, HbA1c 5.7%–7.0%, or OGTT 140–200 mg/dL BMI ≥ 25 (for Asian participants BMI ≥ 23 kg/m^2^)	Pedometer, 6 in‐person sessions (f2f, month 0.5–4), daily logging of weight, activity, and calorie intake, daily reminders, interactive content (messages, videos, quizzes), and short‐ and long‐term goal setting	f2f support	Pedometer and brochure about prediabetes (3)	5	3, 5
Griauzde et al. (2019)	Pilot RCT (3 groups)	USA Ann Arbor, Michigan	69 24 (Group 1) 22 (Group 2) 23	64%	Not stated (51.7)	HbA1c 5.7%–6.4%	Group 1 (App‐only): Daily logging of sleep, presence, PA, creativity, and eating (S.P.A.c.e.), aligned with personal core values; optional daily reminders; and an information handout on prediabetes prevention strategies Group 2 (App‐plus): Same as Group 1, plus physical activity tracker and digital scale for self‐monitoring, with Fitbit data used for tailored messages and health tips No f2f	No f2f support	Information handout about prediabetes and T2D prevention, mHealth tools list for lifestyle monitoring (2)	3	3
Karvela et al. (2024)^a^ ^,^ ^c^ ^,^ ^g^	Open‐label pilot RCT	United Kingdom London	147 50 (Group 1) 46 (Group 2) 51	69%	Not stated (59)	HbA1c 6.0%–6.4%, IFG and IGT within non‐diabetic hyperglycaemic range	Group 1 (Intervention arm) * DNA‐personalized dietary advice via genetic report Group 2 (Exploratory arm): Same as Group 1, plus a self‐guided app and wearable device for scanning food barcodes. Genetic information was used to provide customized food and drink recommendations, suggesting healthier alternatives when shopping No f2f	No f2f support	Usual care (general healthy eating dietary advice according to NICE guidelines) (1)	6.5	1.5, 3, 6.5
Katula et al. (2022)^a^	RCT (wait‐list control)	USA Nebraska, Omaha	599 299 300	61%	19+ (55.4)	HbA1c 5.7%–6.4% (39–46 mmol/mol) BMI ≥ 25 kg/m^2^ (for Asian participants ≥ 22 kg/m^2^)	Using internet‐enabled devices (laptop, tablet, or smartphone) to asynchronously complete weekly behavior change lessons, communicate privately with lifestyle health coach, engage in peer group discussions, track meals, monitor weight, and track PA via wearable devices (own or request pedometer). Health coaches facilitated group discussions (about 2 posts/week) and provided feedback and advice through secure messaging (1–3 messages/week), reinforcing lesson content No f2f	No f2f support	Enhanced standard care (1 session diabetes prevention education) and wait‐list control (2)	12 (6 intensive + 6 maintenance)	4, 12
Kitazawa et al. (2023)^a^	Prospective unblinded RCT	Japan N/A	168 82 86	40%	20–80 (48.1)	HbA1c 5.6%–6.4% or fasting blood glucose 110–125 mg/dL BMI > 23 kg/m^2^ and < 40 kg/m^2^	Tracking of food and physical activity records (manually or via fitness devices) as well as diabetes‐related information (e.g., medications, BW, and blood pressure). Personalized messages based on preset lifestyle intervention, offering diet and exercise advice to prevent T2D, with input from CGM. CGM used for 6 weeks (Weeks 0–2, 4–6, and 8–10) and connected to app: display glucose fluctuations in sync with meals and PA No f2f	No f2f support	No lifestyle modification information and request not to use health care apps (1)	3	3
Lakka et al. (2023)^a^ ^,^ ^d^	Multicenter, unblinded, pragmatic RCT (3 groups)	Finland North Savo, South Karelia, Päljät‐Häme	2907 967 (Group 1) 971 (Group 2) 969	80%	18–74 (55.1)	12+ points in FINDRISC or previous gestational diabetes	Group 1 (Digi): Habit library with 489 behavioral suggestions in 13 lifestyle categories, goal setting, daily self‐monitoring, and summary feedback for habit formation Group 2 (Digi+): Same as Group 1, plus 6 in‐person group coaching sessions (f2f) over first 6 months	f2f support	Digital information on T2D lifestyle risk factors and on PA recommendations (2)	12	12
Lim et al. (2022)^a^	Parallel multicentre RCT	Singapore	148 72 76	40%	21–75 (53.1)	IFG 6.1–6.9 mmol/L or IGT with 2‐h plasma glucose of 7.8–11.0 mmol/L after a 75‐g OGTT BMI ≥ 23 kg/m^2^	Monitoring through app: weight, diet, PA, blood glucose levels, behavioral strategies such as goal‐setting and motivational interviewing, automated system gives instant feedback on food choices, step counts had adaptive targets, weekly educational videos for 12 weeks, individualized health coaching, virtual interactions via app chat, glucometer for blood glucose monitoring No f2f	No f2f support	Usual care (standard dietary counseling and encouragement for 150 min/week PA), digital scale (3)	6	3, 6
Luo et al. (2022)^a^ ^,^ ^e^	Parallel‐arm RCT	China Shanghai	253 84 (Group 1) 85 (Group 2) 84	15%	25–60 (37.7)	Fasting glucose ≥ 5.6 mmol/L BMI ≥ 24 kg/m^2^	Group 1 (MED): weekly feeding regimen of 3 meals/day, electronic scale, smart band, and app to monitor weight, steps, satiety, and leftover photos. Meals: MED style Group 2 (TJD): weekly feeding regimen of 3 meals/day, similar monitoring tools. Meals: traditional southeast Chinese cuisine No f2f	No f2f support	App, control diet: according to China Health and Nutrition Survey (3)	6	6
McLeod et al. (2020)^a^ ^,^ ^h^	Parallel‐arm single‐blind RCT	New Zealand Wellington and Waikato	225 110 (prediabetes analysis) 115 (prediabetes analysis)	51%	18–75 (62.1)	HbA1c 41–49 mmol/mol (5.9%–6.6%)	Core phase: individual health coaching, provision of evidence‐based resources; core phase and maintenance: online peer support (forum) and online goal tracking No f2f	No f2f support	Usual care (1)	12 (4 core + 9 maintenance)	4, 12
Staite et al. (2020)^a^	Parallel‐arm single‐blind RCT	UK London	200 98 102	53%	18–65 (52.3)	HbA1c 39–47 mmol/mol (5.7%–6.5%)	Wristband from Buddi Ltd. with instructions for operation and downloading the study‐specific app, 22 web‐based education sessions on diet, PA and mental resilience, PDF transcripts and SMS notifications for new modules. Motivational SMS texts via app using MI principles, supporting healthy intentions and self‐monitoring, with 3–4 messages/day (excluding weekends) for 12 months No f2f	No f2f support	Buddi wristband for study duration, access to activity data, and web‐based education via app. 22 automated session reminders: weekly (Modules 1–6), biweekly (Modules 7–16), monthly (Modules 17–22), no additional messages sent (3)	12	6, 12
Toro‐Ramos et al. (2020)^a^	Parallel‐arm RCT	USA Long Island, New York	202 103 99	71%	18+ (56.6)	HbA1c 5.7%–6.4% (within 3 months before study enrolment)	Individual coach communication and monitoring, group messaging and daily behavior change challenges, DPP‐based education articles weekly for 20 weeks (up to 52 weeks), logging food (color coding), steps, exercise, and weight (weekly), automated feedback on food choices, weekly goal‐setting No f2f	No f2f support	Paper‐based DPP curriculum (2)	13 (5 core + 6 maintenance)	6, 12
Whelan et al. (2019)	Feasibility RCT (3 groups)	UK Leicestershire	45 Group 1: 15 Group 2: 15 Group 3: 15	60%	40+ (56)	Leicester risk assessment score 16–47 and HbA1c < 6.5	Group 1: glucose feedback from the FreeStyle Libre for first 4 weeks, then PA feedback from Fitbit for 2 weeks Group 2: Fitbit feedback for first 4 weeks, then also feedback from FreeStyle Libre for 2 weeks Group 3: feedback from FreeStyle Libre and Fitbit in parallel for 6 weeks No f2f	No f2f support	N/A	1.5	1.5
Xu et al. (2020)	Parallel‐arm RCT	China Beijing	81 40 41	45%	18+ (not stated)	ADA screening tool score of 5 or more	Push notifications twice/week and educational content to improve eating habits and PA, educational material sent by WeChat subscription account (DHealthBar), WeChat applets with online questionnaires, and a check‐in applet serving as an online forum. The check‐in forum to record and share daily diets and exercises via photos and texts, options to appreciate and comment on each other's posts to encourage peer support No f2f	No f2f support	Behavior change handbook on diet and PA (2)	6	3, 6

Abbreviations: ADA—American Diabetes Association; BMI—body mass index; BW—body weight; CGM—continuous glucose monitoring; DASH—Dietary Approaches to Stop Hypertension; DNA—deoxyribonucleic acid; DPP—Diabetes Prevention Programme; f2f—in‐person face‐to‐face; FPG—fasting plasma glucose; FU—follow‐up; GP—general practitioner; HbA1c—glycated hemoglobin; IFG—impaired fasting glucose; IGT—impaired glucose tolerance; MED—Mediterranean diet; OGTT—oral glucose tolerance test; PA—physical activity; PPGR—postprandial glucose response; RCT—randomized controlled trial; S.P.A.C.E.—(1) Sleep; (2) Presence; (3) Activity; (4) Creativity; and (5) Eating; T2D—Type 2 diabetes mellitus; TJD—traditional Jiangnan diet high in plants; UC—usual care.

^a^ Included in meta‐analysis.

^b^ In the meta‐analysis, we included the comparison between Group 1 (Ordinary mHealth) and the control.

^c^ In the meta‐analysis, we included the comparison between Group 2 (Exploratory arm) and control.

^d^ In the meta‐analysis, we included the comparison between Group 1 (Digi) and the control.

^e^ In the meta‐analysis, we combined both intervention groups.

^f^ Category specification of control condition: (1) UC; (2) Enhanced UC (incl. information material, paper‐based curriculum, and f2f support); and (3) App and/or device.

^g^ Only Group 2 was considered in the meta‐analysis.

^h^ Only the prediabetes range was considered in the meta‐analysis.

### 3.3 RoB Analysis

Our RoB assessments resulted in *N* = 1 RCT [45] having high RoB, *N* = 12 RCTs [43, 46, 47, 48, 49, 50, 51, 52, 53, 54, 55, 56] having some concerns, and *N* = 4 RCTs [42, 57, 58, 59] having low RoB (Appendix S9).

### 3.4 Data Analysis

As described above, our data analysis comprised four iterative steps, with the findings of each described in detail below.

#### 3.4.1 Graphical Synthesis of all Effectiveness Outcomes

We included 12 RCTs assessing *N* = 18 psychosocial and behavioral outcomes, two RCTs assessing *N* = 6 QoL outcomes (Table 2 and Appendix S10), and 16 RCTs assessing *N* = 54 clinical outcomes (Appendix S11), and we synthesized these outcomes graphically through summarizing effect directions. Three of four RCTs suggested that app‐based interventions increased motivation [45], self‐efficacy [48], and health literacy [45].

**TABLE 2:** Effect directions of psychosocial, behavioral, and quality of life outcomes for the longest follow‐up.

	Psychosocial and behavioral outcomes											Outcome					
First author, year	Motivation			Self‐efficacy	Health literacy	Lifestyle (nutrition and physical activity behavior)						Quality of life					
	Motivation (autonomous motivation to prevent T2DM)	Stage of dietary behavior change	^1^Stage of physical activity change/^2^activity volition	Self‐efficacy (^1^self‐efficacy for physical activity, ^2^barriers to being active, ^3^social support for physical activity)	Knowledge of health behavior/ability to select health information	Physical activity (^1^self‐reported/^2^not self‐reported) /^3^decrease in sedentary time	Healthier diet	Dietary/eating behavior	Energy/caloric intake	^1^Sweets, ^2^alcoholic drinks, ^3^SSB, & ^4^sugar intake	Salt intake	Stress	Fatigue/sleep quality	Comprehensive assessment	Physical component	Mental component	Meridian body energy
Ben‐Yacov, 2021	..	..	..	..	..	▲^1^	..	..	▲	..	..	..	..	..	..	..	..
Chen, 2020	..	..	▲^2^	..	▲	▲^b, 1^	▲▲^b^	▲^b^	..	▲^1, 2^	..	▼	▲	▲^b^	..	..	..
Chung, 2023	..	..	..	..	..	▲^1^	..	▼	..	..	..	..	..	..	▲	▲	▼
Fukuoka, 2015^d^	..	..	..	▲^1^▲^a, b, 2^▲^3^	..	▲^a, b, 1^▲^a, b, 2^	..	..	▲	▲^a, b, 3^	..	..	..	..	..	..	..
Griauzde, 2019	▲	..	..	..	..	..	..	..	..	..	..	..	..	..	..	..	..
Karvela, 2024	..	..	..	..	..	..	..	..	●	..	▼	..	..	..	..	..	..
Kitazawa, 2023	..	..	..	..	..	..	..	..	▲	..	..	..	..	..	..	..	..
Lakka, 2023	..	..	..	..	..	▲^1^▲^3^	▲	..	..	..	..	..	..	..	..	..	..
Lim, 2022	..	..	..	..	..	▲^1^	..	..	▲^b^	▲^b, 4^	..	..	..	..	..	..	..
Staite, 2020	..	..	..	..	..	▼^c, 1^▼^2^▼^c, 3^	..	..	..	..	..	..	..	..	..	..	..
Whelan, 2019	..	..	..	..	..	N/A^c, 2^	..	..	..	..	..	..	..	..	..	..	e
Xu, 2020^e^	..	▲^a^	▲^a, 1^	..	..	▲^1^	..	..	▲^a^	..	..	..	..	..	..	..	..

*Note:* The arrows show the effect direction toward improvement or worsening of the outcome in the intervention group in comparison with the control group for the longest follow‐up assessment. More than one arrow can be under one outcome in a study if the study used several instruments to measure this outcome. A detailed overview of estimates can be found in Appendix S10.

Abbreviations: IPAQ: International Physical Activity Questionnaire; PAR: physical activity recall; SSB: sugar‐sweetened beverage; T2DM: Type 2 diabetes mellitus.

▲Effect direction toward better outcome in the intervention than in the control group.

▼Effect direction toward better outcome in the control than in the intervention group.

●Null effect where the difference between the effect in the intervention and the control groups is 0.0; ..: not reported; N/A: effect estimates not reported, thus effect direction could not be derived.

^a^ Statistically significant postintervention through *p*‐value or CI.

^b^ Statistically significant in difference in means between groups (DiD) through *p*‐value or CI.

^c^ Statistical significance in neither *p*‐value nor CI is reported.

^d^ Physical activity parameters are grouped, and the statistical significance is shown if at least half the parameters were statistically significant.

^e^ Stages of dietary behavior and physical activity changes are illustrated from the results of the likelihood of change from the generalized linear mixed models (GLMMs).

Eleven RCTs [43, 44, 45, 46, 47, 48, 51, 52, 53, 56, 59] assessed lifestyle changes (*N* = 9 [44, 45, 46, 47, 48, 52, 53, 56, 59] regarding PA, *N* = 9 [43, 44, 45, 46, 47, 48, 51, 52, 53] regarding diet). Of these, effect directions in two RCTs reported increased PA [45, 48] and one overall improved diet [45], reductions in sweets consumption [45], alcohol consumption [45], sugar‐sweetened beverages [48], and sugar intake [53]. Effect directions in one study suggested increased salt intake in the intervention compared with the control group [43].

Six RCTs reported macronutrient intake effects (Appendix S12). They reported a decreasing tendency in carbohydrate intake/proportion [43, 44, 45, 46, 51, 53], protein intake/proportion [44, 46, 51, 53], total fat intake/proportion [43, 44, 46, 48, 51, 53], and fiber intake/proportion [46, 53]. Total fat intake/proportion and saturated fat intake/proportion increased in two RCTs [43, 46] compared with other RCTs where those outcomes decreased [44, 48, 51, 53].

Two RCTs [45, 47] reported on QoL outcomes, which mostly improved except for stress [45] and meridian body energy [47], where detrimental effects were reported.

Clinical outcomes not covered in the meta‐analyses due to less than two RCTs reporting on those outcomes included creatinine [53], Framingham 8‐year diabetes risk score [57], and traditional Chinese medicine parameters [47], with reductions for the first two outcomes and inconsistent effect directions for the last (Appendix S11).

The additional assessment of effect directions at different follow‐up times (Appendix S13) showed increased effects with a tendency toward the strongest effect estimates up to 6 months of follow‐up. Only two RCTs [50, 59] reported 12‐month follow‐ups, of which one study showed lower rates of participants reaching HbA_1c_ < 5.7% and > 5% weight loss [50] compared with earlier follow‐ups. For PA, effect directions suggested reduced mean steps/day, increased medium‐term, and reduced long‐term total PA in the intervention compared with the control group [59].

No outcomes were reported regarding diabetes incidence.

#### 3.4.2 Meta‐Analyses of Clinical Outcomes

We included results from 15 RCTs in at least one meta‐analysis. One study [48] reported raw means with no reply from the authors to our data request. Therefore, we calculated the pooled SE.

**FIGURE 2:**
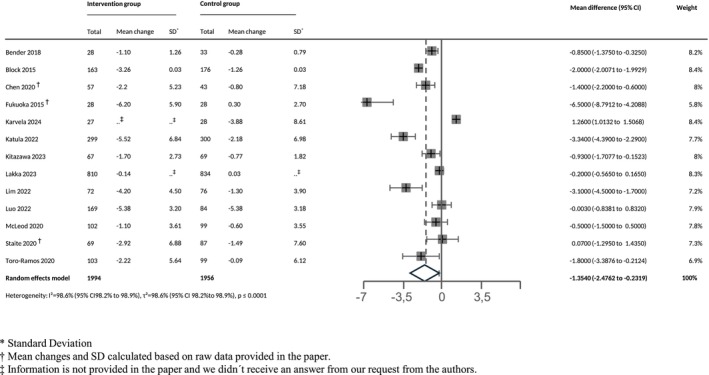
Effect of app‐based intervention on weight (in kg). *Standard deviation. †Mean changes and SD calculated based on raw data provided in the paper. ‡Information is not provided in the paper, and we did not receive an answer to our request to the authors.

We meta‐analyzed *N* = 19 clinical outcomes (Appendix S14) with the highest number of RCTs for body weight, followed by BMI and % HbA_1c_ (Figure 2). Pooled estimates showed statistically significant higher reductions in body weight in absolute (−1.35 kg, 95% CI: [−2.48; −0.23], *I* ^2^ = 99%, 95% CI: [98.2; 98.9], *N* = 13 RCTs) and relative terms (−2.68%, 95% CI: [−4.86; −0.50], *I* ^2^ = 90.4%, 95% CI: [81.8; 94.9], *N* = 6 RCTs), BMI (−0.53 kg/m^2^, 95% CI: [−0.97; −0.09], *I* ^2^ = 99%, 95% CI: [98.1; 98.8], *N* = 11 RCTs), and HbA_1c_ (−0.08%, 95% CI: [−0.10; −0.05], *I* ^2^ = 11%, 95% CI: [0.0; 51.2], *N* = 11 RCTs), with high heterogeneity for body weight and BMI. Pooled point estimates for most other meta‐analyzed outcomes suggested improved T2D risk factor profiles, although CIs were frequently compatible with null effects.

#### 3.4.3 Stratified Meta‐Analyses to Assess the Influence of Study Characteristics

**FIGURE 3:**
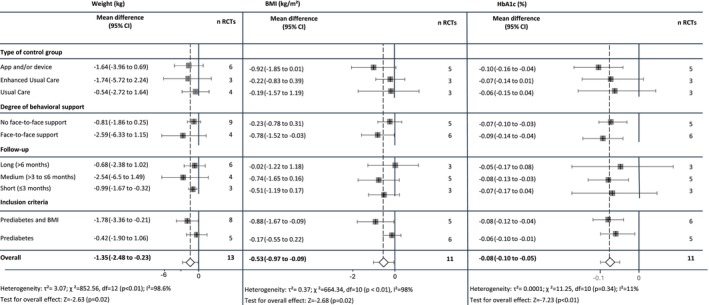
Stratified meta‐analyses of weight (in kg), BMI, and HbA1c (in %). Abbreviations: BMI—body mass index; HbA1c—glycated hemoglobin; RCT—randomized controlled trial.

Results of the stratified analyses based on study design characteristics, specifically on the type of control group, degree of behavioral support, follow‐up duration, inclusion criteria, and RoB, can be found in Figure 3 and Appendices S15–S24.

Through these stratified meta‐analyses, we found tendencies suggesting higher point estimates for interventions with f2f components for most outcomes compared to interventions without f2f components, although with overlapping CIs.

Concerning the type of control group (usual care vs. enhanced usual care vs. app/device), we found higher point estimates for comparisons against either control group receiving an app/device (for BMI and HbA_1c_) or enhanced UC (for weight), instead of usual care, again with overlapping CIs.

Stratifications by inclusion criteria (prediabetes vs. prediabetes plus high BMI) resulted in higher point estimates when populations were restricted to prediabetes plus high BMI for the outcomes weight, BMI, and HbA_1c_, also with overlapping CIs.

Stratified meta‐analyses by follow‐up measurement timepoints (short‐term [1 to ≤ 3 months], medium‐term [> 3 to ≤ 6 months], long‐term [> 6 months]) showed higher point estimates for weight, BMI, and HbA_1c_ for the 1 to ≤ 3 and > 3 to ≤ 6 months timepoints compared with > 6 months.

More granular plots of estimates over time showed no clear trends (Appendix S25). Comparing follow‐ups of < 12 and equal to 12 months confirmed larger effect estimates in follow‐ups < 12 months, specifically for anthropometric and blood glucose outcomes (Appendix S26).

RoB subgroup analyses did not substantially change the results (Appendices S15–S24) [58, 59].

#### 3.4.4 Descriptive Assessment of Health Equity‐Related Effect Heterogeneity

One RCT [57] reported effects stratified by ethnic background. For ethnic minorities, weight increased in the intervention group compared with the control group. Three RCTs [43, 46, 54] conducted subgroup analyses by gender and found no clear trends in terms of effect directions for different outcomes. One RCT [57] mentioned that the subgroup with higher educational degrees achieved less improvement in glycaemic markers than those with lower educational levels. Two RCTs [46, 50] conducted subgroup analyses by age groups and found improved effects in both subgroups, with larger effects for older compared with younger age groups for different clinical outcomes. Two RCTs [46, 54] stratified by overweight and obese populations found mixed effect directions for different outcomes. One RCT [55] found greater weight reductions with higher HbA_1c_ baseline ranges compared with lower ranges (Appendix S27).

### 3.5 Reporting Bias and Sensitivity Analyses

Our robustness checks via the leave‐one‐out method showed no substantial differences except for HbA_1c_ in mmol/mol, where removing one outlier [52] resulted in an increased and statistically significant overall effect estimate (Appendices S20 and S28). Changing assumptions for *r* in the calculation of the pooled effect estimate did not substantially change the results (Appendix S29). Egger's tests and funnel plots did not detect any risk of publication bias (Appendix S30).

## 4 Discussion

### 4.1 Summary of Results

This study provides a comprehensive synthesis of evidence from RCTs on the effectiveness of app‐based interventions in individuals with prediabetes. Overall, we found that app‐based interventions improved outcomes. This is reflected in our graphical synthesis of effect directions for effectiveness outcomes, where we observed mostly improved outcomes. Our meta‐analysis showed significant improvements in weight, BMI, and HbA_1c_. RCTs mostly reported clinical outcomes, followed by behavioral outcomes, whereas psychosocial and QoL outcomes were underrepresented. No RCT reported on diabetes incidence as an outcome. Stratified meta‐analyses showed that the largest effects were measured up to 6 months of follow‐up and decreased thereafter. Only a few RCTs reported follow‐up effects beyond 6 months, resulting in low power of our meta‐analyses of long‐term effectiveness. Additional stratified meta‐analyses suggested tendencies toward higher effects in comparisons against control interventions with f2f components, UC, and apps/devices and when high BMI was an additional inclusion criterion for the study population.

Most RCTs reported improved lifestyle behaviors in terms of diet and PA. However, two RCTs [43, 46] reported increased total fat intake/proportion and saturated fat intake/proportion. Both RCTs are distinguished from the others by personalized nutrition interventions.

Our synthesis of subgroup analyses related to equity was hindered by a lack of evidence as well as conflicting findings from included studies. Even when focusing on syntheses of effect directions, data remained sparse and largely inconclusive. This is unfortunate, as app‐based interventions may have the potential to either exacerbate or remedy existing health inequalities. More frequent reporting of subgroup analyses by more uniformly defined socioeconomic characteristics could therefore help assess the equity impact of app‐based interventions and inform policy decisions [60]. Only for the age effect directions suggested stronger improvements in outcomes among higher (age 50+) compared with younger age groups (Appendix S27) [46, 50].

### 4.2 Comparison With Previous Literature

Effect direction results supported previously reported positive associations between motivation [61], self‐efficacy [62], health literacy [63], and improved behaviors in the context of diabetes care [61, 64] and prevention [63]. Improved QoL outcomes have been shown previously in a community‐based lifestyle intervention study [65]. Our study suggests that this may also apply to app‐based interventions.

In one previous systematic review and meta‐analysis, six RCTs assessed the effectiveness of app‐based interventions in people with prediabetes [19]. This meta‐analysis has an overlap of four RCTs with ours but differs from ours by including RCTs whose respective authors postulated that populations were at high risk of developing T2D, whereas we only accepted populations that fulfilled standardized prediabetes criteria and meta‐analyzed a larger number of RCTs, probably due to a more comprehensive and later search. Furthermore, the authors only assessed the overall effectiveness, whereas we conducted stratified analyses related to relevant study design characteristics and explored effect heterogeneity related to equity. Our meta‐analysis suggested a smaller pooled effect estimate for weight reduction (−1.35 kg, 95% CI: [−2.48; −0.23], *I* ^2^ = 99%, *N* = 13 RCTs vs. −1.85 kg, 95% CI: [−2.90; −0.80], *I* ^2^ = 84%, *N* = 6 RCTs) and BMI (−0.53 kg/m^2^, 95% CI: [−0.97; −0.09], *I* ^2^ = 99%, *N* = 11 RCTs vs. −0.90 kg/m^2^, 95% CI: [−1.53; 0.27], *I* ^2^ = 84%, *N* = 6 RCTs) [19]. Regarding HbA_1c_, we found a statistically significant effect of −0.08% (95% CI: [−0.10; −0.05], *I* ^2^ = 11%, *N* = 11 RCTs), whereas Jahan et al. [19] suggested lower point estimates and compatibility with a null effect (−0.02%, 95% CI: [−0.64; 0.60], *N* = 6 RCTs). As the CIs of our pooled effects largely overlap with those of Jahan et al. [19], differences may result from sampling error and more included RCTs.

The results from our stratified meta‐analysis on the degree of behavioral support are in line with one meta‐analysis that assessed the effectiveness of lifestyle interventions delivered via eHealth [20], including one RCT overlap with our meta‐analysis, which found that eHealth interventions had a larger effect on weight loss with f2f (−4.65%, 95% CI: [−6.63; −2.67]) compared with no f2f support (−3.34%, 95% CI: [−4.00; −2.68]) [20]. Our statistically significant pooled effects for interventions compared with UC are in line with a meta‐analysis by Suhlrie et al. [60] that assessed the effectiveness of telemedicine interventions on populations with prediabetes. Bian et al. [21] assessed technology‐mediated interventions in a meta‐analysis, including one RCT overlap with our meta‐analysis, and found a weight reduction of −3.76 kg (95% CI: [−4.74; −2.77], *I* ^2^ = 96%) in populations with prediabetes or overweight. This stronger effect compared with our meta‐analysis might be explained by the intervention's focus on weight reduction. Our stratified meta‐analysis underlines this with increased weight reductions compared with overall effects in populations with increased BMI in addition to prediabetes.

Although we observed statistically significant effects only for up to 6 months follow‐up and no significant effects at 12 months follow‐up, only a few RCTs assessed effectiveness beyond 6 months. This is in accordance with one systematic review of systematic reviews that reported less long‐term evidence and pronounced effect maintenance compared with initial weight loss for eHealth interventions [66].

### 4.3 Strengths and Limitations

The strengths of this study include its high methodological standards and comprehensiveness. To our knowledge, no other systematic review and meta‐analysis is as comprehensive in assessing the effectiveness of app‐based interventions in individuals with prediabetes, including (1) syntheses of all available outcomes along the causal pathways, depicted in an ex ante developed literature‐based logic model (which allows drawing attention to well‐researched outcomes but also research gaps in effectiveness outcomes); (2) meta‐analyses on many clinical outcomes; (3) stratified meta‐analyses on study design characteristics; and (4) subgroup synthesis exploring effect heterogeneity related to equity.

This study has limitations, most stemming from the identified evidence base. First, our meta‐analysis still included a small number of RCTs and substantial heterogeneity for many outcomes, which limits precision. Second, heterogeneous measures resulted in considerable numbers of outcomes that we could not meta‐analyze. In such cases, we synthesized effect directions. Also, this lack of data only allowed us to conceptually outline causal pathways in a literature‐based logic model without being able to assess the effect sizes of individual pathways via mediation analyses. Third, we were not able to take intervention intensity into account because of both high heterogeneity and a lack of reported information. Finally, because of a lack of reporting in the underlying RCTs, we cannot draw definite conclusions regarding the effect of maintenance nor assess effects on T2D incidence.

### 4.4 Recommendations for Future Research

Multiple recommendations for future research can be drawn from our systematic review and meta‐analysis. First, we find it important to have long‐term follow‐up of app‐based interventions to better understand the impact on diabetes incidence and related diseases. Evidence from the original DPP showed an average weight loss of approximately 5 kg within 4 years and a reduced T2D incidence of 58%, 95% CI: [48; 66] [8]. Assuming the average weight loss of about 1.35 kg according to our meta‐analysis with a digital DPP could be sustained over the same time horizon, app‐based interventions may result in a modest T2D incidence reduction (about 12%).

Second, future studies should investigate potential treatment effect heterogeneity and its implications for health equity. Considering the digital divide, it will also be crucial to assess barriers to and facilitators of the application of such interventions among different populations. Most RCTs included in this study took place in high‐income countries, limiting external validity.

Third, we suggest further assessment of outcomes beyond clinical effectiveness. We found that RCTs mostly focused on clinical outcomes. A better understanding of psychosocial and behavioral effectiveness pathways may help improve interventions.

Fourth, as a large share (*n* = 7 RCTs [42, 48, 49, 50, 55, 57, 59]) out of the *n* = 18 RCTs included in our study mentioned some conceptual alignment with the DPP, we recommend further research focused on the most frequently used dDPPs to provide a comprehensive picture of the state of digital diabetes prevention policies that are already implemented as a public health measure in different health systems.

Lastly, our study included RCTs with innovative approaches such as AI‐supported personalized nutrition recommendations and CGM integrations. To leverage the potential of new data sources (i.e., CGM data), intensified research using advanced analytics will be crucial.

## 5 Conclusion

Overall, we found that app‐based interventions improved outcomes (i.e., lifestyle behavior, other process outcomes, QoL outcomes, and clinical outcomes). Our meta‐analyses suggest reductions in weight, BMI, and HbA_1c_ below the minimal clinically important differences in diabetes care (0.5% HbA_1c_ and 5% weight reductions) [67, 68]. However, we think that, in a preventative context, such effects may still produce a worthwhile impact at the population level [69]. The question of whether broader implementation of such interventions at the population scale is worthwhile will depend on their effectiveness in different population groups, long‐term impact on diabetes incidence, and per‐person costs.

## Author Contributions

A.J.S., L.S., M.L., J.B., and N.A. conceptualized the study. Data collection was carried out by L.S., N.A., and N.O.K. L.S. and N.A. performed the data analysis. J.B. and A.J.S. oversaw the data collection and analysis process. L.S. prepared the original manuscript draft. All authors contributed to editing and reviewing the final manuscript. All authors have read and approved the final version and the decision to submit. All authors had full access to the data, with L.S. and N.O.K. verifying its accuracy.

## Conflicts of Interest

The authors declare no conflicts of interest.

## Supporting information


**Appendix S1:** PRISMA 2020 checklist.
**Appendix S2:** PRISMA Abstract.
**Appendix S3:** Literature‐based Logic model 1, 2.
**Appendix S4:** Deviations from the prospectively registered protocol.
**Appendix S5:** Search strategy based on the population, intervention, comparison, and outcomes (PICO) framework.
**Appendix S6:** Formula of pooled standard errors with assumption on correlation coefficient.
**Appendix S7:** Excluded full‐text articles with reasons.
**Appendix S8:** Cohen's kappa interrater‐reliability coefficients.
**Appendix S9:** Risk of bias assessment.
**Appendix S10:** Details of effect directions analysis of non‐meta‐analyzed (process) outcomes.
**Appendix S11:** Effect directions of clinical outcomes of the longest follow‐up.
**Appendix S12:** Macronutrient intake effect descriptions of the longest follow‐up.
**Appendix S13:** Effect directions of (process) outcomes at different follow‐up times.
**Appendix S14:** Meta‐analyses results overview.
**Appendix S15:** Meta‐analysis SBP (mmHg).
**Appendix S16:** Meta‐analysis DBP (mmHg).
**Appendix S17:** Meta‐analysis FPG (mmol/L).
**Appendix S18:** Meta‐analysis HDL Cholesterol (mmol/L).
**Appendix S19:** Meta‐analysis total Cholesterol (mmol/L).
**Appendix S20:** Meta‐analysis triglycerides (mmol/L).
**Appendix S21:** Meta‐analysis HbA1c (mmol/mol).
**Appendix S22:** Meta‐analysis waist circumference (cm).
**Appendix S23:** Meta‐analysis LDL Cholesterol (mmol/L).
**Appendix S24:** Meta‐analysis Weight (%).
**Appendix S25:** Effect estimates over time.
**Appendix S26:** Sensitivity analysis of follow‐up effectiveness by < 12 months vs. 12 months.
**Appendix S27:** PROGRESS‐Plus effect directions.
**Appendix S28:** Leave‐one‐out forest plots.
**Appendix S29:** Sensitivity analysis Fukuoka et al.
**Appendix S30:** Egger's test results and funnel plots.

## Data Availability

This meta‐analysis utilized publicly available data previously reported in primary literature, which will be made available on OSF (DOI 10.17605/OSF.IO/B89QP) after publication.
