# Supplementary material for: Effectiveness of Smartphone Application‐Based Interventions to Prevent Type 2 Diabetes Mellitus in Individuals With Prediabetes: A Systematic Review and Meta‐Analysis
Source: Obes Rev. 2025 Oct 20;27(3):e70028. doi: 10.1111/obr.70028 (PMC12926618; doi:10.1111/obr.70028)
Supplement: Supplementary file 1 — Appendix S1: PRISMA 2020 checklist. Appendix S2: PRISMA Abstract. Appendix S3: Literature‐based Logic model 1, 2. Appendix S4: Deviations from the prospectively registered protocol. Appendix S5: Search strategy based on the population, intervention, comparison, and outcomes (PICO) framework. Appendix S6: Formula of pooled standard errors with assumption on correlation coefficient. Appendix S7: Excluded full‐text articles with reasons. Appendix S8: Cohen's kappa interrater‐reliability coefficients. Appendix S9: Risk of bias assessment. Appendix S10: Details of effect directions analysis of non‐meta‐analyzed (process) outcomes. Appendix S11: Effect directions of clinical outcomes of the longest follow‐up. Appendix S12: Macronutrient intake effect descriptions of the longest follow‐up. Appendix S13: Effect directions of (process) outcomes at different follow‐up times. Appendix S14: Meta‐analyses results overview. Appendix S15: Meta‐analysis SBP (mmHg). Appendix S16: Meta‐analysis DBP (mmHg). Appendix S17: Meta‐analysis FPG (mmol/L). Appendix S18: Meta‐analysis HDL Cholesterol (mmol/L). Appendix S19: Meta‐analysis total Cholesterol (mmol/L). Appendix S20: Meta‐analysis triglycerides (mmol/L). Appendix S21: Meta‐analysis HbA1c (mmol/mol). Appendix S22: Meta‐analysis waist circumference (cm). Appendix S23: Meta‐analysis LDL Cholesterol (mmol/L). Appendix S24: Meta‐analysis Weight (%). Appendix S25: Effect estimates over time. Appendix S26: Sensitivity analysis of follow‐up effectiveness by < 12 months vs. 12 months. Appendix S27: PROGRESS‐Plus effect directions. Appendix S28: Leave‐one‐out forest plots. Appendix S29: Sensitivity analysis Fukuoka et al. Appendix S30: Egger's test results and funnel plots. [file OBR-27-e70028-s001.pdf]

Effectiveness of smartphone application-based interventions to prevent type 2 diabetes mellitus in individuals with prediabetes: A systematic review and meta-analysis

Authors
Laura Suhlrie, MPH[1]
Nancy Abdelmalak, MSc[1]
Jacob Burns, PhD[1]
Hans Hauner, MD[2]
Niels Ole Kristiansen, MPH[1]
Anna-Janina Stephan, PhD[1, 3, *]
Michael Laxy, PhD[1, 3, *]

Affiliations
[1] Professorship of Public Health and Prevention
TUM School of Medicine and Health
Technical University of Munich, Germany

[2] Institute for Nutritional Medicine
TUM School of Medicine and Health
Technical University of Munich, Germany

[3] German Center for Diabetes Research (DZD)
Oberschleißheim, Germany

* Shared last authorship

SUPPLEMENTARY MATERIAL

Content

Appendix S1: PRISMA 2020 checklist 3

Appendix S2: PRISMA Abstract 5

Appendix S3: Literature-based Logic model [1, 2] 6

Appendix S4: Deviations from the prospectively registered protocol 6

Appendix S5: Search strategy based on the population, intervention, comparison, and outcomes (PICO) framework 6

Appendix S6: Formula of pooled standard errors with assumption on correlation coefficient 12

Appendix S7: Excluded full-text articles with reasons 13

Appendix S8: Cohen´s kappa interrater-reliability coefficients 14

Appendix S9: Risk of bias assessment 15

Appendix S10: Details of effect directions analysis of non-meta-analyzed outcomes 17

Appendix S11: Effect directions of clinical outcomes of longest follow-up 43

Appendix S12: Macronutrient intake effect descriptions of longest follow-up 44

Appendix S13: Effect directions of psychosocial and behavioural outcomes at different follow-up times 45

Appendix S14: Meta-analyses results overview 48

Appendix S15: Meta-analysis SBP (mmHg) 49

Appendix S16: Meta-analysis DBP (mmHg) 49

Appendix S17: Meta-analysis FPG (mmol/L) 50

Appendix S18: Meta-analysis HDL Cholesterol (mmol/L) 50

Appendix S19: Meta-analysis total Cholesterol (mmol/L) 51

Appendix S20: Meta-analysis triglycerides (mmol/L) 52

Appendix S21: Meta-analysis HbA1c (mmol/mol) 52

Appendix S22: Meta-analysis waist circumference (cm) 53

Appendix S23: Meta-analysis LDL Cholesterol (mmol/L) 54

Appendix S24: Meta-analysis Weight (%) 54

Appendix S25: Effect estimates over time 55

Appendix S26: Sensitivity analysis of follow-up effectiveness by <12 months vs. 12 months 56

Appendix S27: PROGRESS-Plus effect directions 58

Appendix S28: Leave-one-out forest plots 65

Appendix S29: Sensitivity analysis Fukuoka et al. 78

Appendix S30: Egger’s test results and funnel plots 79

References 80

Appendix S1: PRISMA 2020 checklist

Section and Topic	Item #	Checklist item	Location where item is reported in manuskript: Page(s)
**TITLE**			
Title	1	Identify the report as a systematic review.	1
**ABSTRACT**			
Abstract	2	See the PRISMA 2020 for Abstracts checklist.	2, Appendix S2
**INTRODUCTION**			
Rationale	3	Describe the rationale for the review in the context of existing knowledge.	3, 4
Objectives	4	Provide an explicit statement of the objective(s) or question(s) the review addresses.	4
**METHODS**			
Eligibility criteria	5	Specify the inclusion and exclusion criteria for the review and how studies were grouped for the syntheses.	4,5
Information sources	6	Specify all databases, registers, websites, organisations, reference lists and other sources searched or consulted to identify studies. Specify the date when each source was last searched or consulted.	4,5
Search strategy	7	Present the full search strategies for all databases, registers and websites, including any filters and limits used.	Appendix S5
Selection process	8	Specify the methods used to decide whether a study met the inclusion criteria of the review, including how many reviewers screened each record and each report retrieved, whether they worked independently, and if applicable, details of automation tools used in the process.	5
Data collection process	9	Specify the methods used to collect data from reports, including how many reviewers collected data from each report, whether they worked independently, any processes for obtaining or confirming data from study investigators, and if applicable, details of automation tools used in the process.	5
Data items	10a	List and define all outcomes for which data were sought. Specify whether all results that were compatible with each outcome domain in each study were sought (e.g. for all measures, time points, analyses), and if not, the methods used to decide which results to collect.	5-6
	10b	List and define all other variables for which data were sought (e.g. participant and intervention characteristics, funding sources). Describe any assumptions made about any missing or unclear information.	5-6
Study risk of bias assessment	11	Specify the methods used to assess risk of bias in the included studies, including details of the tool(s) used, how many reviewers assessed each study and whether they worked independently, and if applicable, details of automation tools used in the process.	6
Effect measures	12	Specify for each outcome the effect measure(s) (e.g. risk ratio, mean difference) used in the synthesis or presentation of results.	6, 7
Synthesis methods	13a	Describe the processes used to decide which studies were eligible for each synthesis (e.g. tabulating the study intervention characteristics and comparing against the planned groups for each synthesis (item #5)).	6, 7
	13b	Describe any methods required to prepare the data for presentation or synthesis, such as handling of missing summary statistics, or data conversions.	6, 7
	13c	Describe any methods used to tabulate or visually display results of individual studies and syntheses.	6, 7
	13d	Describe any methods used to synthesize results and provide a rationale for the choice(s). If meta-analysis was performed, describe the model(s), method(s) to identify the presence and extent of statistical heterogeneity, and software package(s) used.	6, 7
	13e	Describe any methods used to explore possible causes of heterogeneity among study results (e.g. subgroup analysis, meta-regression).	7
	13f	Describe any sensitivity analyses conducted to assess robustness of the synthesized results.	7, 8; Appendix S6; Appendix S26; Appendix S28-S30

Section and Topic	Item #	Checklist item	Location where item is reported in manuskript: Page(s)
Reporting bias assessment	14	Describe any methods used to assess risk of bias due to missing results in a synthesis (arising from reporting biases).	8
Certainty assessment	15	Describe any methods used to assess certainty (or confidence) in the body of evidence for an outcome.	6-8
**RESULTS**			
Study selection	16a	Describe the results of the search and selection process, from the number of records identified in the search to the number of studies included in the review, ideally using a flow diagram.	8, 20: Figure 1
	16b	Cite studies that might appear to meet the inclusion criteria, but which were excluded, and explain why they were excluded.	Appendix S7
Study characteristics	17	Cite each included study and present its characteristics.	21-27: Table 1
Risk of bias in studies	18	Present assessments of risk of bias for each included study.	8, Appendix S9
Results of individual studies	19	For all outcomes, present, for each study: (a) summary statistics for each group (where appropriate) and (b) an effect estimate and its precision (e.g. confidence/credible interval), ideally using structured tables or plots.	8-9, Figure 2-3; Appendix S15-24
Results of syntheses	20a	For each synthesis, briefly summarise the characteristics and risk of bias among contributing studies.	8; App. Figure S9
	20b	Present results of all statistical syntheses conducted. If meta-analysis was done, present for each the summary estimate and its precision (e.g. confidence/credible interval) and measures of statistical heterogeneity. If comparing groups, describe the direction of the effect.	8-11, Figure 2, 3, 4; Appendix S15-24
	20c	Present results of all investigations of possible causes of heterogeneity among study results.	8-11, Figure 2; Appendix S15-24, 27
	20d	Present results of all sensitivity analyses conducted to assess the robustness of the synthesized results.	11
Reporting biases	21	Present assessments of risk of bias due to missing results (arising from reporting biases) for each synthesis assessed.	11
Certainty of evidence	22	Present assessments of certainty (or confidence) in the body of evidence for each outcome assessed.	11, Appendix S29, Appendix S30
**DISCUSSION**			
Discussion	23a	Provide a general interpretation of the results in the context of other evidence.	11-13
	23b	Discuss any limitations of the evidence included in the review.	13
	23c	Discuss any limitations of the review processes used.	13
	23d	Discuss implications of the results for practice, policy, and future research.	13, 14
**OTHER INFORMATION**			
Registration and protocol	24a	Provide registration information for the review, including register name and registration number, or state that the review was not registered.	2, 4
	24b	Indicate where the review protocol can be accessed, or state that a protocol was not prepared.	2, 4
	24c	Describe and explain any amendments to information provided at registration or in the protocol.	Appendix S4
Support	25	Describe sources of financial or non-financial support for the review, and the role of the funders or sponsors in the review.	1
Competing interests	26	Declare any competing interests of review authors.	1
Availability of data, code and other materials	27	Report which of the following are publicly available and where they can be found: template data collection forms; data extracted from included studies; data used for all analyses; analytic code; any other materials used in the review.	15

Appendix S2: PRISMA Abstract

Section and Topic	Item #	Checklist item	Reported (Yes/No)
**TITLE**			
Title	1	Identify the report as a systematic review.	Yes
**BACKGROUND**			
Objectives	2	Provide an explicit statement of the main objective(s) or question(s) the review addresses.	Yes
**METHODS**			
Eligibility criteria	3	Specify the inclusion and exclusion criteria for the review.	Yes
Information sources	4	Specify the information sources (e.g. databases, registers) used to identify studies and the date when each was last searched.	Yes
Risk of bias	5	Specify the methods used to assess risk of bias in the included studies.	Yes
Synthesis of results	6	Specify the methods used to present and synthesise results.	Yes
**RESULTS**			
Included studies	7	Give the total number of included studies and participants and summarise relevant characteristics of studies.	Yes
Synthesis of results	8	Present results for main outcomes, preferably indicating the number of included studies and participants for each. If meta-analysis was done, report the summary estimate and confidence/credible interval. If comparing groups, indicate the direction of the effect (i.e. which group is favoured).	Yes
**DISCUSSION**			
Limitations of evidence	9	Provide a brief summary of the limitations of the evidence included in the review (e.g. study risk of bias, inconsistency and imprecision).	Yes
Interpretation	10	Provide a general interpretation of the results and important implications.	Yes
**OTHER**			
Funding	11	Specify the primary source of funding for the review.	Yes
Registration	12	Provide the register name and registration number.	Yes

From: Page MJ, McKenzie JE, Bossuyt PM, Boutron I, Hoffmann TC, Mulrow CD, et al. The PRISMA 2020 statement: an updated guideline for reporting systematic reviews. BMJ 2021;372:n71. doi: 10.1136/bmj.n71. This work is licensed under CC BY 4.0. To view a copy of this license, visit https://creativecommons.org/licenses/by/4.0/

Appendix S3: Literature-based Logic model [1, 2]

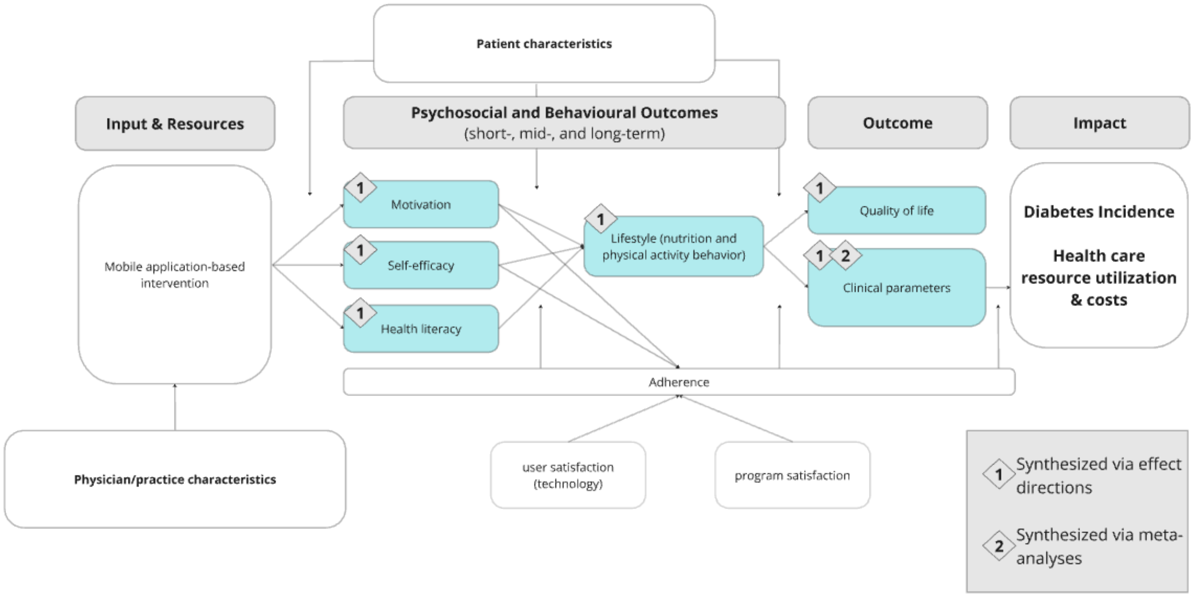

Appendix S4: Deviations from the prospectively registered protocol

Specifications in the protocol	Deviations from the protocol
The protocol covers a larger literature synthesis project which aims at assessing app-based interventions to prevent T2D with regard to various evidence dimensions including effectiveness, reach, equity, adoption, implementation, maintenance, and cost-effectiveness	While this publication focuses on the project results for effectiveness, we aim to address further dimensions in a separate publication to allow for adequate space and consideration of all evidence dimensions.
We pre-specified in the protocol to do a subgroup analysis based on the duration of intervention.	We noticed a strong correlation with the subgroup analysis on follow-up time points and therefore, decided to only keep the follow-up time points subgroup analysis.
No pre-specified subgroup analysis was defined for type of inclusion criteria.	Based on the observed dichotomy of RCTs with regard to inclusion criteria that we found when extracting the respective data (either prediabetes or pre-diabetes plus high BMI), we added a post-hoc subgroup analysis on the type of inclusion criteria

Appendix S5: Search strategy based on the population, intervention, comparison, and outcomes (PICO) framework

PubMedCentral and MEDLINE via Pubmed (search completed on 29 Jan 24)

Population

#	Searches	Results
1	prediabet*[Title/Abstract]<br>OR pre-diabet*[Title/Abstract]<br>OR prediabetic state[MeSH]	17,149
2	Diabetes Mellitus, Type 2[MeSH Terms]<br>OR diabet*[Title/Abstract]	817,845
3	metabolic syndrome[Title/Abstract]<br>OR metabolic syndrome[MeSH Terms]	72,347
4	Hyperglyc* [Title/Abstract]<br>OR hyperglycemia[MeSH Terms]	97,301
5	Impaired fasting glucose[Title/Abstract]<br>OR Impaired glucose tolerance[Title/Abstract]<br>OR Impaired glucose level*[Title/Abstract]<br>OR glucose intolerance[Title/Abstract]	25,564
6	Insulin resistance[Title/Abstract]	102,021
7	High blood sugar[Title/Abstract]<br>OR abnormal blood glucose level[Title/Abstract]	505
8	dysglycaemia[Title/Abstract]	2,126

	OR dysglycemia[Title/Abstract]	
9	risk population[Title/Abstract]	15,075
10	type 1[Title/Abstract]<br>OR gestational diabetes[Title/Abstract]<br>OR NAFLD[Title/Abstract]<br>OR neuropath*[Title/Abstract]<br>OR kidney[Title/Abstract]<br>OR metformin[Title/Abstract]<br>OR diabetes self management[Title/Abstract]<br>OR self management of diabetes[Title/Abstract]<br>OR diabetes management[Title/Abstract]<br>OR management of diabetes[Title/Abstract]<br>OR medication management[Title/Abstract]<br>OR medication adherence[Title/Abstract]<br>OR diabetes medication[Title/Abstract]<br>OR diabetes control[Title/Abstract]<br>OR patients with type 2 diabetes[Title/Abstract]<br>OR diabetic patient*[Title/Abstract]<br>OR PCOS[Title/Abstract]<br>OR polycystic ovary syndrome[Title/Abstract]<br>OR diabetic retinopathy[Title/Abstract]	1,117,566
11	#1 OR #2 OR #3 OR #4 OR #5 OR #6 OR #7 OR #8 OR #9 NOT #10	629,711

Smartphone app-based intervention

#	Searches	Results
12	mobile applications[MeSH Terms]<br>OR mobile application*[Title/Abstract]	16,029
13	smartphone application*[Title/Abstract]<br>OR smart phone application*[Title/Abstract]	4,060
14	mobile phone application*[Title/Abstract]<br>OR mobilephone application*[Title/Abstract]	668
15	(app[Title/Abstract] NOT amyloid[Title/Abstract])<br>OR (apps[Title/Abstract] NOT amyloid[Title/Abstract])	34,706
16	Smart phone based[Title/Abstract]<br>OR smartphone based[Title/Abstract]	3,863
17	mobile phone based[Title/Abstract]<br>OR mobilephone based[Title/Abstract]	853
18	application based[Title/Abstract]	1,503
19	digital[Title/Abstract]	193,168
20	mobile health[Title/Abstract]<br>OR mhealth[Title/Abstract]<br>OR m-health[Title/Abstract]	15,337
21	computers, handheld[MeSH Terms]	13,571
22	#12 OR #13 OR #14 OR #15 OR #16 OR #17 OR #18 OR #19 OR #20 OR #21	246,754

Prevention

#	Searches	Results
23	prevent*[Title/Abstract]	1,831,110
24	risk reduction behavior[Title/Abstract]<br>OR Risk Reduction Behavior[Mesh:NoExp]	15,099
25	Life style [Mesh]<br>OR Life Style[Title/Abstract]	120,906
26	Intervention*[Title/Abstract]<br>OR Program*[Title/Abstract]	2,336,782
27	diet*[Title/Abstract]<br>OR Diet, Reducing[Mesh]	714,791
28	nutrition*[Title/Abstract]	382,289
29	Bodyweight/Body Weight Changes[majr]<br>OR Bodyweight/overweight[majr]<br>OR weight[Title/Abstract]	1,001,801
30	exercis*[Title/Abstract]<br>OR physical activity[Title/Abstract]	489,727
31	behavi* change[Title/Abstract]	173,964
32	Preventive Health Services[Mesh:NoExp]	14,501
33	Primary Prevention[Mesh:NoExp]<br>OR Primary Prevention[Title/Abstract]	38,405
34	Health Promotion[Title/Abstract]<br>OR Health Promotion[Mesh:NoExp]	107,341
35	Body Mass Index[Title/Abstract]<br>OR Body Mass Index[Mesh]	308,539

36	Glycated Hemoglobin [Title/Abstract]<br>OR Glycated Hemoglobin[Mesh]	51,010
37	Waist Circumference[Mesh:NoExp]<br>OR waist circumference[Title/Abstract]<br>OR Waist-Hip Ratio[Mesh]<br>OR Waist-hip ratio[Title/Abstract]	42,992
38	Blood Glucose[Mesh]<br>OR blood glucose[Title/Abstract]	231,131
39	#23 OR #24 OR #25 OR #26 OR #27 OR #28 OR #29 OR #30 OR #31 OR #32 OR #33 OR #34 OR #35 OR #36 OR #37 OR #38	6,034,729

40	#11 AND #22 AND #39	**1741**

<u>Web of Science</u> (search completed on 29 Jan 24)

Population

#	Searches	Results
1	TS=”prediabet*”<br>OR TS=”pre-diabet*”	15,054
2	TS=”diabet*”<br>OR TS=”T2D*”	907,054
3	TS=”metabolic syndrome”	111,609
4	TS=”Hyperglyc$emi*”	87,029
5	TS=”Impaired fasting glucose”<br>OR TS=”Impaired glucose tolerance”<br>OR TS=”Impaired glucose level*”<br>OR TS=”glucose intolerance”	33,150
6	TS=”Insulin resistance”	176,848
7	TS=”High blood sugar”<br>OR TS=”abnormal blood glucose level”	468
8	TS=”dysglyc$emia”	2,193
9	TS=”risk population”	13,888
10	TS=”type 1”<br>OR TS=”gestational diabetes”<br>OR TS=”NAFLD”<br>OR TS=”neuropath*”<br>OR TS=”kidney”<br>OR TS=”metformin”<br>OR TS=”diabetes self management”<br>OR TS=”self management of diabetes”<br>OR TS=”self management of type 2 diabetes”<br>OR TS=”diabetes management”<br>OR TS=”management of diabetes”<br>OR TS=”management of type 2 diabetes”<br>OR TS=”medication management”<br>OR TS=”medication adherence”<br>OR TS=”diabetes medication”<br>OR TS=”diabetes control”<br>OR TS=”patients with type 2 diabetes”<br>OR TS=”patients with diabetes type 2”<br>OR TS=”diabetic patient*”<br>OR TS=”PCOS”<br>OR TS=”polycystic ovary syndrome”<br>OR TS= “diabetic retinopathy”	1,289,173
11	#1 OR #2 OR #3 OR #4 OR #5 OR #6 OR #7 OR #8 OR #9 NOT #10	719,166

Smartphone App-based intervention

#	Searches	Results
12	TS=”mobile app*”	35,106
13	TS=”smartphone app*”<br>OR TS=”smart-phone app*”	11,421
14	TS=”mobile-phone app*”<br>OR TS=”mobilephone app*”	2,155
15	TS=”smart-phone based”<br>OR TS=”smartphone based”	6,752
16	TS=”mobile-phone based”<br>OR TS=”mobilephone based”	1,155
17	TS=”application based”	7,185
18	TS=“digital“	727,791

19	TS=”mobile health”<br>OR TS=”mhealth”<br>OR TS=”m-health”	16,668
20	#12 OR #13 OR #14 OR #15 OR #16 OR #17 OR #18 OR #19	791,435

Prevention

#	Searches	Results
21	TS=”prevent*”	2,133,511
22	TS=”risk reduction”	33,725
23	TS=”Life style”<br>OR TS=”Lifestyle”	160,962
24	TS=”Intervention*”<br>OR TS=”Program*”	3,486,085
25	TS=“diet*“	991,314
26	TS=“nutrition*“	517,582
27	TS=”bodyweight”<br>OR TS=”overweight”<br>OR TS=”weight”	1,658,853
28	TS=”exercis*”<br>OR TS=”physical activity”	689,755
29	TS=”behavi* change”	36,963
30	TS=”preventive health service*”	1,218
31	TS=”Primary Prevention”	27,049
32	TS=”Health Promotion”	44,015
33	TS=”Body Mass Index”	261,157
34	TS=”Glycated Hemoglobin”	12,324
35	TS=”Waist Circumference”<br>OR TS=”Waist-Hip Ratio”	38,674
36	TS=”Blood Glucose”	86,840
37	#21 OR #22 OR #23 OR #24 OR #25 OR #26 OR #27 OR #28 OR #29 OR #30 OR #31 OR #32 OR #33 OR #34 OR #35 OR #36	8,204,071

38	#11 AND #20 AND #37	**1,580**

Scopus (search completed on 31 Jan 24)

Population

#	Searches	Results
1	title-abs-key (prediabet*)<br>OR title-abs-key (pre-diabet*)	19,063
2	title-abs-key (diabet*)	1,288,646
3	title-abs-key ({metabolic syndrome})	104,142
4	title-abs-key (Hyperglyc*emi*)	146,906
5	title-abs-key ({Impaired fasting glucose})<br>OR title-abs-key ({Impaired glucose tolerance})<br>OR title-abs-key (“Impaired glucose level”)<br>OR title-abs-key ({glucose intolerance})	55,681
6	title-abs-key ({Insulin resistance})	166,267
7	title-abs-key ({High blood sugar})<br>OR title-abs-key (“abnormal blood glucose level”)	1,050
8	title-abs-key (dysglyc*emia)	3,068
9	title-abs-key ({risk population})	137,559
10	title-abs-key ({type 1})<br>OR title-abs-key ({gestational diabetes})<br>OR title-abs-key (NAFLD)<br>OR title-abs-key (“neuropath*”)<br>OR title-abs-key (kidney)<br>OR title-abs-key (metformin)<br>OR title-abs-key (management w/5 diabetes)<br>OR title-abs-key ({medication management})<br>OR title-abs-key ({medication adherence})<br>OR title-abs-key ({diabetes medication})<br>OR title-abs-key ({diabetes control})<br>OR title-abs-key ({patients with type 2 diabetes})<br>OR title-abs-key ({patients with diabetes type 2})<br>OR title-abs-key (“diabet* patient”)<br>OR title-abs-key (PCOS)<br>OR title-abs-key ({polycystic ovary syndrome})	2,400,506

11	#1 OR #2 OR #3 OR #4 OR #5 OR #6 OR #7 OR #8 OR #9 AND NOT #10	1,024,927

Smartphone App-based intervention

#	Searches	Results
12	title-abs-key (“mobile application”)	61,758
13	title-abs-key (“smart*phone application”)	8,749
14	title-abs-key (“mobile*phone application”)	3
15	title-abs-key (“app”) AND NOT title-abs-key (“amyloid”)	97,821
16	title-abs-key (“smart*phone based”)	8,473
17	title-abs-key (“mobile*phone based”)	3
18	title-abs-key ({application based})	8,131
19	title-abs-key (digital)	1,588,538
20	title-abs-key ({mobile health})<br>OR title-abs-key (“m*health”)	31,662
21	#12 OR #13 OR #14 OR #15 OR #16 OR #17 OR #18 OR #19 OR #20	1,743,624

Prevention

#	Searches	Results
22	title-abs-key (prevent*)	3,371,500
23	title-abs-key ({risk reduction})	157,559
24	title-abs-key (“Life*style”)	310,892
25	title-abs-key (Intervention*)<br>OR title-abs-key (Program*)	6,023,985
26	title-abs-key (diet*)	1,488,559
27	title-abs-key (nutrition*)	864,037
28	title-abs-key (Bodyweight)<br>OR title-abs-key (overweight)<br>OR title-abs-key (weight)	3,074,992
29	title-abs-key (exercis*)<br>OR title-abs-key ({physical activity})	1,017,466
30	title-abs-key (“behavi* change”)	102,989
31	title-abs-key ({Preventive health services})	14,100
32	title-abs-key ({Primary Prevention})	63,821
33	title-abs-key ({Health Promotion})	158,555
34	title-abs-key ({Body Mass Index})	326,868
35	title-abs-key ({Glycated Hemoglobin})	26,844
36	title-abs-key ({Waist Circumference})<br>OR title-abs-key ({Waist-Hip Ratio})	69,377
37	title-abs-key ({Blood Glucose})	256,586
38	#23 OR #24 OR #25 OR #26 OR #27 OR #28 OR #29 OR #30 OR #31 OR #32 OR #33 OR #34 OR #35 OR #36 OR #37	13,907,580

39	#11 AND #21 AND #38	**2,720**

<u>EMBASE via Ovid</u> (search completed on 30 Jan 24)

Population

#	Searches	Results
1	prediabet*.ti,ab,kf.<br>OR pre-diabet*.ti,ab,kf.<br>OR exp impaired glucose tolerance/	44,305
2	exp diabetes mellitus/<br>OR diabet*.ti,ab,kf.	1,421,134
3	metabolic syndrome.ti,ab,kf.<br>OR exp metabolic syndrome X/	128,251
4	Hyperglyc*.ti,ab,kf.<br>OR exp hyperglycemia/	155,498
5	Impaired fasting glucose.ti,ab,kf.<br>OR Impaired glucose tolerance.ti,ab,kf.<br>OR Impaired glucose level*.ti,ab,kf.<br>OR glucose intolerance.ti,ab,kf.	37,524
6	Insulin resistance.ti,ab,kf.	149,509
7	High blood sugar.ti,ab,kf.<br>OR abnormal blood glucose level.ti,ab,kf.	854

8	dysglycaemia.ti,ab,kf.<br>OR dysglycemia.ti,ab,kf.	3,506
9	risk population.ti,ab,kf.	24,567
10	type 1.ti,ab,kf.<br>OR gestational diabetes.ti,ab,kf.<br>OR NAFLD.ti,ab,kf.<br>OR neuropath*.ti,ab,kf.<br>OR kidney.ti,ab,kf.<br>OR metformin.ti,ab,kf.<br>OR diabetes self management.ti,ab,kf.<br>OR self management of diabetes.ti,ab,kf.<br>OR diabetes management.ti,ab,kf.<br>OR management of diabetes.ti,ab,kf.<br>OR medication management.ti,ab,kf.<br>OR medication adherence.ti,ab,kf.<br>OR diabetes medication.ti,ab,kf.<br>OR diabetes control.ti,ab,kf.<br>OR patients with type 2 diabetes.ti,ab,kf.<br>OR diabetic patient*.ti,ab,kf.<br>OR PCOS.ti,ab,kf.<br>OR polycystic ovary syndrome.ti,ab,kf.<br>OR diabetic retinopathy.ti,ab,kf.	1,517,283
11	1 OR 2 OR 3 OR 4 OR 5 OR 6 OR 7 OR 8 OR 9 NOT 10	1,092,877

Smartphone App-based intervention

#	Searches	Results
12	exp mobile applications/<br>OR mobile application*.ti,ab,kf.	28,776
13	smartphone application*.ti,ab,kf.<br>OR smart phone application*.ti,ab,kf.	5,690
14	mobile-phone application*.ti,ab,kf.<br>OR mobilephone application*.ti,ab,kf.	873
15	app.ti,ab,kf.	55,205
16	amyloid.ti,ab,kf.	136,528
17	15 NOT 16	37,373
18	apps.ti,ab,kf.	14,633
19	amyloid.ti,ab,kf.	136,528
20	18 NOT 19	14,117
21	Smart phone based.ti,ab,kf.<br>OR smartphone based.ti,ab,kf.	4,610
22	mobile phone based.ti,ab,kf.<br>OR mobilephone based.ti,ab,kf.	917
23	application based.ti,ab,kf.	1,765
24	digital.ti,ab,kf.	231,501
25	mobile health.ti,ab,kf.<br>OR mhealth.ti,ab,kf.<br>OR m-health.ti,ab,kf.	14,797
26	exp personal digital assistant/	1,857
27	12 OR 13 OR 14 OR 17 OR 20 OR 21 OR 22 OR 23 OR 24 OR 25	294,636

Prevention

#	Searches	Result
28	prevent*.ti,ab,kf.	2,246,932
29	risk reduction.ti,ab,kf.<br>OR risk reduction/	147,858
30	exp lifestyle/<br>OR Life Style.ti,ab,kf.	174,156
31	Intervention*.ti,ab,kf.<br>OR Program*.ti,ab,kf.	3,046,284
32	diet*.ti,ab,kf.<br>OR exp diet/	885,843
33	nutrition*.ti,ab,kf.	458,228
34	Bodyweight.ti,ab,kf.<br>OR Body Weight Changes.ti,ab,kf.<br>OR Bodyweight.ti,ab,kf.<br>OR overweight.ti,ab,kf.<br>OR weight.ti,ab,kf.	1,308,998
35	exercis*.ti,ab,kf.<br>OR physical activity.ti,ab,kf.	616,574
36	behavi* change.ti,ab,kf.	34,513

37	preventive health services/	28,854
38	primary prevention/<br>OR Primary Prevention.ti,ab,kf.	60,977
39	Health Promotion.ti,ab,kf.<br>OR health promotion/	128,920
40	Body Mass Index.ti,ab,kf.<br>OR exp body mass/	709,860
41	Glycated Hemoglobin .ti,ab,kf.<br>OR exp glycated hemoglobin/	165,328
42	Waist Circumference/<br>OR waist circumference.ti,ab,kf.<br>OR exp waist hip ratio/<br>OR Waist-hip ratio.ti,ab,kf.	94,707
43	exp blood glucose/<br>OR blood glucose.ti,ab,kf.	344,686
44	28 OR 29 OR 30 OR 31 OR 32 OR 33 OR 34 OR 35 OR 36 OR 37 OR 38 OR 39 OR 40 OR 41 OR 42 OR 43	7,637,623
45	11 AND 27 AND 44	**3,662**

➔ **Total: 9703**

Additional note: LS hand searched clinicaltrials.gov and the Cochrane Database of Systematic Reviews (CDSR) trial registries for overlooked projects. This hand search did not yield any results.

Appendix S6: Formula of pooled standard errors with assumption on correlation coefficient

To calculate estimators for the meta-analysis from provided mean differences we used the following formulas as outlined by the Cochrane Handbook and Goryakin et al. (2018) [3, 4].

Difference-in-difference (DiD) estimators:

$$DiD = \bar{\mu}_1 - \bar{\mu}_2,$$

where $\bar{\mu}_1$ and $\bar{\mu}_2$ are average within-group differences between baseline and follow-up, for intervention and control groups, respectively.

Standard error (SE) of the DiD:

$$\mathrm{SE}(DiD) = S_p\sqrt{\frac{1}{n_1} + \frac{1}{n_2}},$$

Where S_p is the estimate of the pooled standard deviation (SD) for the intervention and control groups.

Common standard deviation for the intervention and control groups:

$$S_p = \sqrt{\frac{(n_1 - 1)s_1^2 + (n_2 - 1)s_2^2}{n_1 + n_2 - 2}},$$

where n_1 and n_2 are sample sizes for the intervention and control groups; s_1 and s_2 are the estimates of the SDs of the within-group differences between baseline and follow-up for intervention and control groups, respectively.

One study only provided raw means at baseline and follow-up. Therefore, we calculated the standard deviation of the within-group difference between baseline and follow-up as follows (for intervention and control groups separately):

$$SD_{E,change} = \sqrt{SD_{E,baseline}^2 + SD_{E,final}^2 - (2 \times Corr \times SD_{E,baseline} \times SD_{E,final})},$$

where $SD_{E,\ baseline}$ and $SD_{E,\ final}$ are SDs for the underlying outcome variable (E) at baseline and follow-up, respectively; Corr is the correlation coefficient between measurements at baseline and follow-up. Since the Corr values are unknown, we assumed autocorrelation in the respective outcomes (HbA1c, SBP, DBP, Triglycerides, FPG, HDL Cholesterol, LDL Cholesterol, Total Cholesterol) of Corr=0.90. We conducted sensitivity analyses with Corr=0.7; 0.8; 0.95 to assess the impact of this assumption on our results.

Appendix S7: Excluded full-text articles with reasons

No	Title	First author	Year	Reason for exclusion[1]
1	Telemedicine as a supplement to regular visits - Evaluation via a multicenter randomized controlled study	Adolfsson	2019	Not peer-reviewed
2	Using Mhealth Apps for Behaviour Change in Urban and Rural India -a Pilot Study	Anjana	2022	Not peer-reviewed
3	Engagement in Digital Health App-Based Prevention Programs Is Associated With Weight Loss Among Adults Age 65+	Auster-Gussman	2022	No RCT
4	Gut microbiome modulates the effects of a personalised postprandial-targeting (PPT) diet on cardiometabolic markers: a diet intervention in pre-diabetes	Ben-Yacov	2023	Publication related to included RCT
5	The Impact of A Personalized Weight Loss Diet on Dietary Measures in Adults with Prediabetes and Early-stage Type 2 Diabetes	Berube	2022	Not peer-reviewed
6	Fully-automated online diabetes prevention program Alive-PD is effective in lowering HbA1c glucose and weight	Block	2015	Not peer-reviewed
7	The Effects of Text Messages for Promoting Physical Activities in Prediabetes: A Randomized Controlled Trial	Bootwong	2022	No mobile-application based intervention
8	Correction to: The Effects of Mobile-App-Based Low-Carbohydrate Dietary Guidance on Postprandial Hyperglycemia in Adults with Prediabetes (Diabetes Therapy	Chen	2020	Correction
9	The Effects of Mobile-App-Based Low-Carbohydrate Dietary Guidance on Postprandial Hyperglycemia in Adults with Prediabetes	Chen	2020	Duplicate with included article
10	Satisfaction with a digitally-enabled telephone health coaching intervention for people with non-diabetic hyperglycaemia	Coventry	2019	No mobile-application based intervention
11	A novel mobile phone delivered diabetes prevention program in overweight adults at risk for type 2 diabetes-a randomized controlled trial	Fukuoka	2014	Not peer-reviewed
12	A Novel Diabetes Prevention Intervention Using a Mobile App	Fukuoka	2015	Duplicate
13	A weight loss intervention using a commercial mobile application in Latino Americans-Adelgaza Trial	Fukuoka	2018	No RCT
14	A randomized clinical trial of mhealth supported exercise intervention in patients with metabolic syndrome	Gill	2013	Not peer-reviewed
15	Using a smartphone app in changing cardiovascular risk factors: A randomized controlled trial (EVIDENT II study)	Gonzalez-Sanchez	2019	No RCT
16	Behavioral lifestyle intervention program using mobile application improves diet quality in adults with prediabetes (D'LITE study): a randomized controlled trial	Han	2023	Publication related to included RCT
17	Effectiveness of a complex pre-conception intervention to reduce the risk of diabetes by reducing adiposity in young adults in Malaysia: The Jom Mama project â€“ A randomised controlled trial"	Hanafiah	2022	No prediabetes
18	Internet-Based Lifestyle Intervention to Prevent Type 2 Diabetes Through Healthy Habits: Design and 6-Month Usage Results of Randomized Controlled Trial	Harjumaa	2020	Publication related to included RCT
19	Telemedicine-Based Health Coaching Is Effective for Inducing Weight Loss and Improving Metabolic Markers	Johnson	2019	No prediabetes
20	Effects of a digital diabetes prevention program on hba1c and body weight in prediabetes	Katula	2020	Not peer-reviewed
21	A randomized clinical trial comparing low-fat with precision nutritionâ€“based diets for weight loss: impact on glycemic variability and HbA1c	Kharmats	2023	No prediabetes
22	Behavioural interventions to promote physical activity in a multiethnic population at high risk of diabetes: PROPELS three-arm RCT	Khunti	2021	No mobile-application based intervention
23	Promoting physical activity in a multi-ethnic population at high risk of diabetes: the 48-month PROPELS randomised controlled trial	Khunti	2021	No mobile-application based intervention
24	Promoting long-term physical activity in prediabetes: The propels RCT	Khunti	2020	Not peer-reviewed
25	Effects of a mobile healthcare service provided by public health centers on practicing of health behaviors and health risk factors	Kim	2019	No prediabetes
26	Dietary Sodium Reduction with App-Based Education Program for People with Hypertension and Pre-Diabetes: Results of a Pilot Study	Kim	2023	Not peer-reviewed
27	Lifestyle Intervention Program with Smartphone App Augmented by Intermittently Scanned Continuous Glucose Monitoring for People at High Risk of Diabetes-Randomized Controlled Trial	Kitazawa	2023	Not peer-reviewed
28	Mobile health physical activity and obesity: Subanalysis of a randomized controlled trial	Lee	2018	No RCT

29	Comparing Digital Therapeutic Intervention with an Intensive Obesity Management Program: Randomized Controlled Trial	Moravcova	2019	No prediabetes [2]
30	Change in cardiometabolic risk factors among Asian Indian adults recruited in a mHealth-based diabetes prevention trial	Muralidharan	2021	No prediabetes [2]
31	Engagement and Weight Loss: Results from the Mobile Health and Diabetes Trial	Muralidharan	2019	No prediabetes [2]
32	The effect of healthcare service of employees at a workplace using mobile	Noh	2020	No RCT
33	Effect of novel technology-enabled multidimensional physical activity feedback in primary care patients at risk of chronic disease - The MIPACT study: A randomised controlled trial	Peacock	2020	No prediabetes
34	Mobile health exercise and metabolic risk: a randomized controlled trial"	Petrella	2014	No prediabetes
35	The Effects of Time-Restricted Eating versus Standard Dietary Advice on Weight	Phillips	2021	No prediabetes
36	Improvement in diet habits independent of physical activity helps to reduce incident diabetes among prediabetic Asian Indian men"	Ram	2014	No mobile-application based intervention
37	App engagement and weight loss in a mobile health study (MDIAB)	Ranjani	2019	Not peer-reviewed
38	Acceptability of Two Novel Mhealth Applications for Diabetes Prevention in Urban and Rural India	Ranjani	2022	Not peer-reviewed
39	Effect of a Culturally Adapted Behavioral Intervention for Latino Adults on Weight Loss over 2 Years: A Randomized Clinical Trial	Rosas	2020	No prediabetes
40	mHealth Intervention to Improve Cardiometabolic Health in Rural Hispanic Adults: A Pilot Study	Rowland	2022	No prediabetes
41	Adaptation of a health game for adults over 50 and examination of its acceptability, feasibility and preliminary impact when implemented with a nutrition education intervention"	Ruggiero	2023	No prediabetes
42	Precision Recruitment and Engagement of Individuals at Risk for Diabetes and Hypertension in Clinical Trials (PREDHICT): A Randomized Trial for an E-Persuasive Mobile Application to Inform Decision Making about Clinical Trials	Seixas	2023	No prediabetes
43	Efficacy of IVRS-based mHealth intervention in reducing cardiovascular risk in metabolic syndrome: A cluster randomized trial	Sharma	2021	No prediabetes
44	The effectiveness of an mHealth intervention on diabetes risk factors and body composition	Simkó	2023	No prediabetes
45	A smartphone-based exercise adherence intervention for people with metabolic syndrome: A feasibility pilot study	Sit	2016	Not peer-reviewed
46	Reducing Sedentary Time and Whole-Body Insulin Sensitivity in Metabolic Syndrome: A 6-Month Randomized Controlled Trial	Sjöros	2023	No prediabetes
47	The effect of extended personalization to a combined lifestyle intervention program	Smid	2023	Not peer-reviewed
48	Health supported exercise intervention in patients with metabolic syndrome: A randomized clinical trial	Stuckey	2013	Full-text not found
49	Does a prescriptive exercise program with mobile health tracking improve cardio-metabolic risk factors to a greater extent than exercise prescription alone?	Stuckey	2013	Not peer-reviewed
50	Does prescriptive exercise with mobile health tracking improve self-efficacy and health status in individuals with metabolic syndrome?	Stuckey	2013	Not peer-reviewed
51	Mediating Effects of the 'eCoFit' Physical Activity Intervention for Adults at Risk of or Diagnosed with Type 2 Diabetes"	Wilczynska	2019	No prediabetes
52	The effects of the eCoFit RCT on depression and anxiety symptoms among adults with or at risk of Type 2 Diabetes	Wilczynska	2022	No prediabetes[2]
53	Research on the application of health management model based on the perspective of mobile health	Yang	2019	No prediabetes

[1] Studies were screened by the following order and screening stopped with the first criterion not met: No duplicate, Peer-reviewed (incl. conference abstracts and gray literature), RCT study design, Populations at risk of developing T2DM, Mobile application-based intervention, English

[2] These studies included prediabetic populations amongst other criteria (e.g. overweight, diabetic populations). Given that no results were reported separately for the prediabetic populations only, we excluded those borderline studies.

Appendix S8: Cohen´s kappa interrater-reliability coefficients

Title and abstract screening

		NA	
		Include	Exclude
LS	Include	29	13
	Exclude	65	5498

P_0	0.986
$P_{Include}$	0.000
$P_{Exclude}$	0.976
P_e	0.976
k	0.420

Total studies N=5,605
Total conflicts N=78

Full-text screening

		NA	
		Include	Exclude
LS	Include	15*	5

	Exclude	7**	42

P_0	0.826
$P_{Include}$	0.092
$P_{Exclude}$	0.484
P_e	0.576
k	0.590

Total studies N=69
Total conflicts N=12

* N=3 studies with the inclusion criterion of Metabolic Syndrome were included by NA and excluded by LS. The authors agreed to exclude those studies to keep the population as homogenous as possible. Although Metabolic Syndrome contains one prediabetes criterion it cannot be stated that all participants in the study were prediabetic and therefore the studies were excluded.
** N=3 studies were first included by NA and LS and later excluded as they were then identified as related additional publications using data from already included RCTs.

Formulas:

		NA	
		Include	Exclude
LS	Include	a	b
	Exclude	c	d

P_0 (observed proportionate agreement) = $\frac{a+d}{a+b+c+d}$

$P_{Include}$ (expected probability of inclusion) = $\frac{a+b}{a+b+c+d} \times \frac{a+c}{a+b+c+d}$

$P_{Exclude}$ (expected probability of exclusion) = $\frac{c+d}{a+b+c+d} \times \frac{b+d}{a+b+c+d}$

P_e (random agreement probability) = $P_{Include} + P_{Exclude}$

k (inter-rater reliability) = $\frac{P0-Pe}{1-Pe}$

Appendix S9: Risk of bias assessment

First author (year)	D1	D2	D3	D4	D5	Overall
Bender (2018)	+	+	+	+	!	+
Ben Yacov (2021)	+	+	+	+	!	!
Block (2015)	+	+	+	+	!	+
Chen (2020)	!	!	-	+	!	-
Chung (2023)	+	+	+	!	+	!
Fukuoka (2015)	!	+	+	!	!	!
Griauzde (2019)	+	!	!	+	+	!
Karvela (2024)	!	+	+	!	!	!
Katula (2022)	+	+	+	+	!	!
Kitazawa (2023)	!	+	!	!	!	!
Lakka (2023)	+	+	!	!	+	!
Luo (2022)	!	+	!	!	+	!
McLeod (2020)	+	+	+	+	+	+
Staite (2020)	+	!	+	+	+	+
Toro-Ramos (2020)	+	+	+	!	+	!
Whelan (2019)	!	!	+	!	!	!
Lim (2022)	+	+	+	!	!	!

+ Low risk
! Some concerns
- High risk

D1 Randomisation process
D2 Deviations from the intended interventions
D3 Missing outcome data
D4 Measurement of the outcome
D5 Selection of the reported result

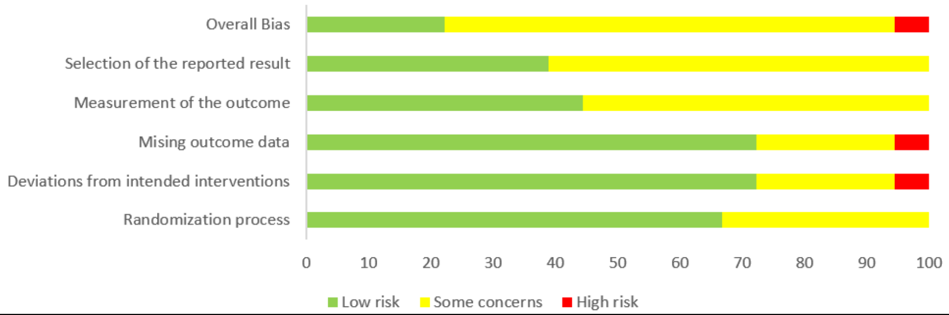

Appendix S10: Details of effect directions analysis of non-meta-analyzed outcomes

Primary author, year	Outcome Category	Outcome	Instrument used, unit	Follow-up in months	Type of Analysis	Effect Estimate	CI / P-Value	Effect direction (summary and interpretation +/-) †	Same/different effect direction
Ben-Yacov, 2021	Lifestyle/ behaviour	Diet, energy intake	App log in, kcal/day	6	Difference between groups after 6 m	-28.4	(-114.9, 58.0) / P=0.516	Less energy intake in PPT-diet group (intervention) than in MED-diet group (control)<br>+	N/A
		Diet, carbohydrate intake	App log in, g/day	6	Difference between groups after 6 m	-93.2	(-101.9, -84.4) / P=0.0*	Less carbohydrate intake in PPT-diet group (intervention) than in MED-diet group (control)<br>N/A	N/A
		Diet, carbohydrate intake proportion	App log in, % of energy	6	Difference between groups after 6 m	-22	(-23.2, -20.9) / P=0.0*	Less carbohydrate proportion intake in PPT-diet group (intervention) than in MED-diet group (control)<br>N/A	N/A
		Diet, protein intake	App log in, g/day	6	Difference between groups after 6 m	5.1	(-0.5, 10.8), P=0.074	More protein intake in PPT-diet group (intervention) than in MED-diet group (control)<br>N/A	N/A
		Diet, protein intake proportion	App log in, % of energy	6	Difference between groups after 6 m	1.7	(0.7, 2.6) / P=0.001*	More protein proportion intake in PPT-diet group (intervention) than in MED-diet group (control)<br>N/A	N/A
		Diet, total fat intake	App log in, g/day	6	Difference between groups after 6 m	37.1	(32.1, 42.1) / P=0.0*	More total fat intake in PPT-diet group (intervention) than in MED-diet group (control)<br>N/A	N/A
		Diet, total fat intake proportion	App log in, % of energy	6	Difference between groups after 6 m	21	(19.8, 22.2) / P=0.0*	More total fat proportion intake in PPT-diet group (intervention) than in MED-diet group (control)<br>N/A	N/A
		Diet, saturated fat intake	App log in, g/day	6	Difference between groups after 6 m	11.5	(9.7, 13.2) / P=0.0*	More saturated fat intake in PPT-diet group (intervention) than in MED-diet group (control)<br>N/A	N/A

Primary author, year	Outcome Category	Outcome	Instrument used, unit	Follow-up in months	Type of Analysis	Effect Estimate	CI / P-Value	Effect direction (summary and interpretation +/-) †	Same/different effect direction
		Diet, saturated fat intake proportion	App log in, % of energy	6	Difference between groups after 6 m	6.4	(5.8, 7.0) / P=0.0*	More saturated fat proportion intake in PPT-diet group (intervention) than in MED-diet group (control)<br>N/A	N/A
		Diet, fiber	App log in, g/day	6	Difference between groups after 6 m	-10.8	(-12.9, -8.7) / P=0.0*	Less fiber intake in PPT-diet group (intervention) than in MED-diet group (control)<br>N/A	N/A
		Diet, fiber proportion	App log in, g/1000 kcal	6	Difference between groups after 6 m	-6.2	(-7.2, -5.2) / P=0.0*	Less fiber proportion intake in PPT-diet group (intervention) than in MED-diet group (control)<br>N/A	N/A
		Physical activity	App log in, h/week	6	Within group difference after 6 m / P-value of between-group difference	MED: 1.11 ± 1.70<br>PPT: 1.15 ± 1.67	N/A / P=0.51	slight improvement in PPT group more than MED group<br>+	N/A
	Clinical	Postprandial (postmeal) glucose responses (PPGRs) 5 hours	mg/dL*h	6	DiD	N/A	(-12.26, -7.56) / P< 0.001^	Improved in the PPT group more than in the MED group<br>+	N/A
		Fatty liver index	N/A	6	DiD	N/A	(-8.34, -1.41) / P< 0.01^	Improved in the PPT group more than in the MED group<br>+	N/A
		AST	µkat/L	6	DiD	N/A	(-0.06, 0) / P=n.s	Improved in the PPT group more than in the MED group<br>+	N/A
		ALT	µkat/L	6	DiD	N/A	(-0.09, 0.02) / P=n.s	Improved in the PPT group more than in the MED group<br>+	N/A
		US Liver	N/A	6	DiD	N/A	(-0.18, 0.25) / P=n.s	Decreased less in the PPT group more than in the MED group<br>-	N/A
Block, 2015	Clinical	5% Weight loss	%	6	Descriptive statistics after 6 m	• 35.3% in intervention group<br>• 8.3% in control	P<0.001 ^	Improved in the intervention group more than in the control group<br>+	Both increased
		Achieving a normal fasting glucose	%	6	Descriptive statistics after 6 m	• 40.5% in intervention group<br>• 17.7% in control	P<0.001 ^	Improved in the intervention group more than in the control group<br>+	Both increased
		BMI reduction by ≥1 kg/m^2	%	6	Descriptive statistics after 6 m	• 44.9% in intervention group<br>• 18.6% in control	P<0.001 ^	Improved in the intervention group more than in the control	Both increased

Primary author, year	Outcome Category	Outcome	Instrument used, unit	Follow-up in months	Type of Analysis	Effect Estimate	CI / P-Value	Effect direction (summary and interpretation +/-) †	Same/different effect direction
								group<br>+	
		Framingham 8-year diabetes risk score	Framingham 8-year diabetes risk score	6	Within group difference after 6 m (calculated)	• Intervention: -5%<br>• Control: -1.41%	P<0.001 ^	Improved in the intervention group more than in the control group<br>+	Both decreased
Chen, 2020	Health Competence	Knowledge of health behavior/ ability to select health information	JNCSA Applied Behavioral Analysis for Health Promotion (JABH), score (higher scores mean better function)	3	DiD	6.9	(-1.2, 15.0) / P=0.096	Improved in the intervention group more than in the control group<br>+	Intervention increased / Control decreased
	Lifestyle/behaviour	Dietary composition/nutrient balance	JNCSA Applied Behavioral Analysis for Health Promotion (JABH), score (higher scores mean better function)	3	DiD	3.9	(-2.6, 10.4) / P=0.234	Improved in the intervention group more than in the control group<br>+	Both increased
		Eating behavior	JNCSA Applied Behavioral Analysis for Health Promotion (JABH), score (higher scores mean better function)	3	DiD	8	(1.8, 14.3) / P=0.013^	Improved in the intervention group more than in the control group<br>+	Both increased
		Sweets and alcoholic drinks	JNCSA Applied Behavioral Analysis for Health Promotion (JABH), score (higher scores mean better function)	3	DiD	1.9	(-3.3, 7.0) / P=0.474	Improved in the intervention group more than in the control group<br>+	Intervention increased / Control decreased
		Dietary assessment score	JNCSA Applied Behavioral Analysis for Health Promotion (JABH), score (higher scores mean better function)	3	DiD	22	(16.8, 27.2) / P<0.001^	Improved in the intervention group more than in the control group<br>+	Intervention increased / Control decreased
		Physical activity	JNCSA Applied Behavioral Analysis for Health Promotion (JABH), score (higher scores mean better function)	3	DiD	12	(4.8, 19.1) / P=0.001^	Improved in the intervention group more than in the control group<br>+	Intervention increased / Control decreased
		Activity volition	JNCSA Applied Behavioral Analysis for Health Promotion (JABH), score (higher scores mean better function)	3	DiD	2	(-6.1, 10.0) / P=0.626	Improved in the intervention group more than in the control group<br>+	Both increased
	Quality of life	QoL: Stress	JNCSA Applied Behavioral Analysis for Health Promotion (JABH), score	3	DiD	-2.5	(-9.1, 4.1) / P=0.455	Worsened in the intervention group more than in the control group<br>-	Both increased

Primary author, year	Outcome Category	Outcome	Instrument used, unit	Follow-up in months	Type of Analysis	Effect Estimate	CI / P-Value	Effect direction (summary and interpretation +/-) †	Same/different effect direction
			(higher scores mean better function)						
		QoL: Fatigue/sleep quality	JNCSA Applied Behavioral Analysis for Health Promotion (JABH), score (higher scores mean better function)	3	DiD	5.1	(-2.7, 12.9) / P=0.199	Improved in the intervention group more than in the control group<br>+	Both increased
		Comprehensive Assessment	Sum of components of JNCSA Applied Behavioral Analysis for Health Promotion (JABH), score (higher scores mean better function)	3	DiD	4.7	(1.1, 8.2) /<br>P= 0.012^	Improved in the intervention group more than in the control group<br>+	Both increased
	Clinical	Skeletal muscle mass	kg	3	DiD	0.1	(- 0.2, 0.5) / P=0.437	Less decrease in the intervention group than the control group<br>+	Both decreased
		Body fat mass	kg	3	DiD	-1.6	(- 2.4, - 0.8) / P<0.001^	Improved in the intervention group more than in the control group<br>+	Both decreased
		Percentage body fat	%	3	DiD	-1.7	(- 2.6, - 0.7) / P=0.001^	Improved in the intervention group more than in the control group<br>+	Both decreased
		Visceral fat area	cm2	3	DiD	-9.1	(- 13.9, - 4.4) /<br>P<0.001^	Improved in the intervention group more than in the control group<br>+	Both decreased
		Time in postprandial hyperglycemia	h/day	3	DiD	−0.81	(-1.56, -0.06) /<br>P=0.035^	Improved in the intervention group more than in the control group<br>+	Both decreased
		Frequencies of postprandial hyperglycemia	n/day	3	DiD	-0.1	(- 0.22, 0.03) / P=0.035	Improved in the intervention group more than in the control group<br>+	Both decreased
Chung, 2023	Lifestyle/behaviour	Dietary behaviour	Dietary approaches to stop hypertension (DASH), score (higher scores mean better correspondence to DASH diet)	3	• OMG * T2 (vs. CG * T1)<br>• TCMG * T2 (vs. CG * T1)	• **mhealth vs control: 0.22**<br>• TCM vs control: 1.21	• **mhealth vs. control: (−1.42, 1.86) / P=0.79**<br>• TCM vs control: (−0.62, 3.06) / P=0.19	Improved in the intervention groups (mhealth and TCM) more than in the control group<br>+	N/A

Primary author, year	Outcome Category	Outcome	Instrument used, unit	Follow-up in months	Type of Analysis	Effect Estimate	CI / P-Value	Effect direction (summary and interpretation +/-) †	Same/different effect direction
		Dietary behaviour	Dietary approaches to stop hypertension (DASH), score (higher scores mean better correspondence to DASH diet)	4	*OMG * T3 (vs. CG * T1)<br>*TCMG * T3 (vs. CG * T1)	• **mhealth vs. control: -0.51**<br>• TCM vs control: 0.99	• **mhealth vs. control: (-2.49, 1.48) / P=0.62**<br>• TCM vs control: (-1.05, 3.04) / P=0.34	**Worsened in the intervention mhealth group more than in the control group**<br>**-**<br><br>improved in the intervention TCM group more than in the control group<br>+	N/A
		Physical activity	IPAQ (Taiwanese version), metabolic equivalents (MET)-minutes/week	3	• OMG * T2 (vs. CG * T1)<br>• TCMG * T2 (vs. CG * T1)	• **mhealth vs control: 236.33**<br>• TCM vs control: 248.59	• **mhealth vs. control: (−231.76 to 704.43) / P=0.32**<br>• TCM vs control: (−191.18, 688.37) / P=0.27	Improved in the intervention groups (mhealth and TCM) more than in the control group<br>+	N/A
		Physical activity	IPAQ (Taiwanese version), metabolic equivalents (MET)-minutes/week	4	• OMG * T3 (vs. CG * T1)<br>• TCMG * T3 (vs. CG * T1)	• **mhealth vs. control: 213.18**<br>• TCM vs control: 122.74	• **mhealth vs. control: (-360.50, 786.86) / P=0.47**<br>• TCM vs control: (-459.22, 704.71) / P=0.68	Improved in the intervention groups (mhealth and TCM) more than in the control group<br>+	N/A
		Meredian body energy	Meridian Energy Analysis Device (MEAD), µA (MEAD values for the 24 acupoints (Ryodoraku points) along the 12 meridians ranging from 0 to 200 µA. Individuals with prediabetes have a lower level of body energy)	3	• OMG * T2 (vs. CG * T1)<br>• TCMG * T2 (vs. CG * T1)	• **mhealth vs control: –0.16**<br>• TCM vs control: 8.60	• **mhealth vs. control: (−11.23 to 10.91) / P=0.98**<br>• TCM vs control: (−1.91 to 19.11) / P=0.11	**Worsened in the intervention mhealth group more than in the control group**<br>**-**<br><br>Improved in the intervention TCM group more than in the control group<br>+	N/A
		Meredian body energy	Meridian Energy Analysis Device (MEAD), µA (MEAD values for the 24 acupoints (Ryodoraku points) along the 12 meridians ranging from 0 to 200 µA. Individuals with prediabetes have a lower level of body energy)	4	• OMG * T3 (vs. CG * T1)<br>• TCMG * T3 (vs. CG * T1)	• **mhealth vs. control: -5.84**<br>• TCM vs control: 7.81	• **mhealth vs. control: (-17.68, 6.01) / P=0.33**<br>• TCM vs control: (-3.36, 18.98) / P=0.17	**worsened in the intervention mhealth group more than in the control group**<br>**-**<br><br>improved in the intervention TCM group more than in the control group<br>+	N/A
		QoL: Physical component	SF-36 (Taiwanese version), score (higher scores indicate a better physical aspect of HRQOL)	3	• OMG * T2 (vs. CG * T1)<br>• TCMG * T2 (vs. CG * T1)	• **mhealth vs control: 2.56**<br>• TCM vs control: 4.93	• **mhealth vs. control: (−0.44, 5.56) / P=0.10**<br>• TCM vs control: (1.97, 7.89) / P=0.001^	Improved in the intervention groups (mhealth and TCM) more than in the control group<br>+	N/A

Primary author, year	Outcome Category	Outcome	Instrument used, unit	Follow-up in months	Type of Analysis	Effect Estimate	CI / P-Value	Effect direction (summary and interpretation +/-) †	Same/different effect direction
		QoL: Physical component	SF-36 (Taiwanese version), score (higher scores indicate a better physical aspect of HRQOL)	4	• OMG * T3 (vs. CG * T1)<br>• TCMG * T3 (vs. CG * T1)	• **mhealth vs. control: 2.90**<br>• TCM vs control: 4.89	• **mhealth vs. control: (-0.07, 5.89) / P=0.06**<br>• TCM vs control: (1.92, 7.87) / P=0.001	Improved in the intervention groups (mhealth and TCM) more than in the control group<br>+	N/A
		QoL: Mental component	SF-36 (Taiwanese version), score (higher scores indicate a better mental aspect of HRQOL)	3	• OMG * T2 (vs. CG * T1)<br>• TCMG * T2 (vs. CG * T1)	• **mhealth vs control: 4.63**<br>• TCM vs control: 8.11	• **mhealth vs. control: (0.91, 8.35) / P=0.02^**<br>• TCM vs control: (3.82, 12.40) / P<0.001^	Improved in the intervention groups (mhealth and TCM) more than in the control group<br>+	N/A
		QoL: Mental component	SF-36 (Taiwanese version), score (higher scores indicate a better mental aspect of HRQOL)	4	• OMG * T3 (vs. CG * T1)<br>• TCMG * T3 (vs. CG * T1)	• **mhealth vs. control: 2.68**<br>• TCM vs control: 7.26	• **mhealth vs. control: (-1.16, 6.51) / P=0.17**<br>• TCM vs control: (3.35, 11.17) / P<0.001	Improved in the intervention groups (mhealth and TCM) more than in the control group<br>+	N/A
		Yang-deficiency	Score of Body composition questionnaire (BCQ), higher scores indicating a greater deficiency	3	• OMG * T2 (vs. CG * T1)<br>• TCMG * T2 (vs. CG * T1)	• **mhealth vs control: -0.81**<br>• TCM vs control: −3.15	• **mhealth vs. control: (−3.16, 1.53) / P=0.50**<br>• TCM vs control: (−6.09, −0.21) / P=0.04^	Improved in the intervention groups (mhealth and TCM) more than in the control group<br>+	N/A
		Yang-deficiency	Score of Body composition questionnaire (BCQ), higher scores indicating a greater deficiency	4	• OMG * T3 (vs. CG * T1)<br>• TCMG * T3 (vs. CG * T1)	• **mhealth vs. control: −0.46**<br>• TCM vs control: −2.37	• **mhealth vs. control: (−2.73, 1.81) / P=0.69**<br>• TCM vs control: (−5.04, 0.29) / P=0.08	Improved in the intervention groups (mhealth and TCM) more than in the control group<br>+	N/A
		Yin-deficiency	Score of Body composition questionnaire (BCQ), higher scores indicating a greater deficiency	3	• OMG * T2 (vs. CG * T1)<br>• TCMG * T2 (vs. CG * T1)	• **mhealth vs control: -0.02**<br>• TCM vs control: -2.29	• **mhealth vs. control: (-2.56, 2.52) / P=0.99**<br>• TCM vs control: (-5.28, 0.70) / P=0.13	Improved in the intervention groups (mhealth and TCM) more than in the control group<br>+	N/A
		Yin-deficiency	Score of Body composition questionnaire (BCQ), higher scores indicating a greater deficiency	4	• OMG * T3 (vs. CG * T1)<br>• TCMG * T3 (vs. CG * T1)	• **mhealth vs. control: 0.03**<br>• TCM vs control: -1.62	• **mhealth vs. control: (-2.08, 2.13) / P=0.98**<br>• TCM vs control: (-4.21, 0.96) / P=0.22)	**worsened in the intervention mhealth group more than in the control group**<br>**-**<br>improved in the intervention TCM group more than in the control group<br>+	N/A
		Phlegm-stasis	Score of Body composition questionnaire (BCQ), higher scores indicating a greater deficiency	3	• OMG * T2 (vs. CG * T1)<br>• TCMG * T2 (vs. CG * T1)	• **mhealth vs control: −1.36**<br>• TCM vs control: −3.45	• **mhealth vs. control: (−4.16, 1.44) / P=0.34**<br>• TCM vs control: (−6.49, −0.42) / P=0.03^	Improved in the intervention groups (mhealth and TCM) more than in the control group<br>+	N/A
		Phlegm-stasis	Score of Body composition questionnaire (BCQ), higher scores indicating a greater deficiency	4	• OMG * T3 (vs. CG * T1)<br>• TCMG * T3 (vs. CG * T1)	• **mhealth vs control: −1.88**<br>• TCM vs control: −3.30	• **mhealth vs. control: (−4.38, 0.63) / P=0.14**<br>• TCM vs control: (−6.01, −0.58) / P=0.02^	Improved in the intervention groups (mhealth and TCM) more than in the control group<br>+	N/A

Primary author, year	Outcome Category	Outcome	Instrument used, unit	Follow-up in months	Type of Analysis	Effect Estimate	CI / P-Value	Effect direction (summary and interpretation +/-) †	Same/different effect direction
Fukuoka, 2015	Lifestyle/behaviour	Physical activity	7-Day Physical Activity Recall (PAR), kcal/kg/day	3	Within group difference after 3 m (calculated)	• Control: -0.8<br>• Intervention: 1	N/A / P=0.06 (between group after 3 m) / P=0.03 (DiD)^	Improved in the intervention group more than the control group<br>+	Increased in intervention / Decreased in control
		Physical activity	7-Day Physical Activity Recall (PAR), kcal/kg/day	5	Within group difference after 5 m (calculated)	• Control: 0.2<br>• Intervention: 0.5	N/A / P=0.98 (between group after 5 m) / P=0.03 (DiD)^	Improved in the intervention group more than the control group<br>+	Both increased
		Physical activity	Pedometer, steps/day	3	Within group difference after 3 m	• Control: −125±3,451<br>• Intervention: 2,888±4,998	N/A / P<0.001 (between group after 3 m) / P<0.001 (DiD)^	Improved in the intervention group more than the control group<br>+	Increased in intervention / Decreased in control
		Physical activity	Pedometer, steps/day	5	Within group difference after 5 m	• Control: -734±3,308<br>• Intervention: 2,551±4,712	N/A / P<0.001 (between group after 5 m) / P<0.001 (DiD)^	Improved in the intervention group more than the control group<br>+	Increased in intervention / Decreased in control
		Physical activity	Pedometer, steps/hour	3	Within group difference after 3 m	• Control: 9±251<br>• Intervention: 151±321	N/A / P=0.004 (between group after 3 m) / P<0.001 (DiD)^	Improved in the intervention group more than the control group<br>+	Both increased
		Physical activity	Pedometer, steps/hour	5	Within group difference after 5 m	• Control: −21±240<br>• Intervention: 141±327	N/A / P=0.001 (between group after 5 m) / P<0.001 (DiD)^	Improved in the intervention group more than the control group<br>+	Increased in intervention / Decreased in control
		Physical activity	IPAQ, MET min/day	3	Within group difference after 3 m	Light PA (1–2 METs):<br>• Control: −29±141<br>• Intervention: 41±147	N/A / P=0.01* (between group after 3 m) / P=0.06 (DiD)	Improved in the intervention group more than the control group<br>+	Increased in intervention / Decreased in control
		Physical activity	IPAQ, MET min/day	5	Within group difference after 5 m	Light PA (1-2 METs):<br>• Control: −49±151<br>• Intervention: 17±167	N/A / P=0.05* (between group after 5 m) / P=0.06 (DiD)	Improved in the intervention group more than the control group<br>+	Increased in intervention / Decreased in control
		Physical activity	IPAQ, MET min/day	3	Within group difference after 3 m	Moderate PA (3–5 METs):<br>• Control: −1.7±30<br>• Intervention: 21±46	N/A / P<0.001* (between group after 3 m) / P<0.001^ (DiD)	Improved in the intervention group more than the control group<br>+	Increased in intervention / Decreased in control
		Physical activity	IPAQ, MET min/day	5	Within group difference after 5 m	Moderate PA (3-5 METs):<br>• Control: −4.2±29<br>• Intervention: 16±46	N/A / P<0.001* (between group after 5 m) / P<0.001^ (DiD)	Improved in the intervention group more than the control group<br>+	Increased in intervention / Decreased in control
		Physical activity	IPAQ, MET min/day	3	Within group difference after 3 m	Vigorous PA (6–8 METs):<br>• Control: −0.35±4.6<br>• Intervention: 0.91±6.3	N/A / P=0.14 (between group after 3 m) / P=0.13 (DiD)	Improved in the intervention group more than the control group<br>+	Increased in intervention / Decreased in control

Primary author, year	Outcome Category	Outcome	Instrument used, unit	Follow-up in months	Type of Analysis	Effect Estimate	CI / P-Value	Effect direction (summary and interpretation +/-) †	Same/different effect direction
		Physical activity	IPAQ, MET min/day	5	Within group difference after 5 m	Vigorous PA (6-8 METs):<br>• Control: −0.36±5.1<br>• Intervention: 2.30±11.0	N/A / P=0.01* (between group after 5 m) / P=0.13 (DiD)	Improved in the intervention group more than the control group<br>+	Increased in intervention / Decreased in control
		Diet, caloric intake	Block Food Frequency Questionnaire, kcal/day	5	Within group difference after 5 m (calculated)	• Control: -320<br>• Intervention: -430	N/A / P=0.19 (between group after 5 m) / P=0.19 (DiD)	Improved in the intervention group more than the control group<br>+	Both decreased
		Diet, fat intake	Block Food Frequency Questionnaire, g/day	5	Within group difference after 5 m (calculated)	• Control: -14.4<br>• Intervention: -20.9	N/A / P=0.14 (between group after 5 m) / P=0.18 (DiD)	Less fat intake in the intervention group than the control group<br>N/A	Both decreased
		Diet, saturated fat intake	Block Food Frequency Questionnaire, g/day	5	Within group difference after 5 m (calculated)	• Control: -4.3<br>• Intervention: -9.2	N/A / P=0.007* (between group after 5 m) / P=0.007^ (DiD)	Less fat intake in the intervention group than the control group<br>N/A	Both decreased
		Diet, sugar-sweetened beverages	Block Food Frequency Questionnaire, kcal/day	5	Within group difference after 5 m (calculated)	• Control: -8.5<br>• Intervention: -24.5	N/A / P=0.02* (between group after 5 m) / P=0.02^ (DiD)	Improved in the intervention group more than the control group<br>+	Both decreased
	Clinical	Depression	Center for Epidemiologic Studies Depression Scale (CES-D), score (higher scores indicating the presence of more symptomatology)	5	Within group difference after 5 m (calculated)	• Control: 3.9<br>• Intervention: 1.5	N/A / P=0.27 (between group after 5 m) / P=0.41 (DiD)	Less increase in the intervention group than the control group<br>+	Both increased
	Motivation and Self-Efficacy	Self-efficacy (barriers to being active, and social support)	Self-Efficacy for Physical Activity Survey, score (higher scores indicate better self-efficacy)	3	Within group difference after 3 m (calculated)	• Control: -0.9<br>• Intervention: -1.2	N/A / P=0.79 (between group after 5 m) / P=0.35 (DiD)	Worsened in the intervention group more than in the control group<br>-	Both decreased
		Self-efficacy (barriers to being active, and social support)	Self-Efficacy for Physical Activity Survey, score (higher scores indicate better self-efficacy)	5	Within group difference after 5 m (calculated)	• Control: -0.8<br>• Intervention: 0.2	N/A / P=0.22 (between group after 5 m) / P=0.35 (DiD)	Improved in the intervention group more than in the control group<br>+	Intervention increased / Control decreased
		Self-efficacy (barriers to being active, and social support)	Barriers to Being Active Quiz, score (score of 5 or above in any category shows that this is an important barrier)	3	Within group difference after 3 m (calculated)	Lack of time:<br>• Control: -0.11<br>• Intervention: -0.99	N/A / P=0.08 (between group after 3 m) / P=0.02^ (DiD)	Improved in the intervention group more than in the control group<br>+	Both decreased
		Self-efficacy (barriers to being active, and social support)	Barriers to Being Active Quiz, score (score of 5 or above in any category shows that this is an important barrier)	5	Within group difference after 5 m (calculated)	Lack of time:<br>• Control: 0.35<br>• Intervention: -1.12	N/A / P=0.03* (between group after 5 m) / P=0.02^ (DiD)	Improved in the intervention group more than in the control group<br>+	Intervention decreased / Control increased

Primary author, year	Outcome Category	Outcome	Instrument used, unit	Follow-up in months	Type of Analysis	Effect Estimate	CI / P-Value	Effect direction (summary and interpretation +/-) †	Same/different effect direction
		Self-efficacy (barriers to being active, and social support)	Barriers to Being Active Quiz, score (score of 5 or above in any category shows that this is an important barrier)	3	Within group difference after 3 m (calculated)	Social influence:<br>• Control: 0.54<br>• Intervention: -0.75	N/A / P=0.005* (between group after 3 m) / P=0.01^ (DiD)	Improved in the intervention group more than in the control group<br>+	Intervention decreased / Control increased
		Self-efficacy (barriers to being active, and social support)	Barriers to Being Active Quiz, score (score of 5 or above in any category shows that this is an important barrier)	5	Within group difference after 5 m (calculated)	Social influence:<br>• Control: 0.1<br>• Intervention: -0.72	N/A / P=0.03* (between group after 5 m) / P=0.01^ (DiD)	Improved in the intervention group more than in the control group<br>+	Intervention decreased / Control increased
		Self-efficacy (barriers to being active, and social support)	Barriers to Being Active Quiz, score (score of 5 or above in any category shows that this is an important barrier)	3	Within group difference after 3 m (calculated)	Lack of energy:<br>• Control: -0.09<br>• Intervention: -1.08	N/A / P=0.02* (between group after 3 m) / P=0.04^ (DiD)	Improved in the intervention group more than in the control group<br>+	Both decreased
		Self-efficacy (barriers to being active, and social support)	Barriers to Being Active Quiz, score (score of 5 or above in any category shows that this is an important barrier)	5	Within group difference after 5 m (calculated)	Lack of energy:<br>• Control: 0.08<br>• Intervention: -1.07	N/A / P=0.05* (between group after 5 m) / P=0.04^ (DiD)	Improved in the intervention group more than in the control group<br>+	Intervention decreased / Control increased
		Self-efficacy (barriers to being active, and social support)	Barriers to Being Active Quiz, score (score of 5 or above in any category shows that this is an important barrier)	3	Within group difference after 3 m (calculated)	Lack of willpower:<br>• Control: -0.93<br>• Intervention: -2.81	N/A / P<0.001* (between group after 3 m) / P<0.001^ (DiD)	Improved in the intervention group more than in the control group<br>+	Both decreased
		Self-efficacy (barriers to being active, and social support)	Barriers to Being Active Quiz, score (score of 5 or above in any category shows that this is an important barrier)	5	Within group difference after 5 m (calculated)	Lack of willpower:<br>• Control: -0.51<br>• Intervention: -2.91	N/A / P<0.001* (between group after 5 m) / P<0.001^ (DiD)	Improved in the intervention group more than in the control group<br>+	Both decreased
		Self-efficacy (barriers to being active, and social support)	Barriers to Being Active Quiz, score (score of 5 or above in any category shows that this is an important barrier)	3	Within group difference after 3 m (calculated)	Fear of injury:<br>• Control: 0.23<br>• Intervention: -0.47	N/A / P=0.20 (between group after 3 m) / P=0.23 (DiD)	Improved in the intervention group more than in the control group<br>+	Intervention decreased / Control increased
		Self-efficacy (barriers to being active, and social support)	Barriers to Being Active Quiz, score (score of 5 or above in any category shows that this is an important barrier)	5	Within group difference after 5 m (calculated)	Fear of injury:<br>• Control: 0.55<br>• Intervention: -0.31	N/A / P=0.19 (between group after 5 m) / P=0.23 (DiD)	Improved in the intervention group more than in the control group<br>+	Intervention decreased / Control increased
		Self-efficacy (barriers to being active, and social support)	Barriers to Being Active Quiz, score (score of 5 or above in any category shows that this is an important barrier)	3	Within group difference after 3 m (calculated)	Lack of skill:<br>• Control: 0.54<br>• Intervention: -1.25	N/A / P=0.06 (between group after 3 m) / P=0.01^ (DiD)	Improved in the intervention group more than in the control group<br>+	Intervention decreased / Control increased

Primary author, year	Outcome Category	Outcome	Instrument used, unit	Follow-up in months	Type of Analysis	Effect Estimate	CI / P-Value	Effect direction (summary and interpretation +/-) †	Same/different effect direction
		Self-efficacy (barriers to being active, and social support)	Barriers to Being Active Quiz, score (score of 5 or above in any category shows that this is an important barrier)	5	Within group difference after 5 m (calculated)	Lack of skill:<br>• Control: 0.27<br>• Intervention: -0.79	N/A / P=0.31 (between group after 5 m) / P=0.01^ (DiD)	Improved in the intervention group more than in the control group<br>+	Intervention decreased / Control increased
		Self-efficacy (barriers to being active, and social support)	Barriers to Being Active Quiz, score (score of 5 or above in any category shows that this is an important barrier)	3	Within group difference after 3 m (calculated)	Lack of resources:<br>• Control: 0.47<br>• Intervention: -0.77	N/A / P=0.16 (between group after 3 m) / P=0.10 (DiD)	Improved in the intervention group more than in the control group<br>+	Intervention decreased / Control increased
		Self-efficacy (barriers to being active, and social support)	Barriers to Being Active Quiz, score (score of 5 or above in any category shows that this is an important barrier)	5	Within group difference after 5 m (calculated)	Lack of resources:<br>• Control: 0.28<br>• Intervention: -0.23	N/A / P=0.77 (between group after 5 m) / P=0.10 (DiD)	Improved in the intervention group more than in the control group<br>+	Intervention decreased / Control increased
		Self-efficacy (barriers to being active, and social support)	Social support for physical activity, score (higher scores indicate better support)	3	Within group difference after 3 m (calculated)	Family:<br>• Control: 0.1<br>• Intervention: 6.3	N/A / P=0.87 (between group after 3 m) / P=0.13 (DiD)	Improved in the intervention group more than in the control group<br>+	Both increased
		Self-efficacy (barriers to being active, and social support)	Social support for physical activity, score (higher scores indicate better support)	5	Within group difference after 5 m (calculated)	Family:<br>• Control: -0.2<br>• Intervention: 2.8	N/A / P=0.50 (between group after 5 m) / P=0.13 (DiD)	Improved in the intervention group more than in the control group<br>+	Intervention increased / Control decreased
		Self-efficacy (barriers to being active, and social support)	Social support for physical activity, score (higher scores indicate better support)	3	Within group difference after 3 m (calculated)	Friend:<br>• Control: -0.4<br>• Intervention: 0.7	N/A / P=0.71 (between group after 3 m) / P=0.13 (DiD)	Improved in the intervention group more than in the control group<br>+	Intervention increased / Control decreased
		Self-efficacy (barriers to being active, and social support)	Social support for physical activity, score (higher scores indicate better support)	5	Within group difference after 5 m (calculated)	Friend:<br>• Control: -3.1<br>• Intervention: 0.3	N/A / P=0.17 (between group after 5 m) / P=0.13 (DiD)	Improved in the intervention group and worsened in the control group<br>+	Intervention increased / Control decreased
	Clinical	Hip circumference	cm	3	Within group difference after 3 m (calculated)	• Control: -1.1<br>• Intervention: -3.5	N/A / P=0.04* (between group after 5 m) / P<0.001^ (DiD)	Improved in the intervention group more than in the control group<br>+	Both decreased
		Hip circumference	cm	5	Within group difference after 5 m (calculated)	• Control: -1.2<br>• Intervention: -5.3	N/A / P=0.008* (between group after 5 m) / P<0.001^ (DiD)	Improved in the intervention group more than in the control group<br>+	Both decreased
Griauzde, 2019	Motivation and Self-Efficacy	Motivation (Autonomous motivation to prevent T2DM)	Treatment Self-Regulation Questionnaire (TSRQ), score (higher scores indicate greater levels)	3	DiD	• App-plus vs control: 0.08<br>• **App-only vs control: 0.22**	• App-plus vs control: N/A / P=0.77<br>• **App-only vs control: N/A / P=0.51**	Improved in the intervention group more than in the control group<br>+	N/A

Primary author, year	Outcome Category	Outcome	Instrument used, unit	Follow-up in months	Type of Analysis	Effect Estimate	CI / P-Value	Effect direction (summary and interpretation +/-) †	Same/different effect direction
Karvela, 2024	Lifestyle/behaviour	Diet, energy intake	FFQ, N/A	6.5	DiD	• Intervention vs control: -0.01 (0.01)<br>• **Exploratory vs control: 0 (0.01)**	• Intervention vs control: P = 0.444<br>• **Exploratory vs control: P = 0.97**	No difference between exploratory and control groups --> null effect	N/A
		Diet, salt intake	FFQ, N/A	6.5	DiD	• Intervention vs control: 0 (0.01)<br>• **Exploratory vs control: 0.01 (0.01)**	• Intervention vs control: P = 0.77<br>• **Exploratory vs control: P = 0.39**	Worsened in the exploratory group more than in the control group<br>-	N/A
		Diet, carbohydrate intake	FFQ, N/A	6.5	DiD	• Intervention vs control: -0.03 (0.01)<br>• **Exploratory vs control: -0.03 (0.01)**	• Intervention vs control: P = 0.04^<br>• **Exploratory vs control: P = 0.01^**	Slight decrease in carbohydrate intake in the exploratory group than in the control group<br>N/A	N/A
		Diet, fat intake	FFQ, N/A	6.5	DiD	• Intervention vs control: 0 (0.01)<br>• **Exploratory vs control: 0.01 (0.01)**	• Intervention vs control: P = 0.74<br>• **Exploratory vs control: P = 0.55**	Slight increase in fat intake in the exploratory group than in the control group --> worse outcome in exploratory group<br>N/A	N/A
		Diet, saturated fat intake	FFQ, N/A	6.5	DiD	• Intervention vs control: 0 (0.01)<br>• **Exploratory vs control: 0.01 (0.01)**	• Intervention vs control: P = 0.97<br>• **Exploratory vs control: P = 0.53**	Slight increase in saturated fat intake in the exploratory group than in the control group --> worse outcome in exploratory group<br>N/A	N/A
	Clinical	Fat mass	kg	1.5	DiD	• Intervention vs control: − 1.12 (0.59)<br>• **Exploratory vs control: 1.01 (0.62)**	• Intervention vs control: P = 0.06<br>• **Exploratory vs control: P = 0.11**	Worsened in the exploratory group more than in the control group<br>-	N/A
		Fat mass	kg	3	DiD	• Intervention vs control: − 1.91 (0.76)<br>• **Exploratory vs control: 1.39 (0.75)**	• Intervention vs control: P = 0.01<br>• **Exploratory vs control: P = 0.07**	Worsened in the exploratory group more than in the control group<br>-	N/A
		Fat mass	kg	6.5	DiD	• Intervention vs control: − 1.01 (1.05)<br>• **Exploratory vs control: 1.79 (1.05)**	• Intervention vs control: P = 0.34<br>• **Exploratory vs control: P = 0.09**	Worsened in the exploratory group more than in the control group<br>-	N/A
		HOMA-B	N/A	1.5	DiD	• Intervention vs control: − 25.24 (32.33)<br>• **Exploratory vs control: 17.74 (334.45)**	• Intervention vs control: P = 0.38<br>• **Exploratory vs control: P = 0.51**	Worsened in the exploratory group more than in the control group<br>-	N/A

Primary author, year	Outcome Category	Outcome	Instrument used, unit	Follow-up in months	Type of Analysis	Effect Estimate	CI / P-Value	Effect direction (summary and interpretation +/-) †	Same/different effect direction
		HOMA-B	N/A	3	DiD	• Intervention vs control: − 1.55 (35.33)<br>• **Exploratory vs control: − 14.35 (35.15)**	• Intervention vs control: P = 0.94<br>• **Exploratory vs control: P = 0.72**	Improved in the exploratory group more than in the control group<br>+	N/A
		HOMA-B	N/A	6.5	DiD	• Intervention vs control: 19.7 (20.43)<br>• **Exploratory vs control: 30.62 (30.9)**	• Intervention vs control: P = 0.17<br>• **Exploratory vs control: P = 0.16**	Worsened in the exploratory group more than in the control group<br>-	N/A
Katula, 2021	Clinical	% in normal HbA1c (<5.7%) range	%	4	Descriptive statistics after 4 m	46.56% in intervention group vs 3.6% in control	N/A / P<0.025^	Improved in the intervention group more than in the control group<br>+	N/A
		% in normal HbA1c (<5.7%) range	%	12	Descriptive statistics after 12 m	57.61% in intervention group vs 47.92% in control	N/A	Improved in the intervention group more than in the control group<br>+	N/A
		5% Weight loss	%	4	Descriptive statistics after 4 m	46.54% in intervention group vs 12.3% in control	N/A / P<0.025^	Improved in the intervention group more than in the control group<br>+	N/A
		5% Weight loss	%	12	Descriptive statistics after 12 m	43.35% in intervention group vs 20.96% in control	N/A / P<0.025^	Improved in the intervention group more than in the control group<br>+	N/A
Kitazawa, 2023	Lifestyle/behaviour	Diet, protein intake	N/A, g/day	3	Within group difference after 3 m	• Control: -0.2<br>• Intervention: -1.5	N/A / P=0.746	Higher decrease in protein intake in the intervention group than in the control group<br>N/A	Both decreased
		Diet, fat intake	N/A, g/day	3	Within group difference after 3 m	• Control: -0.6<br>• Intervention: -2.4	N/A / P=0.866	Higher decrease in fat intake in the intervention group than in the control group<br>N/A	Both decreased
		Diet, carbohydrate intake	N/A, g/day	3	Within group difference after 3 m	• Control: -4.4<br>• Intervention: -22.7	N/A / P=0.049^	Higher decrease in carbohydrate intake in the intervention group than in the control group<br>N/A	Both decreased

Primary author, year	Outcome Category	Outcome	Instrument used, unit	Follow-up in months	Type of Analysis	Effect Estimate	CI / P-Value	Effect direction (summary and interpretation +/-) †	Same/different effect direction
		Diet, energy intake	Brief Diet History Questionnaire, kcal/day	3	Within group difference after 3 m	• Control: -60.58<br>• Intervention: -176.38	N/A / P=0.156	Higher decrease in energy intake in the intervention group than in the control group<br>+	Both decreased
	Clinical	Weight reduction of ≥2 kg	%	3	Within group difference after 3 m	• Control: 15.9%<br>• Intervention: 32.8%	N/A / P=0.28	Higher in the intervention group than in the control group<br>+	Both increased
		Time in range (TIR) (70-140 mg/dL)	min/day	3	Within group difference after 3 m	• Control: -2.6<br>• Intervention: 31.5	N/A / P=0.03^	Improved in the intervention group more than in the control group<br>+	Control decreased / Intervention increased
		Time below range (TBR) (<70 mg/dL)	min/day	3	Within group difference after 3 m	• Control: 23.5<br>• Intervention: -8.9	N/A / P=0.032^	Improved in the intervention group more than in the control group<br>+	Control increased / Intervention decreased
		Continuous overlapping net glycemic action (CONGA) at 2 hours	N/A	3	Within group difference after 3 m	• Control: −2.5<br>• Intervention: −1.6	N/A / P=0.307	Improved in the control group more than in the intervention group<br>-	Both decreased
		Mean amplitude of glucose excursion (MAGE)	N/A	3	Within group difference after 3 m	• Control: −1.8<br>• Intervention: −3.4	N/A / P=0.755	Improved in the intervention group more than in the control group<br>+	Both decreased
Lakka, 2023	Lifestyle/behaviour	Diet	Healthy diet index, score (total score ranges from 0 (lowest quality) to 100 (highest quality)	12	DiD	• **DIGI vs control: 0.40**<br>• DIGI+GROUP vs control: 1.63	• **DIGI vs control: (-0.29, 1.09)**<br>• DIGI+GROUP vs control: (0.95, 2.31)	Improved in the intervention groups more than in the control group<br>+	N/A
		Physical activity	Self-reported, h/wk	12	DiD	• **DIGI vs control: 0.48**<br>• DIGI+GROUP vs control: 0.64	• **DIGI vs control: (-0.25, 1.20) / N/A**<br>• DIGI+GROUP vs control: (-0.08, 1.37) / N/A	Improved in the intervention groups more than in the control group<br>+	N/A
		Physical activity	Sedentary time, h/d	12	DiD	• **DIGI vs control: 0.04**<br>• DIGI+GROUP vs control: -0.04	• **DIGI vs control: (-0.11, 0.18) / N/A**<br>• DIGI+GROUP vs control: (-0.18, 0.10) / N/A	**Improved in the DIGI+GROUP more than in the control group**<br>**+**<br>Worsened in the DIGI group more than in the control group<br>-	N/A
Lim, 2022	Lifestyle/behaviour	Diet, caloric intake	nBuddy dashboard's nutrient analysis platform, kcal/day	3	DiD	−326.5	(−458.1, −194.9) / P<0.001^	Improved in the intervention group more than in the control group<br>+	Both decreased

Primary author, year	Outcome Category	Outcome	Instrument used, unit	Follow-up in months	Type of Analysis	Effect Estimate	CI / P-Value	Effect direction (summary and interpretation +/-) †	Same/different effect direction
		Diet, caloric intake	nBuddy dashboard's nutrient analysis platform, kcal/day	6	DiD	−397.1	(−530.4, −263.8) / P<0.001^	Improved in the intervention group more than in the control group<br>+	Both decreased
		Diet, carbohydrate intake	nBuddy dashboard's nutrient analysis platform, g/day	3	DiD	−42.2	(−58.9, −25.6) / P<0.001^	Higher decrease in carbohydrate intake in the intervention group than in the control group<br>N/A	Both decreased
		Diet, carbohydrate intake	nBuddy dashboard's nutrient analysis platform, g/day	6	DiD	−53.5	(−70.1, −36.8) / P<0.001^	Higher decrease in carbohydrate intake in the intervention group than in the control group<br>N/A	Both decreased
		Diet, sugar intake	nBuddy dashboard's nutrient analysis platform, g/day	3	DiD	−16.7	(−24.2, −9.3) / P<0.001^	Improved in the intervention group more than in the control group<br>+	Both decreased
		Diet, sugar intake	nBuddy dashboard's nutrient analysis platform, g/day	6	DiD	−19.0	(−26.7, −11.3) / P<0.001^	Improved in the intervention group more than in the control group<br>+	Both decreased
		Diet, protein intake	nBuddy dashboard's nutrient analysis platform, g/day	3	DiD	−5.1	(−11.7, 1.5) / P=0.132	Higher decrease in protein intake in the intervention group than in the control group<br>N/A	Both decreased
		Diet, protein intake	nBuddy dashboard's nutrient analysis platform, g/day	6	DiD	−10.8	(−18.7, −2.9) / P=0.007^	Higher decrease in protein intake in the intervention group than in the control group<br>N/A	Both decreased
		Diet, total fat intake	nBuddy dashboard's nutrient analysis platform, g/day	3	DiD	−15.4	(−22.5, −8.4) / P<0.001^	Higher decrease in fat intake in the intervention group than in the control group<br>N/A	Both decreased
		Diet, total fat intake	nBuddy dashboard's nutrient analysis platform, g/day	6	DiD	−15.4	(−22.8, −8.0) / P<0.001^	Higher decrease in fat intake in the intervention group than in the control group<br>N/A	Both decreased
		Diet, saturated fat intake	nBuddy dashboard's nutrient analysis platform, g/day	3	DiD	−7.6	(−10.7, −4.4) / P<0.001^	Higher decrease in fat saturated intake in the intervention group than in the	Both decreased

Primary author, year	Outcome Category	Outcome	Instrument used, unit	Follow-up in months	Type of Analysis	Effect Estimate	CI / P-Value	Effect direction (summary and interpretation +/-) †	Same/different effect direction
								control group<br>N/A	
		Diet, saturated fat intake	nBuddy dashboard's nutrient analysis platform, g/day	6	DiD	-7.1	(−10.3, −3.9) / P<0.001^	Higher decrease in fat saturated intake in the intervention group than in the control group<br>N/A	Both decreased
		Diet, fiber intake	nBuddy dashboard's nutrient analysis platform, g/day	3	DiD	−0·5	(−2.2, 1.1) / P=0.526	Higher decrease in fiber intake in the intervention group than in the control group<br>N/A	Both decreased
		Diet, fiber intake	nBuddy dashboard's nutrient analysis platform, g/day	6	DiD	−0·8	(−2.7, 1.1) / P=0.398	Higher decrease in fiber intake in the intervention group than in the control group<br>N/A	Both decreased
		Physical activity	Self-reported, min/week or h/wk	3	DiD	42.5	(6.9, 78.1) / P=0.019	Improved in the intervention group more than in the control group<br>+	Both increased
		Physical activity	Self-reported, min/week or h/wk	6	DiD	33.1	(-4.5, 70-7) / P=0.084	Improved in the intervention group more than in the control group<br>+	Both increased
	Clinical	Creatinine	umol/L	3	DiD	1.8	(−0.7, 4.3) / P=0.149	Improved in the intervention group more than in the control group<br>+	Control decreased / Intervention increased
		Creatinine	umol/L	6	DiD	3.0	(0.6, 5.3) / P=0.015^	Improved in the intervention group more than in the control group<br>+	Control decreased / Intervention increased
Luo, 2022	Clinical	Abdominal fat	cm2	3	Within group change after 3 m	• MD: −56.6 (−68.2 to −44.9)<br>• TJD: −45.8 (−56.4 to −35.2)<br>• CD: −47.1 (−57.4 to −36.7)	P=0.17 (DiD)	Improved in the MD group more than in the control group<br>+	All decreased
		Abdominal fat	cm2	6	Within group change after 6 m	• MD: -65.6 (−77.5 to −53.6)<br>• TJD: −53.9 (−65.3 to −42.5)<br>• CD: −65.2 (−76.0 to −54.5)	P=0.17 (DiD)	Improved in the MD group more than in the control group<br>+	All decreased

Primary author, year	Outcome Category	Outcome	Instrument used, unit	Follow-up in months	Type of Analysis	Effect Estimate	CI / P-Value	Effect direction (summary and interpretation +/-) †	Same/different effect direction
		Visceral adipose tissue.	cm2	3	Within group change after 3 m	• MD: −34.1 (−41.6 to −26.50)<br>• TJD: −30.8 (−37.7 to −23.9)<br>• CD: −29.0 (−35.7 to −22.3)	P=0.44 (DiD)	Improved in the MD group more than in the control group<br>+	All decreased
		Visceral adipose tissue.	cm2	6	Within group change after 6 m	• MD: −39.88 (−47.7 to −32.1)<br>• TJD: −36.2 (−43.7 to −28.6)<br>• CD: −40.5 (−47.5 to −33.5)	P=0.44 (DiD)	Improved in the CD group more than in the MD<br>-	All decreased
		Subcutaneous adipose tissue	cm2	3	Within group change after 3 m	• MD: −22.4 (−28.2 to −16.6)<br>• TJD: −15.2 (−20.6 to −9.90)<br>• CD: −17.7 (−22.9 to −12.5)	P=0.26 (DiD)	Improved in the MD group more than in the control group<br>+	All decreased
		Subcutaneous adipose tissue	cm2	6	Within group change after 6 m	• MD: −25.8 (−31.8 to −19.7)<br>• TJD: −17.7 (−23.6 to −11.9)<br>• CD: −24.4 (−29.8 to −19.0)	P=0.26 (DiD)	Improved in the MD group more than in the control group<br>+	All decreased
		Area under the curve (AUC) glucose	OGTT, mmol*h/L	3	Within group change after 3 m	• MD: 0.23 (−0.29 to 0.75)<br>• TJD: 0.86 (0.37-1.35)<br>• CD: 0.66 (0.17-1.15)	P=0.96 (DiD)	Increased less in the MD group than in the CD group<br>+	All decreased
		Area under the curve (AUC) glucose	OGTT, mmol*h/L	6	Within group change after 6 m	• MD: −0.93 (−1.46 to −0.40)<br>• TJD: −0.19 (−0.73 to 0.34)<br>• CD: −0.47 (−0.99 to 0.05)	P=0.96 (DiD)	Improved in the MD group more than in the control group<br>+	All decreased
		AUC insulin	OGTT, uIU*h/mL	3	Within group change after 3 m	• MD: −15.0 (−34.0 to 3.95)<br>• TJD: −31.7 (−49.9 to −13.4)<br>• CD: −30.5 (−48.5 to −12.6)	P=0.33 (DiD)	Improved in the MD group less than in the control group<br>-	All decreased

Primary author, year	Outcome Category	Outcome	Instrument used, unit	Follow-up in months	Type of Analysis	Effect Estimate	CI / P-Value	Effect direction (summary and interpretation +/-) †	Same/different effect direction
		AUC insulin	OGTT, uIU*h/mL	6	Within group change after 6 m	• MD: −28.0 (−47.4 to −8.64)<br>• TJD: −27.7 (−47.5 to −8.00)<br>• CD: −26.0 (−45.0 to −7.09)	P=0.33 (DiD)	Improved in the MD group more than in the control group<br>+	All decreased
		Matsuda Index	OGTT, N/A	3	Within group change after 3 m	• MD: 3.62 (2.02-5.22)<br>• TJD: 2.76 (1.23-4.30)<br>• CD: 2.29 (0.78-3.81)	P=0.74 (DiD)	Improved in the MD group more than in the control group<br>+	All increased
		Matsuda Index	OGTT, N/A	6	Within group change after 6 m	• MD: 4.84 (3.19-6.50)<br>• TJD: 3.62 (1.94-5.31)<br>• CD: 4.06 (2.46-5.66)	P=0.74 (DiD)	Improved in the MD group more than in the control group<br>+	All increased
		SD of interstitial glucose from CGM	CGM, mmol/L	3	Within group change after 3 m	N/A	MD vs CD: P= 0.43<br>P group x time = 0.035	Decreased more in the MD group than in the CD group<br>+	Both decreased
		SD of interstitial glucose from CGM	CGM, mmol/L	6	Within group change after 6 m	N/A	MD vs CD: P= 0.43<br>P group x time = 0.035	Decreased less in the MD group than in the CD group<br>-	Both decreased
		sqrt(Time above range (TAR)) (>10 mmol/L)	CGM. %	3	Within group change after 3 m	N/A	MD vs CD: P= 0.12<br>P group x time = 0.036	Decreased more in the MD group than in the CD group<br>+	Both decreased
		sqrt(Time above range (TAR)) (>10 mmol/L)	CGM. %	6	Within group change after 6 m	N/A	MD vs CD: P= 0.12<br>P group x time = 0.036	Decreased less in the MD group than in the CD group<br>-	Both decreased
		sqrt(Time below range (TBR)) (<3.9 mmol/L)	CGM. %	3	Within group change after 3 m	N/A	MD vs CD: P= 0.024<br>P groupxtime = 0.015	Increased more in the MD group than in the CD group<br>-	Both increased
		sqrt(Time below range (TBR)) (<3.9 mmol/L)	CGM. %	6	Within group change after 6 m	N/A	MD vs CD: P= 0.024^<br>P groupxtime = 0.015	Increased less in the MD group than in the CD group<br>+	Both increased
Staite, 2020	Lifestyle/behaviour	Physical activity	Wristband, mean steps/day	6	DiD	−382.90	(−860.65, 94.85) / N/A	Improved in the control group more than the intervention group<br>-	Both decreased
		Physical activity	Wristband, mean steps/day	12	DiD	-92.64	(−380.92, 566.20) / N/A	Improved in the control group more than the intervention group<br>-	Both decreased

Primary author, year	Outcome Category	Outcome	Instrument used, unit	Follow-up in months	Type of Analysis	Effect Estimate	CI / P-Value	Effect direction (summary and interpretation +/-) †	Same/different effect direction
		Physical activity	IPAQ, sitting, minutes	6	Descriptive summary at 6 m (calculated increase/decrease)	• control: median: 300, IQR: 210.0 (-60)<br>• intervention: median: 360, IQR: 300.0 (-30)	N/A / N/A	Sitting: Improved in the control group more than the intervention group<br>-	Both decreased
		Physical activity	IPAQ, sitting, minutes	12	Descriptive summary at 12 m (calculated increase/decrease)	• control: median: 300, IQR: 300.0 (-60)<br>• intervention: median: 360, IQR: 300.0 (-30)	N/A / N/A	Improved in the control group more than the intervention group<br>-	Both decreased
		Physical activity	IPAQ, total activity, metabolic equivalent of tasks (MET) min/week	6	Descriptive summary at 6 m (calculated increase/decrease)	• control: median: 2373, IQR: 3540 (307)<br>• intervention: median: 2026, IQR: 3648 (573)	N/A / N/A	Total: Improved in the intervention group more than the control group<br>+	Both increased
		Physical activity	IPAQ, total activity, metabolic equivalent of tasks (MET) min/week	12	Descriptive summary at 12 m (calculated increase/decrease)	• control: median: 2496, IQR: 2758.5 (430)<br>• intervention: median: 1782, IQR: 3132.0 (329)	N/A / N/A	Improved in the control group more than the intervention group<br>-	Both increased
	Clinical	Waist to hip ratio	N/A	12	DiD	0.00	(−0.02 to 0.02) /N/A	Null effect	Both decreased
Whelan, 2019	Lifestyle/ behaviour	Physical activity	Step count/active minutes/number of flights of stairs/reductions in the number of reminders to move, Min/day	1.5	N/A	There was no clear trend toward increased physical activity over the 6 weeks	N/A / N/A	N/A	N/A
Xu, 2020	Lifestyle/ behaviour	Diet, energy intake	FFQ25, kcal/day	3	• Change within group after 3 m (calculated)<br>• GLMM Intervention vs control<br>• GLMM difference in mean change	***Median (IQR)***<br>• Control: -864<br>• Intervention: -575<br><br>***GLMM e^beta***<br>• 1.02<br>• 0.9	N/A / P=0.56<br><br>GLMM<br>• (0.86-1.21) / P=1<br>• (0.78-1.07) / P=0.53	Decreased in the control group more than the intervention group (in comparison with baseline)<br>-	Both decreased
		Diet, energy intake	FFQ25, kcal/day	6	• Change within group after 6 m (calculated)	***Median (IQR)***<br>• Control: -173<br>• Intervention: -1014<br><br>***GLMM e^beta***	N/A / P<0.001*<br><br>GLMM<br>• (0.62-0.88) / P<0.001	Decreased in the intervention group more than the control group (in comparison with baseline)<br>+	Both decreased

Primary author, year	Outcome Category	Outcome	Instrument used, unit	Follow-up in months	Type of Analysis	Effect Estimate	CI / P-Value	Effect direction (summary and interpretation +/-) †	Same/different effect direction
					• GLMM Intervention vs control<br>• GLMM difference in mean change	• 0.74<br>• 0.66	• (0.56-0.77) / P<0.001		
		Diet, carbohydrate intake	FFQ25, g/day	3	• Change within group after 3 m (calculated)<br>• GLMM Intervention vs control<br>• GLMM difference in mean change	***Median (IQR)***<br>• Control: -40.5<br>• Intervention: -40.5<br><br>***GLMM e^beta***<br>• 1.08<br>• 1.05	N/A / P=0.3<br><br>GLMM<br>• (0.86-1.35) / P=0.90<br>• (0.84-1.31) / P=0.98	Null effect	Both decreased
		Diet, carbohydrate intake	FFQ25, g/day	6	• Change within group after 6 m (calculated)<br>• GLMM Intervention vs control<br>• GLMM difference in mean change	***Median (IQR)***<br>• Control: -33.8<br>• Intervention: -68.9<br><br>***GLMM e^beta***<br>• 0.85<br>• 0.83	N/A / P=0.08<br><br>GLMM<br>• (0.68-1.06) / P=0.26<br>• (0.66-1.03) / P<0.001	Higher decrease in carbohydrate intake in the intervention group than the control group<br>N/A	Both decreased
		Diet, carbohydrate intake proportion	FFQ25, %	3	• Change within group after 3 m (calculated)<br>• GLMM Intervention vs control<br>• GLMM difference in mean change	***Mean (SD)***<br>• Control: 0.8<br>• Intervention: 0.3<br><br>***GLMM e^beta***<br>• 1.02<br>• 0.99	N/A / P=0.47<br><br>GLMM<br>• (0.92-1.14) / P=0.49<br>• (0.86-1.13) / P=1.00	Higher increase in carbohydrate intake proportion in the control group than the intervention group<br>N/A	Both increased
		Diet, carbohydrate intake proportion	FFQ25, %	6	• Change within group after 6 m (calculated)<br>• GLMM Intervention vs control<br>• GLMM difference in mean change	***Mean (SD)***<br>• Control: 0.5<br>• Intervention: 3.9<br><br>***GLMM e^beta***<br>• 1.12<br>• 1.07	N/A / P<0.001*<br><br>GLMM<br>• (1.00-1.25) / P=0.03<br>• (0.94-1.23) / P=0.60	Higher increase in carbohydrate intake proportion in the intervention group than the control group<br>N/A	Both increased

Primary author, year	Outcome Category	Outcome	Instrument used, unit	Follow-up in months	Type of Analysis	Effect Estimate	CI / P-Value	Effect direction (summary and interpretation +/-) †	Same/different effect direction
		Diet, protein intake	FFQ25, g/day	3	• Change within group after 3 m (calculated)<br>• GLMM Intervention vs control<br>• GLMM difference in mean change	***Median (IQR)***<br>• Control: -14.7<br>• Intervention: -12.5<br><br>***GLMM e^beta***<br>• 1.03<br>• 1.17	N/A / P=0.95<br><br>GLMM<br>• (0.83-1.27) / P=1.00<br>• (0.91-1.49) / P=0.40	Higher decrease in protein intake in the control group than the intervention group<br>N/A	Both decreased
		Diet, protein intake	FFQ25, g/day	6	• Change within group after 6 m (calculated)<br>• GLMM Intervention vs control<br>• GLMM difference in mean change	***Median (IQR)***<br>• Control: -10.4<br>• Intervention: -38.6<br><br>***GLMM e^beta***<br>• 0.75<br>• 0.85	N/A / P<0.001*<br><br>GLMM<br>• (0.61-0.93) / P=0.003<br>• (0.67-1.09) / P=0.38	Higher decrease in protein intake in the intervention group than in the control group<br>N/A	Both decreased
		Diet, protein intake proportion	FFQ25, %	3	• Descriptive summary at 3 m<br>• GLMM Intervention vs control<br>• GLMM difference in mean change	• **Control: after 3 m:** 20.0 (18.6-23.3) Median (IQR) / **Baseline**: 20.8 (2.3) Mean (SD)<br>• **Intervention: after 3 m:** 19.8(18.0-21.5) Median (IQR) / **Baseline**: 20.1 (2.4) Mean (SD)<br><br>***GLMM e^beta***<br>• 0.97<br>• 1.00	N/A / P=0.3<br><br>GLMM<br>• (0.89-1.06) / P=0.92<br>• (0.90-1.11) / P=1.00	Higher decrease in protein intake proportion in the control group than the intervention group<br>N/A	Both decreased
		Diet, protein intake proportion	FFQ25, %	6	• Descriptive summary at 6 m<br>• GLMM Intervention vs control<br>• GLMM difference in mean change	• **Control: after 6 m:** 20.11 (18.3-22.59) Median (IQR) / **Baseline**: 20.8 (2.3) Mean (SD)<br>• **Intervention: after 6:** 18.6(17.5-20.8) Median (IQR) / **Baseline**: 20.1 (2.4) Mean (SD)<br><br>***GLMM e^beta***<br>• 0.95	N/A / P=0.07<br><br>GLMM<br>• (0.87-1.04) / P=0.52<br>• (0.88-1.09) / P=1.00	Higher decrease in protein intake proportion in the intervention group than in the control group<br>N/A	Both decreased

Primary author, year	Outcome Category	Outcome	Instrument used, unit	Follow-up in months	Type of Analysis	Effect Estimate	CI / P-Value	Effect direction (summary and interpretation +/-) †	Same/different effect direction
						• 0.98			
		Diet, fat intake	FFQ25, g/day	3	• Change within group after 3 m (calculated)<br>• GLMM Intervention vs control<br>• GLMM difference in mean change	***Median (IQR)***<br>• Control: -15.9<br>• Intervention: -16.35<br><br>***GLMM e^beta***<br>• 1.00<br>• 1.01	N/A / P=0.71<br><br>GLMM<br>• (0.79-1.28) / P=1.00<br>• (0.76-1.35) / P=1.00	Higher decrease in fat intake in the intervention group than in the control group<br>N/A	Both decreased
		Diet, fat intake	FFQ25, g/day	6	• Change within group after 6 m (calculated)<br>• GLMM Intervention vs control<br>• GLMM difference in mean change	***Median (IQR)***<br>• Control: -1<br>• Intervention: -37.9<br><br>***GLMM e^beta***<br>• 0.70<br>• 0.71	N/A / P<0.001*<br><br>GLMM<br>• (0.55-0.90) / P<0.001<br>• (0.54-0.95) / P<0.001	Higher decrease in fat intake in the intervention group than in the control group<br>N/A	Both decreased
		Diet, fat intake proportion	FFQ25, %	3	• Descriptive summary at 3 m<br>• GLMM Intervention vs control<br>• GLMM difference in mean change	• **Control: after 3 m:** 35.9 (7.5) Mean (SD) / **Baseline**: 37.3 (33.0-40.9) Median (IQR)<br>• **Intervention: after 3 m:** 35.2 (5.5) Mean (SD) / **Baseline**: 36.8 (30.9-38.3) Median (IQR)<br><br>**GLMM e^beta**<br>• 0.99<br>• 1.01	N/A / P=0.63<br><br>GLMM<br>• (0.88-1.10) / P=1.00<br>• (0.88-1.16) / P=1.00	Higher decrease in fat intake proportion in the intervention group than in the control group<br>N/A	Both decreased
		Diet, fat intake proportion	FFQ25, %	6	• Descriptive summary at 6 m<br>• GLMM Intervention vs control<br>• GLMM difference in mean change	• **Control: after 6 m:** 36.5 (5.6) Mean (SD) / **Baseline**: 37.3 (33.0-40.9) Median (IQR)<br>• **Intervention: after 6 m**: 32.2 (6.3) Mean (SD) / **Baseline**: 36.8 (30.9-38.3) Median (IQR)	N/A / P<0.001*<br><br>GLMM<br>• (0.79-0.99) / P<0.001<br>• (0.79-1.05) / P=0.33	Higher decrease in fat intake proportion in the intervention group than in the control group<br>N/A	Both decreased

Primary author, year	Outcome Category	Outcome	Instrument used, unit	Follow-up in months	Type of Analysis	Effect Estimate	CI / P-Value	Effect direction (summary and interpretation +/-) †	Same/different effect direction
						GLMM e^beta<br>• 0.89<br>• 0.91			
		Physical activity	IPAQ (Chinese version), metabolic equivalent of tasks (MET) min/week	3	• Change within group after 3 m (calculated)<br>• GLMM Intervention vs control<br>• GLMM difference in mean change	**TOTAL, *Median (IQR)***<br>• Control: 436.5<br>• Intervention: 1054.5<br><br>**TOTAL, *GLMM e^beta***<br>• 1.08<br>• 1.39	N/A / P=0.80<br><br>GLMM<br>• (0.68-1.73) / P=0.99<br>• (0.96-2.00) / P=0.10	Improved in the intervention group more than the control group<br>+	Both increased
		Physical activity	IPAQ (Chinese version), metabolic equivalent of tasks (MET) min/week	6	• Change within group after 6 m (calculated)<br>• GLMM Intervention vs control<br>• GLMM difference in mean change	**TOTAL, *Median (IQR)***<br>• Control: 487.5<br>• Intervention: 1840.5<br><br>**TOTAL, *GLMM e^beta***<br>• 1.07<br>• 1.38	N/A / P=0.38<br><br>GLMM<br>• (0.67-1.71) / P=1.00<br>• (0.96-1.99) / P=0.11	Improved in the intervention group more than the control group<br>+	Both increased

Primary author, year	Outcome Category	Outcome	Instrument used, unit	Follow-up in months	Type of Analysis	Effect Estimate	CI / P-Value	Effect direction (summary and interpretation +/-) †	Same/different effect direction
	Motivation and Self-Efficacy	Stage of dietary behaviors change	Stage of change (SOC) scale, n (%)	3	• Change within group after 3 m (calculated)<br>• GLMM Intervention vs control<br>• GLMM difference in mean change	Precontemplation:<br>• Control: -5(-12.5%)<br>• Intervention: -6(-16.6%)<br>Contemplation:<br>• Control: -3(-7.5%)<br>• Intervention: -14(38.9%)<br>Preparation:<br>• Control: 5(12.5)<br>• Intervention: 6(38.8%)<br>Action:<br>• Control: 2(5%)<br>• Intervention: 5(13.8%)<br>Maintenance:<br>• Control: 1(2.5%)<br>• Intervention: 1(2.8%)<br>----<br>GLMM e^beta<br>• 4.69<br>• 4.06	N/A / P=0.02*<br><br>GLMM<br>• (0.71-31.04) / P=0.42<br>• (0.95-17.32) / P=0.26	Less individuals in "Precontemplation" and "Contemplation", and more individuals in "Preparation" in the intervention than in the control group<br>+	N/A

Primary author, year	Outcome Category	Outcome	Instrument used, unit	Follow-up in months	Type of Analysis	Effect Estimate	CI / P-Value	Effect direction (summary and interpretation +/-) †	Same/different effect direction
		Stage of dietary behaviors change	Stage of change (SOC) scale, n (%)	6	• Change within group after 6 m (calculated)<br>• GLMM Intervention vs control<br>• GLMM difference in mean change	Precontemplation:<br>• Control: -9(-22.5%)<br>• Intervention: -7(-19.4%)<br>Contemplation:<br>• Control: -4(-10%)<br>• Intervention: -20(-55.6%)<br>Preparation:<br>• Control: 2(5%)<br>• Intervention: 1(2.7%)<br>Action:<br>• Control: 10(25%)<br>• Intervention: 19(52.8%)<br>Maintenance:<br>• Control: 1(2.5%)<br>• Intervention: 7(4.5%)<br>----<br>GLMM e^beta<br>• 26.80<br>• 26.80	N/A / P<0.001<br><br>GLMM<br>• (3.51-204.91) / P=0.002<br>• (3.51-204.91) / P=0.002	More individuals in "Action" and "Maintenance" in the intervention than in the control group<br>+	N/A

Primary author, year	Outcome Category	Outcome	Instrument used, unit	Follow-up in months	Type of Analysis	Effect Estimate	CI / P-Value	Effect direction (summary and interpretation +/-) †	Same/different effect direction
		Stage of physical activity change	Stage of change (SOC) scale, n (%)	3	• Change within group after 3 m (calculated)<br>• GLMM Intervention vs control<br>• GLMM difference in mean change	Precontemplation:<br>• Control: 0(0%)<br>• Intervention: -4(-11.2%)<br>Contemplation:<br>• Control: -11(-27.5%)<br>• Intervention: -9(-25%)<br>Preparation:<br>• Control: 8(20.5%) (13(32.5) - 5 (12.5))<br>• Intervention: 5(16.8%) (11(30.6) - 6 (16.7))<br>Action:<br>• Control: 3(7.5%) (7(17.5) - 4 (10.0))<br>• Intervention: 5(14%) (10(27.8) - 5 (13.8))<br>Maintenance:<br>• Control: 0(0%) 2(5.0) - 2 (5.0)<br>• Intervention: 3(8.3%) (3(8.3) - 0(0.0))<br>----<br>GLMM e^beta<br>• 3.24<br>• 4.49	N/A / P=0.10<br><br>GLMM<br>• (0.55-18.99) / P=0.66<br>• (0.92-21.87) / P=0.29	More individuals in "Action" in the intervention than in the control group<br>+	N/A

Primary author, year	Outcome Category	Outcome	Instrument used, unit	Follow-up in months	Type of Analysis	Effect Estimate	CI / P-Value	Effect direction (summary and interpretation +/-) †	Same/different effect direction
		Stage of physical activity change	Stage of change (SOC) scale, n (%)	6	• Change within group after 6 m (calculated)<br>• GLMM Intervention vs control<br>• GLMM difference in mean change	Precontemplation:<br>• Control: -6(-15%)<br>• Intervention: -6(-16.7%)<br>Contemplation:<br>• Control: -8(20%)<br>• Intervention: -18(-50%)<br>Preparation:<br>• Control: 6(15%) (11(27.5) - 5 (12.5))<br>• Intervention: 2(5.5%) (8(22.2) - 6 (16.7))<br>Action:<br>• Control: 5(12.5%) (9(22.5) - 4 (10.0))<br>• Intervention: 13(36.2%) (18(50.0) - 5 (13.8))<br>Maintenance:<br>• Control: 3(7%) (5(12.5) - 2 (5.0))<br>• Intervention: 9(25%) (9(25.0) - 0(0.0))<br>---<br>GLMM e^beta<br>• 15.60<br>• 21.65	N/A / P<0.001*<br><br>GLMM<br>• (2.67-91.04) / P=0.01<br>• (3.91-119.89) / P=0.003	More individuals in "Action" and "Maintenance" and less individuals in "Precontemplation, Contemplation, and Preparation" in the intervention than in the control group by the end of the intervention<br>+	N/A
	Clinical	BMI	N/A	6	• GLMM (included age, gender, education level, occupational classification as covariates)	GLMM:<br>e^beta: 0,99	GLMM:<br>(0,93; -1,05) / P=1,00	Significant greater probability of BMI decrease in Intervention group<br>+	N/A
		Waist circumference	N/A	6	• GLMM (included age, gender, education level, occupational classification as covariates)	GLMM:<br>e^beta: 1,02	GLMM:<br>(0,97;-1,07) / P=0,75	Significant greater probability of Waist circumference decrease in Intervention group<br>+	N/A

† Signs with + or – indicated if the intervention had better or worse effects respectively in the intervention in comparison with the control group. Whenever there were more than 1 intervention groups in the study, the main intervention group of interest was written in **bold**.

Abbreviations: CI: confidence interval; DiD: difference between means from baseline to follow-up; GLMM: generalized linear mixed models; N/A: not available.

Appendix S11: Effect directions of clinical outcomes of longest follow-up

First author, year †	Outcome							
	Anthropometry		Blood Glucose	Blood Lipids	Blood pressure	Other		
	[1]Weight / [2]BMI / [3]Achieving 5% Weight Loss / [4]Achieving ≥2 kg weight loss / [5]BMI reduction likelihood / [6]BMI reduction by ≥1 kg/m2	[1]Skeletal muscle mass / [2]Body fat mass / [3]Percentage body fat / [4]Visceral fat area/adipose tissue / [5]Hip circumference / [6]Waist to hip ratio / [7]Abdominal fat / [8]Subcutaneous adipose tissue / [9]Waist circumference / [10]Waist circumference reduction likelihood	[1]HbA1c / [2]FPG / [3]2-h PG / [4]PPGRs 5 hours / [5]Glucose level time above 140 mg/dL / [6]Time in postprandial hyperglycemia / [7]Frequencies of postprandial hyperglycemia / [8]Mean glucose (CGM) / [9]HOMAIR / [10]HOMA-B / [11]AUC-glucose / [12]AUC-insulin / [13]Fasting insulin / [14]Matsuda index / [15]SD of interstitial glucose from CGM / [16] TAR / [17]TBR / [18]TIR / [19]Achieving a normal fasting glucose / [20]Achieving a normal HbA1c (<5.7%) / [21]CONGA at 2 hours / [22]MAGE	[1]AST / [2]ALT / [3]Fatty Liver Index / [4]US Liver/ [5]Cholesterol/HDL ratio / [6]HDL / [7]LDL / [8]TC / [9]Triglycerides	[1]SBP / [2]DBP	Creatinine	Framingham 8-year diabetes risk score	[1]Yang-deficiency, [2]Yin-deficiency, [3]Phlegm-stasis
Bender, 2018 [a]	▲[^1] ▲[^2]	▲[^9]	▲[1] ▲[2]					
Ben-Yacov, 2021 [a]			▲[^4]	▲[1] ▲[2] ▲[^3] ▼[4]				
Ben-Yacov, 2021 [b]	▲[1] ▲[2]		▲[^1] ▼[2] ▲[3] ▲[^5] ▲[^8] ▼[9]	▲[^5] ▲[^6] ▼[7] ▼[8] ▲[^9]	▼[1] ▲[2]			
Block, 2015 [a]	▲[^3] ▲[^6]		▲[^19]				▲[^]	
Block, 2015 [b]	▲[○1] ▲[○2]	▲[○9]	▲[○1] ▲[○2]					
Chen, 2020 [a]		▲[1] ▲[^2] ▲[^3] ▲[^4]	▲[^6] ▲[7]					
Chen, 2020 [b]	▲[^1] ▲[^2]		▲[1] ▲[^5]	▲[6] ▼[7] ▲[8] ▲[^9]				
Chung 2020 [b]	▲[2]		▲[1] ▼[2]					▲[1] ▼[2] ▲[3]
Fukuoka, 2015 [a]		▲[*^5]						
Fukuoka, 2015 [b]	▲[○1] ▲[○2]		▲[○1] ▲[○2]	▼[○6] ▲[○7] ▲[○8] ▲[○9]	▲[○1] ▲[○2]			
Karvela, 2024 [a]		▼[2]	▼[5]					
Karvela, 2024 [b]	▼[1] ▼[2]	▼[9]	▲[1] ▲[2] ▼[3] ▼[9]	▲[6] ▲[8] ▼[9]	▲[1] ▲[2]			
Katula, 2021 [a]	▲[^3]		▲[20]					
Katula, 2021 [b]	▲[^1]		▲[^1]	▲[^5] ▲[^6] ▲[^7] ▲[^8] ▲[9]	▼[1] ▼[2]			
Kitazawa, 2023 [a]	▲[4]		▲[^17] ▲[^18] ▼[21] ▲[22]					
Kitazawa, 2023 [b]	▲[^1] ▲[^2]	▲[^9]	▲[1] ▲[5] ▼[8]		▲[1] ▲[^2]			
Lakka 2023 [b]	▲[1] ▲[2]	▲[9]	▲[1] ●[2] ▼[3] ▲[○13]					
Lim, 2022 [b]	▲[^1] ▲[^2]		▲[^1] ▲[^2]	▲[^6] ▼[7] ▼[8] ▲[9]	▲[1] ▲[2]	▲[^]		

Luo, 2022 [a]		▲[7]▼[4]▲[8]	▲[11]▲[12]▲[14]▼[15]▼[16]▲[*17]					
Luo, 2022 [b]	●[°1]		▲[°2]▲[°13]	●[°6]▼[°7]▼[°8]▲[°9]	▲[°1]▲[°2]			
Mcleod, 2020 [b]	▲[1]		●[°1]					
Staite, 2020 [a]		●[6]						
Staite, 2020 [b]	▼[1]	▲[9]	▼[°1]	▼[°5]●[°6]▼[°7]▼[°8]▼[°9]	▲[°1]▲[°2]			
Toro-Ramos, 2020 [b]	▲[^1]▲[^2]		▼[1]					
Xu, 2020 [a]	▼[5]	▲[10]						
Xu, 2020 [b]	▼[2]	▼[9]						

Notes: The arrows show the effect direction towards improvement or worsening of the outcome in the intervention group in comparison with the control group. More than one arrow can be under one outcome in a study, if the study used several instruments to measure this outcome.

† a - only synthesized by effect directions due to heterogeneous outcome reporting; b -included in meta-analysis

▲: effect direction towards better outcome in the intervention than in the control group; ▲: effect direction towards less worse outcome in the intervention than in the control group; ▼: effect direction towards better outcome in the control than in the intervention group; ●: null effect where the difference between the effect in the intervention and in the control groups is 0.0; N/A: effect estimates not reported thereby effect direction could not be derived; * statistically significant at post intervention through p-value or CI; ^ statistically significant in difference in means between groups (DiD) through p-value or CI; ° statistical significance neither in p-value nor in CI reported.

Abbreviations: ALT: Alanine transaminase; AST: Aspartate transferase; AUC: Area under the curve; BMI: Body Mass Index; CGM: Continuous glucose monitoring; CONGA: Continuous overlapping net glycemic action; DBP: Diastolic blood pressure; FPG: Fasting plasma glucose; HbA1c: Glycated hemoglobin; HDL: High density lipoprotein; HOMAIR: Homeostasis model assessment of insulin resistance; HOMA-B: Homeostasis model assessment of β-cell function; LDL: Low density lipoprotein; MAGE: Mean amplitude of glucose excursion; PPGR: Postprandial (post meal) glucose responses; SD: Standard deviation; SBP: Systolic blood pressure; TAR: Time above range; TBR: Time below range; TC: Total cholesterol; TIR: Time in range

Appendix S12: Macronutrient intake effect descriptions of longest follow-up

First author, year	Macronutrients				
	[1]Carbohydrate Intake / [2]Carbohydrate Proportion	[1]Protein Intake / [2]Protein Proportion	[1]Total Fat Intake / [2]Total Fat Proportion	[1]Saturated Fat Intake / [2]Saturated Fat Proportion	[1]Fiber Intake / [2]Fiber Proportion
Ben-Yacov, 2021	▼[*1]▼[*2]	▲[*1]▲[2]	▲[*1]▲[*2]	▲[*1]▲[*2]	▼[*1]▼[*2]
Fukuoka, 2015			▼[1]	▼[*^1]	
Karvela, 2024	▼[^1]		▲[1]	▲[1]	
Kitazawa, 2023	▼[^1]	▼[1]	▼[1]		
Lim, 2022	▼[^1]	▼[^1]	▼[^1]	▼[^1]	▼[1]
Xu, 2020	▼[1]▲[*2]	▼[*1]▼[2]	▼[*1]▼[*2]		

Notes: ▲: increased more in the intervention than in the control group; ▼: decreased more in the control than in the intervention group; * Statistically significant at post intervention through p-value or CI; ^ statistically significant in difference in means between groups (DiD) through p-value or CI; ° statistical significance neither in p-value nor in CI reported.

Appendix S13: Effect directions of psychosocial and behavioural outcomes at different follow-up times

Primary author, year	Outcome	Instrument/Unit	Effect direction (point estimate)		
			1 - ≤ 3 m	>3 - ≤ 6 m	>6 m
Chung, 2023	Dietary behaviour	Dietary approaches to stop hypertension (DASH), score (higher scores mean better correspondence to DASH diet)	↑ (0.22)	↓ (-0.51)	NA
	Physical activity	IPAQ (Taiwanese version), metabolic equivalents (MET)-minutes/week	↑ (236.33)	↑ (213.18)	NA
	Meredian body energy	Meridian Energy Analysis Device (MEAD), µA (MEAD values for the 24 acupoints (Ryodoraku points) along the 12 meridians ranging from 0 to 200 µA. Individuals with prediabetes have a lower level of body energy)	↓ (-0.16)	↓ (-5.84)	NA
	QoL: Physical component	SF-36 (Taiwanese version), score (higher scores indicate a better physical aspect of HRQOL)	↑ (2.56)	↑ (2.90)	NA
	QoL: Mental component	SF-36 (Taiwanese version), score (higher scores indicate a better mental aspect of HRQOL)	↑^ (4.63)	↑ (2.68)	NA
	Yang-deficiency	Score of Body composition questionnaire (BCQ), higher scores indicating a greater deficiency	↑ (-0.81)	↑ (-0.46)	NA
	Yin-deficiency	Score of Body composition questionnaire (BCQ), higher scores indicating a greater deficiency	↑ (-0.02)	↓ (0.03)	NA
	Phlegm-stasis	Score of Body composition questionnaire (BCQ), higher scores indicating a greater deficiency	↑ (-1.36)	↑ (-1.88)	NA
Fukuoka, 2015	Physical activity	7-Day Physical Activity Recall (PAR), kcal/kg/day	↑^ (1.8)	↑^ (0.3)	NA
	Physical activity	Pedometer, steps/day	↑*^ (3013)	↑*^ (3285)	NA
	Physical activity	Pedometer, steps/hour	↑*^ (142)	↑*^ (143)	NA
	Physical activity	Light-IPAQ, MET min/day	↑*^ (79)	↑* (66)	NA
	Physical activity	Moderate-IPAQ, MET min/day	↑*^ (22.7)	↑*^ (20.2)	NA
	Physical activity	Vigorous-IPAQ, MET min/day	↑ (1.26)	↑* (2.66)	NA
	Self-efficacy (barriers to being active, and social support)	Self-Efficacy for Physical Activity Survey, score (higher scores indicate better self-efficacy)	↓ (-0.3)	↑ (1.0)	NA
	Self-efficacy (barriers to being active, and social support)	Lack of time - Barriers to Being Active Quiz, score (score of 5 or above in any category shows that this is an important barrier)	↑^ (-0.88)	↑*^ (-1.47)	NA
	Self-efficacy (barriers to being active, and social support)	Social influence - Barriers to Being Active Quiz, score (score of 5 or above in any category shows that this is an important barrier)	↑*^ (-1.29)	↑*^ (-0.82)	NA
	Self-efficacy (barriers to being active, and social support)	Lack of energy - Barriers to Being Active Quiz, score (score of 5 or above in any category shows that this is an important barrier)	↑*^ (-0.99)	↑*^ (-1.15)	NA
	Self-efficacy (barriers to being active, and social support)	Lack of willpower - Barriers to Being Active Quiz, score (score of 5 or above in any category shows that this is an important barrier)	↑*^ (-1.88)	↑*^ (-2.4)	NA
	Self-efficacy (barriers to being active, and social support)	Fear of injury - Barriers to Being Active Quiz, score (score of 5 or above in any category shows that this is an important barrier)	↑ (-0.7)	↑ (-0.86)	NA
	Self-efficacy (barriers to being active, and social support)	Lack of skill - Barriers to Being Active Quiz, score (score of 5 or above in any category shows that this is an important barrier)	↑^ (-1.79)	↑^ (-1.26)	NA
	Self-efficacy (barriers to being active, and social support)	Lack of resources - Barriers to Being Active Quiz, score (score of 5 or above in any category shows that this is an important barrier)	↑ (-1.24)	↑ (-0.51)	NA
	Self-efficacy (barriers to being active, and social support)	Family - Social support for physical activity, score (higher scores indicate better support)	↑ (6.2)	↑ (3.0)	NA
	Self-efficacy (barriers to being active, and social support)	Friends - Social support for physical activity, score (higher scores indicate better support)	↑ (1.1)	↑ (2.8)	NA
	Hip circumference	cm	↑*^ (-2.4)	↑*^ (-6.5)	NA
Karvela, 2024	Fat mass	kg	↓ (1.01) †<br>↓ (1.39) ‡	↓ (1.79)	NA
	HOMA-B	N/A	↓ (17.74) †<br>↑ (-14.35) ‡	↓ (30.62)	NA

Katula, 2021	% in normal HbA1c (<5.7%) range	%	NA	↑^ (42.96%)	↑° (9.69%)
	5% Weight loss	%	NA	↑^ (34.24%)	↑^ (22.39%)
Lim, 2022	Diet, caloric intake	nBuddy dashboard's nutrient analysis platform, kcal/day	↑^ (-326.5)	↑^ (-397.1)	NA
	Diet, carbohydrate intake	nBuddy dashboard's nutrient analysis platform, g/day	↑^ (-42.2)	↑^ (-53.5)	NA
	Diet, sugar intake	nBuddy dashboard's nutrient analysis platform, g/day	↑^ (16.7)	↑^ (-19.0)	NA
	Diet, protein intake	nBuddy dashboard's nutrient analysis platform, g/day	↑ (-5.1)	↑ (-10.8)	NA
	Diet, total fat intake	nBuddy dashboard's nutrient analysis platform, g/day	↑^ (-15.4)	↑^ (-15.4)	NA
	Diet, saturated fat intake	nBuddy dashboard's nutrient analysis platform, g/day	↑^ (-7.6)	↑^ (-7.1)	NA
	Diet, fiber intake	nBuddy dashboard's nutrient analysis platform, g/day	↓ (-0.5)	↓ (-0.8)	NA
	Physical activity	nBuddy dashboard's nutrient analysis platform, g/day	↑ (42.5)	↑ (33.1)	NA
	Creatinine	umol/L	↑ (1.8)	↑^ (3.0)	NA
Luo, 2022	Abdominal fat	cm2	↑ (-9.5)	↑ (-0.4)	NA
	Visceral adipose tissue.	cm2	↑ (-5.1)	↓ (0.62)	NA
	Subcutaneous adipose tissue	cm2	↑ (-4.7)	↑ (-1.4)	NA
	AUC glucose	OGTT, mmol*h/L	↑ (-0.43)	↑ (-0.46)	NA
	AUC insulin	OGTT, uIU*h/mL	↓ (15.5)	↑ (-2.0)	NA
	Matsuda Index	OGTT, N/A	↑ (1.33)	↑ (0.78)	NA
	SD of interstitial glucose from CGM	CGM, mmol/L	↑ (N/A)	↓ (N/A)	NA
	Square root (Time above range (TAR)) (>10 mmol/L)	CGM. %	↑ (N/A)	↓ (N/A)	NA
	Square root (Time below range (TBR)) (<3.9 mmol/L)	CGM. %	↓ (N/A)	↑ (N/A)	NA
Staite, 2020	Physical activity	Wristband, mean steps/day	NA	↓ (-382.9)	↓ (-92.64)
	Physical activity	IPAQ, sitting minutes	NA	↓° (30.0)	↓° (30.0)
	Physical activity	IPAQ, total activity, metabolic equivalent of tasks (MET) min/week	NA	↑° (266)	↓° (-101.0)
Xu, 2020	Diet, energy intake	FFQ25, kcal/day	↓ (289)	↑* (-841)	NA
	Diet, carbohydrate intake	FFQ25, g/day	● (0.0)	↑ (-35.1)	NA
	Diet, carbohydrate intake proportion	FFQ25, %	↓ (-0.5%)	↑* (3.4%)	NA
	Diet, protein intake	FFQ25, g/day	↓ (2.2)	↑* (-28.2)	NA
	Diet, protein intake proportion	FFQ25, %	↓ (N/A)	↑ (N/A)	NA
	Diet, fat intake	FFQ25, g/day	↑ (-0.45)	↑* (-36.9)	NA
	Diet, fat intake proportion	FFQ25, %	↑ (N/A)	↑* (N/A)	NA
	Physical activity	IPAQ (Chinese version), metabolic equivalent of tasks (MET) min/week	↑ (618)	↑ (1353)	NA
	Stage of dietary behaviors change	Stage of change (SOC) scale, n (%)	↑*(N/A)	↑* (N/A)	NA
	Stage of physical activity change	Stage of change (SOC) scale, n (%)	↑ (N/A)	↑ (N/A)	NA

Notes:
Outcomes with only one follow-up time-point measurement and outcomes that are meta-analyzed are not included in this table.
↑: *Effect direction towards better outcome in the intervention than in the control group.*
↑: *Effect direction towards less worsened outcome in the intervention than in the control group.*
↓: *Effect direction towards better outcome in the control than in the intervention group.*
●: *Null effect where the difference between the effect in the intervention and in the control groups is 0.0.*
* *Statistically significant at post intervention through p-value or confidence interval.*
^ *Statistically significant in difference in means between groups (DiD) through p-value or confidence interval.*
° *Statistical significance neither in p-value nor in CI reported.*
† *Follow-up at 1.5 months.*
‡ *Follow-up at 3 months.*
Abbreviations: AUC: area under the curve; CGM: continuous glucose monitoring; FFQ: food frequency questionnaire; HbA1c: glycated hemoglobin; HOMA-B: Homeostasis model assessment of β-cell function; HRQOL: health-related quality of life; IPAQ: international physical activity questionnaire; N/A: effect

estimates not reported (thereby effect direction not illustrated); NA: not available; OGTT: oral glucose tolerance test; SD: standard deviation; SF-36: short form health survey.

Appendix S14: Meta-analyses results overview

Risk factor		N RCTs	N participants	MD (95% CI)	Tau (95% CI)	I^2
**Measures of anthropometry**	Body weight, kg	13	3950	-1.35 [-2.48; -0.23]	1.75 [0.83; 3.22]	98.6%
	Body weight, %	6	1428	-2.68 [-4.86; -0.50]	1.07 [0.74; 4.25]	90.4%
	Body mass index, kg/m^2	11	3045	-0.53 [-0.97; -0.09]	0.61 [0.29; 1.19]	98.5%
	Waist circumference, cm	6	2376	-1.05 [-3.34; 1.23]	2.34 [1.17; 5.94]	99.1%
**Measures of blood glucose**	HbA1c, %	11	2158	-0.08 [-0.10; -0.05]	0.01 [0.00; 0.07]	11.1%
	HbA1c, mmol/mol	7	3151	-0.54 [-1.14; 0.05]	0.44 [0.28; 1.33]	88.1%
	Fasting Plasma Glucose (FPG), mmol/L	9	2803	-0.11 [-0.24; 0.01]	0.13 [0.08; 0.31]	93.8%
	2h Post Glucose, mmol/L	3	1855	0.03 [-0.06; 0.11]	0 [0.00; 0.34]	0.0%
	Glucose level time >140 mg/dL, h/Day	3	441	-0.60 [-1.89; 0.69]	0.53 [0.13; 3.46]	84.1%
	Fasting insulin, mIU/L	3	2011	-0.23 [-2.67; 2.21]	0.75 [0.00; 6.37]	51.5%
	Mean Glucose, mg/dL	2	341	-2.05 [-42.30; 38.21]	4.30	92.0%
	HOMAIR	2	280	0.29 [0.27; 0.31]	0	0.0%
**Measures of blood lipids**	LDL cholesterol, mmol/L	7	1532	0.02 [-0.06; 0.10]	0.03 [0.00; 0.18]	9.6%
	HDL cholesterol, mmol/L	8	1589	0.03 [-0.01; 0.07]	0.02 [0.01; 0.09]	59.7%
	Total cholesterol, mmol/L	8	1589	-0.03 [-0.14; 0.08]	0.07 [0.00; 0.59]	50.6%
	Cholesterol/HDL ratio	3	978	-0.09 [-0.47; 0.29]	0.12 [0.01; 0.94]	71.1%
	Triglycerides, mmol/L	8	1589	-0.09 [-0.25; 0.08]	0.14 [0.07; 0.39]	76.3%
**Measures of blood pressure**	Systolic blood pressure, mmHg	8	1593	-1.35 [-3.09; 0.39]	1.33 [0.01; 3.93]	57.7%
	Diastolic blood pressure, mmHg	8	1621	-1.19 [-2.46; 0.08]	0.99 [0.00; 2.95]	58.8%

Abbreviations: HbA1c: glycated hemoglobin; HDL: high density lipoprotein; HOMAIR: Homeostasis model assessment of insulin resistance; LDL: low density lipoprotein.

Appendix S15: Meta-analysis SBP (mmHg)

**Subgroup**	**N RCTs**	**MD (95% CI)**	**I^2**
**Overall**	8	-1.35 [-3.09; 0.39]	57.7%
**Type of control group**			
Usual care	2	-1.82 [-3.18; -0.45]	0.0%
Enhanced usual care	1	1.07 [-1.29; 3.43]	-
App and/or device	3	-1.85 [-5.07; 1.38]	64.4%
**Degree of behavioral support**			
Face-to-face support	3	-1.99 [-10.90; 6.92]	81.3%
No face-to-face support	5	-1.25 [-2.74; 0.25]	31.3%
**Follow-up**			
Short (≤3 months)	1	-1.38 [-4.45; 1.69]	-
Medium (>3 to ≤6 months)	4	-1.70 [-6.35; 2.95]	72.5%
Long (>6 months)	3	-1.08 [-5.58; 3.42]	64.2%
**Inclusion criteria**			
Prediabetes	2	-0.70 [-18.57; 17.17]	75.6%
Prediabetes and increased BMI	6	-1.7 [-4.16; 0.77]	59.5%
**Risk of bias**			
Low	1	-2.62 [-6.37; 1.13]	-
Some	7	-1.24 [-3.24; 0.77]	63.0%
High	-	-	-

Appendix S16: Meta-analysis DBP (mmHg)

**Subgroup**	**N RCTs**	**MD (95% CI)**	**I^2**
**Overall**	8	-1.19 [-2.46; 0.08]	57.8%
**Type of control group**			
Usual care	2	-1.37 [-16.54; 13.80]	77.5%
Enhanced usual care	1	0.25 [-1.47; 1.97]	-
App and/or device	5	-1.62 [-3.74; 0.50]	50.4%
**Degree of behavioral support**			
Face-to-face support	3	-2.08 [-7.81; 3.66]	70.8%
No face-to-face support	5	-0.72 [-1.95; 0.51]	34.1%
**Follow-up**			
Short (≤3 months)	1	-2.84 [-5.06; -0.62]	-

Medium (>3 to ≤6 months)	4	-1.65 [-4.81; 1.51]	62.7%
Long (>6 months)	3	-0.40 [-1.29; 0.49]	0.0%
**Inclusion criteria**			
Prediabetes	2	-0.41 [-0.79; -0.02]	0.0%
Prediabetes and increased BMI	6	-1.66 [-3.53; 0.21]	60.1%
**Risk of bias**			
Low	1	-1.61 [-3.92; 0.70]	-
Some	7	-1.16 [-2.66; 0.34]	62.2%
High	-	-	-

Appendix S17: Meta-analysis FPG (mmol/L)

**Subgroup**	**N RCTs**	**MD (95% CI)**	**I^2**
**Overall**	9	-0.11 [-0.24; 0.01]	93.8%
**Type of control group**			
Usual care	1	-0.24 [-0.87; 0.40]	94.6%
Enhanced usual care	2	0.02 [-0.74; 0.79]	24.5%
App and/or device	5	-0.10 [-0.31; 0.10]	61.5%
**Degree of behavioral support**			
Face-to-face support	5	-0.07 [-0.34; 0.20]	69.4%
No face-to-face support	4	-0.14 [-0.35; 0.06]	97.3%
**Follow-up**			
Short (≤3 months)	1	-0.08 [-0.34; 0.19]	-
Medium (>3 to ≤6 months)	6	-0.12 [-0.34; 0.10]	87.6%
Long (>6 months)	2	-0.10 [-1.30; 1.11]	98.4%
**Inclusion criteria**			
Prediabetes	4	-0.03 [-0.26; 0.20]	95.8%
Prediabetes and increased BMI	5	-0.18 [-0.39; 0.02]	83.8%
**Risk of bias**			
Low	2	-0.23 [-1.47; 1.01]	59.0%
Some	7	-0.08 [-0.24; 0.07]	92.5%
High	-	-	-

Appendix S18: Meta-analysis HDL Cholesterol (mmol/L)

**Subgroup**	**N RCTs**	**MD (95% CI)**	**I^2**
**Overall**	8	0.03 [-0.01; 0.07]	59.7%

**Type of control group**			
Usual care	1	0.02 [0.01; 0.03]	-
Enhanced usual care	1	0.05 [0.02; 0.08]	-
App and/or device	6	0.02 [-0.04; 0.09]	63.9%
**Degree of behavioral support**			
Face-to-face support	4	0.03 [-0.10; 0.17]	73.4%
No face-to-face support	4	0.02 [-0.00; 0.05]	28.8%
**Follow-up**			
Short (≤3 months)	1	0.06 [-0.03; 0.15]	-
Medium (>3 to ≤6 months)	4	0.02 [-0.11; 0.15]	76.1%
Long (>6 months)	3	0.03 [-0.02; 0.07]	46.9%
**Inclusion criteria**			
Prediabetes	3	0.04 [-0.03; 0.12]	56,1%
Prediabetes and increased BMI	5	0.01 [-0.06; 0.09]	76.4%
**Risk of bias**			
Low	1	0.00 [-0.07; 0.07]	-
Some	6	0.03 [-0.02; 0.08]	69.1%
High	1	0.06 [-0.03; 0.15]	-

Appendix S19: Meta-analysis total Cholesterol (mmol/L)

Subgroup	N RCTs	MD (95% CI)	I^2
**Overall**	8	-0.03 [-0.14; 0.08]	50.6%
**Type of control group**			
Usual care	1	-0.07 [-0.10; -0.04]	-
Enhanced usual care	1	-0.01 [-0.12; 0.09]	-
App and/or device	6	-0.02 [-0.22; 0.19]	57.6%
**Degree of behavioral support**			
Face-to-face support	4	-0.13 [-0.44; 0.17]	27.1%
No face-to-face support	4	0.01 [-0.14; 0.15]	64.0%
**Follow-up**			
Short (≤3 months)	1	-0.10 [-0.39; 0.19]	-
Medium (>3 to ≤6 months)	4	-0.03 [-0.47; 0.41]	71.0%
Long (>6 months)	3	-0.04 [-0.19; 0.12]	39.2%
**Inclusion criteria**			
Prediabetes	3	-0.07 [-0.14; -0.00]	0.0%

Prediabetes and increased BMI	5	-0.00 [-0.19; 0.18]	58.9%
**Risk of bias**			
Low	1	0.10 [-0.11; 0.31]	-
Some	6	-0.04 [-0.18; 0.11]	57.9%
High	1	-0.10 [-0.39; 0.19]	-

Appendix S20: Meta-analysis triglycerides (mmol/L)

**Subgroup**	**N RCTs**	**MD (95% CI)**	**I^2**
**Overall**	8	-0.09 [-0.25; 0.08]	76.3%
**Type of control group**			
Usual care	1	0.02 [0.00; 0.04]	-
Enhanced usual care	1	-0.05 [-0.22; 0.11]	-
App and/or device	6	-0.12 [-0.38; 0.14]	66.6%
**Degree of behavioral support**			
Face-to-face support	4	-0.24 [-0.32; -0.16]	0.0%
No face-to-face support	4	0.03 [-0.12; 0.18]	30.7%
**Follow-up**			
Short (≤3 months)	1	-0.36 [-0.68; -0.04]	-
Medium (>3 to ≤6 months)	4	-0.15 [-0.41; 0.11]	55.5%
Long (>6 months)	3	0.02 [-0.30; 0.35]	48.5%
**Inclusion criteria**			
Prediabetes	3	-0.15 [-0.62; 0.32]	86.8%
Prediabetes and increased BMI	5	-0.04 [-0.33; 0.24]	65.0%
**Risk of bias**			
Low	1	0.39 [-0.03; 0.81]	-
Some	6	-0.09 [-0.24; 0.06]	76.4%
High	1	-0.36 [-0.68; -0.04]	-

Appendix S21: Meta-analysis HbA1c (mmol/mol)

**Subgroup**	**N RCTs**	**MD (95% CI)**	**I^2**
**Overall**	7	-0.54 [-1.14; 0.05]	88.1%
**Overall (excl. Lakka after leave-one out robustness check)**	6	-0.68 [-1.33; -0.03]	75%
**Type of control group**			
Usual care	3	-0.85 [-1.44; -0.26]	46.1%

Enhanced usual care	2	-0.41 [-5.16; 4.33]	85.7%
App and/or device	2	-0.51 [-14.04; 13.02]	91.6%
**Degree of behavioral support**			
Face-to-face support	1	-1.60 [-2.57; -0.63]	-
No face-to-face support	6	-0.45 [-1.05; 0.16]	89.4%
**Follow-up**			
Short (≤3 months)	-	-	-
Medium (>3 to ≤6 months)	2	-1.08 [-5.14; 2.99]	50.4%
Long (>6 months)	5	-0.31 [-1.05; 0.43]	90.3%
**Inclusion criteria**			
Prediabetes	4	-0.60 [-1.71; 0.52]	91.4%
Prediabetes and increased BMI	3	-0.47 [-2.35; 1.42]	85.4%
**Risk of bias**			
Low	3	-0.17 [-1.99; 1.66]	87.9%
Some	4	-0.75 [-1.69; 0.18]	91.0%
High	-	-	-

Appendix S22: Meta-analysis waist circumference (cm)

Subgroup	N RCTs	MD (95% CI)	I^2
**Overall**	6	-1.05 [-3.34; 1.23]	99.1%
**Type of control group**			
Usual care	1	-0.49 [-5.65; 4.66]	99.6%
Enhanced usual care	3	-0.10 [-0.56; 0.36]	-
App and/or device	2	-2.67 [-29.97; 24.63]	86.7%
**Degree of behavioral support**			
Face-to-face support	1	-4.90 [-7.35; -2.45]	-
No face-to-face support	5	-0.43 [-2.30; 1.43]	99.2%
**Follow-up**			
Short (≤3 months)	2	-2.72 [-28.11; 22.66]	89.1%
Medium (>3 to ≤6 months)	1	-2.34 [-2.53; -2.15]	-
Long (>6 months)	3	0.47 [-2.60; 3.54]	95.7%
**Inclusion criteria**			
Prediabetes	2	0.83 [-10.86; 12.52]	97.7%
Prediabetes and increased BMI	4	-1.94 [-4.57; 0.69]	83.2%
**Risk of bias**			

Low	3	-2.42 [-7.19; 2.34]	73.6%
Some	3	0.28 [-3.07; 3.64]	96.8%
High	-	-	-

Appendix S23: Meta-analysis LDL Cholesterol (mmol/L)

**Subgroup**	**N RCTs**	**MD (95% CI)**	**I^2**
**Overall**	7	0.02 [-0.06; 0.10]	9.6%
**Type of control group**			
Usual care	-	-	-
Enhanced usual care	1	-0.05 [-0.15; 0.05]	-
App and/or device	6	0.06 [-0.04; 0.15]	0.0%
**Degree of behavioral support**			
Face-to-face support	4	0.04 [-0.13; 0.22]	11.8%
No face-to-face support	3	0.01 [-0.20; 0.22]	29.9%
**Follow-up**			
Short (≤3 months)	1	0.15 [-0.11; 0.41]	-
Medium (>3 to ≤6 months)	4	0.05 [-0.11; 0.21]	12.5%
Long (>6 months)	2	-0.04 [-0.38; 0.31]	0.0%
**Inclusion criteria**			
Prediabetes	2	0.13 [-0.05; 0.30]	0.0%
Prediabetes and increased BMI	5	-0.01 [-0.10; 0.08]	0.0%
**Risk of bias**			
Low	1	0.02 [-0.19; 0.23]	-
Some	5	0.01 [-0.10; 0.13]	27.6%
High	1	0.15 [-0.11; 0.41]	-

Appendix S24: Meta-analysis Weight (%)

**Subgroup**	**N RCTs**	**MD (95% CI)**	**I^2**
**Overall**	6	-2.68 [-4.86; -0.50]	90.4%
**Type of control group**			
Usual care	1	-2.28 [-2.33; -2.23]	-
Enhanced usual care	1	-3.40 [-4.37; -2.44]	-
App and/or device	4	-2.96 [-7.61; 1.69]	92.1%
**Degree of behavioral support**			
Face-to-face support	4	-2.96 [-7.61; 1.69]	92.1%

No face-to-face support	2	-2.73 [-9.71; 4.25]	80.6%
**Follow-up**			
Short (≤3 months)	1	-1.10 [-1.69; -0.52]	-
Medium (>3 to ≤6 months)	4	-3.19 [-7.40; 1.02]	90.4%
Long (>6 months)	1	-3.40 [-4.37; -2.44]	-
**Inclusion criteria**			
Prediabetes	1	-0.59 [-1.72; 0.55]	-
Prediabetes and increased BMI	5	-3.09 [-5.59; -0.60]	90.8%
**Risk of bias**			
Low	2	-1.73 [-9.21; 5.75]	93.6%
Some	4	-3.61 [-7.78; 0.56]	90.8%
High	-	-	-

Appendix S25: Effect estimates over time

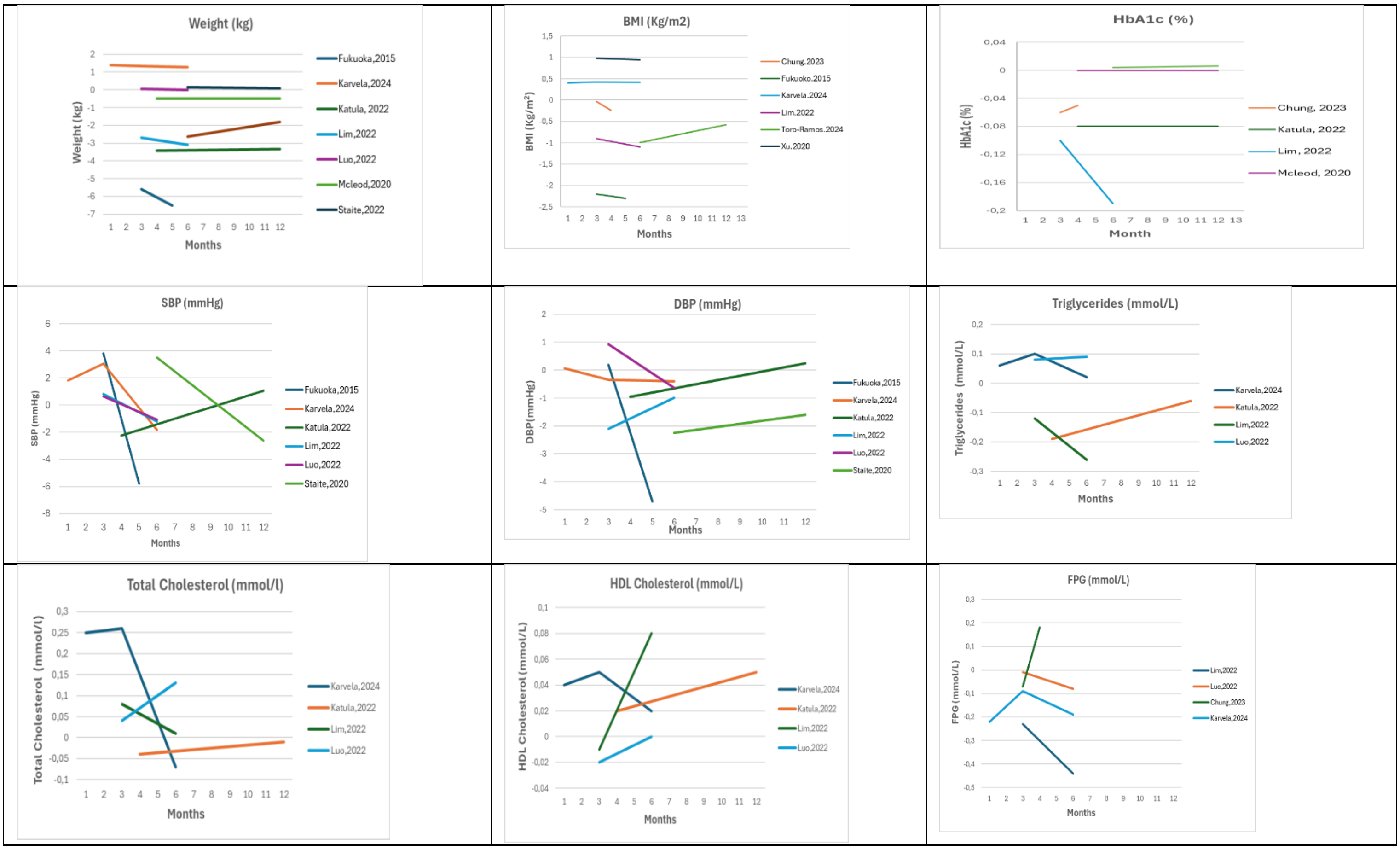

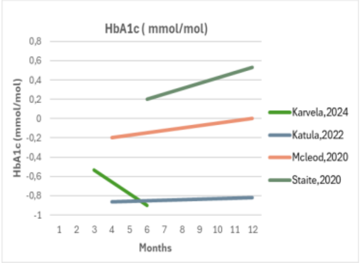

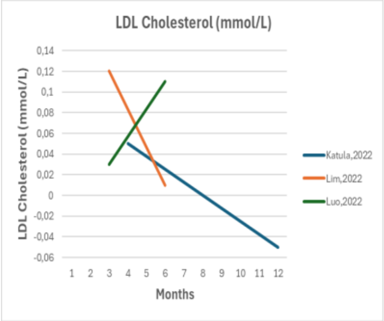

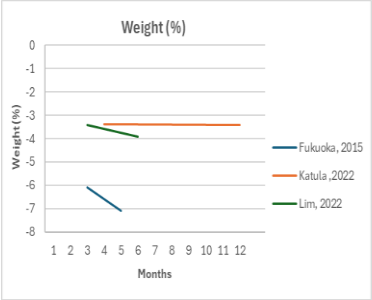

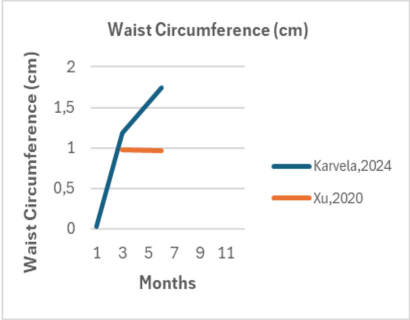

Abbreviations: BMI: body mass index; DBP: diastolic blood pressure; FPG: fasting plasma glucose; HbA_{1c}: glycated hemoglobin; HDL: high density lipoprotein; LDL: low density lipoprotein; SBP: systolic blood pressure.

Appendix S26: Sensitivity analysis of follow-up effectiveness by <12 months vs. 12 months

Risk factor	N RCTs	MD (95% CI)	Tau (95% CI)	I^2
**Measures of anthropometry**				
Body weight, kg - Total	13	-1.34 [-2.44; -0.25]	1.91 [0.87; 3.40]	99.1%
Body weight, kg - FU<12m	8	-1.48 [-3.25; 0.29]	2.08	99.4%
Body weight, kg - FU 12m	5	-1.12 [-2.89; 0.64]	1.29	88.2%
Body weight, % - Total	6	-2.66 [-4.73; -0.59]	1.06 [0.71; 4.03]	90.5%
Body weight, % - FU<12m	5	-2.53 [-5.26; 0.19]	1.13	91.6%
Body weight, % - FU 12m	1	-3.4 [-4.37; -2.44]	-	-
Body mass index, kg/m^2 - Total	11	-0.52 [-0.96; -0.08]	0.65 [0.30; 1.25]	99.0%
Body mass index, kg/m2 - FU<12m	9	-0.58 [-1.13; -0.02]	0.70	99.1%
Body mass index, kg/m2 - FU 12m	2	-0.27 [-3.51; 2.97]	0.32	76.9%
Waist circumference, cm - Total	6	-1.16 [-3.21; 0.90]	2.02 [1.00; 5.22]	99.0%
Waist circumference, cm - FU<12m	4	-1.60 [-5.41; 2.21]	2.25	99.4%
Waist circumference, cm - FU 12m	2	-0.13 [-1.61; 1.36]	0	0.0%
**Measures of blood glucose**				
HbA1c, % - Total	11	-0.07 [-0.09; -0.05]	0.01 [0.00; 0.14]	7.1%
HbA1c, % - FU<12m	7	-0.08 [-0.09; -0.06]	0	0.0%
HbA1c, % - FU 12m	4	-0.05 [-0.28; 0.18]	0.08	64.7%

HbA1c, mmol/mol - Total	7	-0.51 [-1.08; 0.05]	0.38 [0.26; 1.25]	86.3%
HbA1c, mmol/mol - FU<12m	3	-0.83 [-1.40; -0.25]	0.14	47.4%
HbA1c, mmol/mol - FU 12m	4	-0.14 [-1.01; 0.74]	0.43	52.6%
FPG, mmol/L - Total	9	-0.11 [-0.21; -0.0023]	0.12 [0.07; 0.28]	93.3%
FPG, mmol/L - FU<12m	8	-0.13 [-0.24; -0.02	0.09	86.0%
FPG, mmol/L – FU 12m	1	0.00 [-0.04; 0.04]	-	-
**Measures of blood lipids**				
LDL cholesterol, mmol/L - Total	7	0.01 [-0.06; 0.09]	0.00 [0.00; 0.16]	0.0%
LDL cholesterol, mmol/L - FU<12m	5	0.06 [-0.06; 0.17]	0	0.0%
LDL cholesterol, mmol/L – FU 12m	2	-0.04 [-0.38; 0.31]	0	0.0%
HDL cholesterol, mmol/L - Total	8	0.03 [-0.005; 0.06]	0.02 [0.01; 0.09]	52.3%
HDL cholesterol, mmol/L - FU<12m	6	0.02 [-0.03; 0.08]	0.03	61.1%
HDL cholesterol, mmol/L – FU 12m	2	0.03 [-0.25; 0.32]	0.02	42.9%
Total cholesterol, mmol/L - Total	8	0.02 [-0.11; 0.15]	0.12 [0.00; 0.61]	74.0%
Total cholesterol, mmol/L - FU<12m	6	0.00 [-0.21; 0.21]	0.15	75%
Total cholesterol, mmol/L – FU 12m	2	0.01 [-0.57; 0.59]	0	0.0%
Triglycerides, mmol/L – Total	8	-0.08 [-0.25; 0.09]	0.16 [0.08; 0.41]	81.4%
Triglycerides, mmol/L - FU<12m	6	-0.12 [-0.13; 0.06]	0.17	85.0%
Triglycerides, mmol/L – FU 12m	2	0.12 [-2.65; 2.90]	0.27	73.9%
**Measures of blood pressure**				
Systolic blood pressure, mmHg - Total	8	0.52 [-0.33; 1.37]	0.36 [0.00; 2.34]	9.0%
Systolic blood pressure, mmHg - FU<12m	6	0.77 [0.05; 1.48]	0	0%
Systolic blood pressure, mmHg – FU 12m	2	-0.48 [-23.61; 22.66]	2.06	62.5%
Diastolic blood pressure, mmHg - Total	8	-0.66 [-1.51; 0.19]	0.51 [0.00; 2.08]	27.6%
Diastolic blood pressure, mmHg - FU<12m	6	-0.81 [-1.97; 0.36]	0.66	38.0%
Diastolic blood pressure, mmHg – FU 12m	2	-0.51 [-12.13; 11.11]	0.80	37.4%

Appendix S27: PROGRESS-Plus effect directions

Subgroup Category	Primary Author, Year	Subgroup	Outcome (unit)	Follow-up (month)	Type of analysis	Effect estimate	CI/P-value	Effect direction (summary and interpretation +/-) †
Ethnicity	Mcleod, 2020	Maori/Pacific	Weight (kg)	12	DiD	1.9	(-1.9; 5.7) / P = 0.432	Increased in the intervention group and decreased in the control group. -
**Gender**	**Ben-Yacov, 2021**	**Male**	**Glucose level time above 140 mg/dL (hour/day)**	**6**	**DiD**	**-1.000**	**(-1.57; -0.43) / P < 0.001**	**Decreased more in the PPT-diet group than in the MED-diet group. +**
**Gender**	**Ben-Yacov, 2021**	**Female**	**Glucose level time above 140 mg/dL (hour/day)**	**6**	**DiD**	**-0.995**	**(-1.36; -0.63) / P <0.001**	**Decreased more in the PPT-diet group than in the MED-diet group. +**
Gender	Ben-Yacov, 2021	Male	HbA1c (%)	6	DiD	-0.08	(-0.17; 0.01) / P = n.s.	Decreased more in the PPT-diet group than in the MED-diet group. +
**Gender**	**Ben-Yacov, 2021**	**Female**	**HbA1c (%)**	**6**	**DiD**	**-0.08**	**(-0.15; -0.01) / P <0.05**	**Decreased more in the PPT-diet group than in the MED-diet group. +**
Gender	Ben-Yacov, 2021	Male	OGTT (mmol/mol)	6	DiD	0.562	(-0.51; 1.63) / P = n.s.	Increased more in the PPT-diet group than in the MED-diet group. -
Gender	Ben-Yacov, 2021	Female	OGTT (mmol/mol)	6	DiD	-0.646	(-1.47; 0.18) / P = n.s.	Decreased more in the PPT-diet group than in the MED-diet group. +
Gender	Karvela, 2024	Male	FPG (mmol/l)	6.5	Linear mixed effects	-0.15	(-0.74; 0.44) / P = 0.06	Decreased more in the intervention group than in the control group. +
Gender	Karvela, 2024	Female	FPG (mmol/l)	6.5	Linear mixed effects	0.24	(-0.25; 0.73) / P = 0.23	Increased more in the intervention group than in the control group. -
Gender	Luo, 2022	Male	0-h Glucose (mmol/l)	6	Within group comparison	MD: -0.41; TJD: -0.35; CD: -0.3	MD: (-0.52; -0.3); TJD: (-0.46; -0.24); CD: (-0.41; -0.19); / P = 0.37 (DiD)	Decreased more in the MD-group than in the CD-group. +
Gender	Luo, 2022	Female	0-h Glucose (mmol/l)	6	Within group comparison	MD: -0.22; TJD: -0.19; CD: -0.22	MD: (-0.52; 0.09); TJD: (-0.52; 0.14); CD: (-0.58; 0.13) / P =0.46 (DiD)	Null effect
Gender	Luo, 2022	Male	0-h insulin (uIU/mL)	6	Within group comparison	MD: -4.91; CD: -4.58; TJD: -4.19	MD: (-6.81; -3.01); CD: (-6.49; -2.68); TJD: (-6.05; -2.33) / P = 0.84 (DiD)	Decreased more in the MD-group than in the CD-group. +
Gender	Luo, 2022	Female	0-h insulin (uIU/mL)	6	Within group comparison	MD: -3.25; TJD: -2.56; CD: -3.25	MD: (-5.67; -0.84); TJD: (-5.17; 0.06); CD: (-6.07; -0.44) / P = 0.33 (DiD)	Null effect
Gender	Luo, 2022	Male	AUC Glucose (mmol*h/L)	6	Within group comparison	MD: -0.91; TJD: -0.23; CD: -0.45	MD: (-1.48; -0.33); TJD: (-0.8; 0.34); CD: (-1; 0.1) / P = 0.92 (DiD)	Decreased more in the MD-group than in the CD-group. +

Gender	Luo, 2022	Female	AUC Glucose (mmol*h/L)	6	Within group comparision	MD: -1.11; TJD: 0.09; CD: -0.55	MD: (-2.71; 0.49); TJD: (-1.78; 1.95); CD: (-2.49; 1.39) / P = 0.89 (DiD)	Decreased more in the MD-group than in the CD-group. +
Gender	Luo, 2022	Male	AUC insulin (uIU*h/mL)	6	Within group comparison	MD: -31.2; TJD: -26.5; CD: -23.0	MD: (-52.6; -9.8); TJD: (-47.7; -5.17); CD: (-43.2; -2.84) / P = 0.11 (DiD)	Decreased more in the MD-group than in the CD-group. +
Gender	Luo, 2022	Female	AUC insulin (uIU*h/mL)	6	Within group comparison	MD: -9.30; TJD: -43.3; CD: -45.6	MD: (-56.4; 37.8); TJD: (-99.8; 13.31); CD: (-106.1; 14.98) / P = 0.46 (DiD)	Decreased more in the CD-group than in the MD-group. -
Gender	Luo, 2022	Male	Mastuda Index (N/A)	6	Within group comparison	MD: 5.31; TJD: 3.94; CD: 4.05	MD: (3.45 ;7.17); TJD: (2.11; 5.77); CD: (2.33; 5.78) / P = 0.8 (DiD)	Increased more in the MD-group than in the CD-group. +
Gender	Luo, 2022	Female	Mastuda Index (N/A)	6	Within group comparison	MD: 2.57; TJD: 2.73; CD: 4.63	MD: (-0.56; 5.7); TJD: (-1.22; 6.69); CD: (0.63; 8.63) / P = 0.79 (DiD)	Increased more in the CD-group than in the MD-group.
Gender	Luo, 2022	Male	Mean Glucose (mmol/l)	6	Within group comparison	MD: -0.42; TJD: -0.38; CD: -0.67	MD: (-0.57; -0.27); TJD: (-0.53; -0.24); CD: (-0.82; -0.53) / P = 0.11 (DiD)	Decreased more in the CD-group than in the MD-group. -
Gender	Luo, 2022	Female	Mean Glucose (mmol/l)	6	Within group comparison	MD: -0.1; TJD: -0.8; CD: -0.41	MD: (-0.51; 0.31); TJD: (-1.21; -0.39); CD: (-0.93; 0.11) / P = 0.29 (DiD)	Decreased more in the CD-group than in the MD-group. -
Gender	Luo, 2022	Male	TAR (%)	6	Within group comparison	MD: -0.21; TJD: -0.26; CD: -0.41	MD: (-0.37; -0.04); TJD: (-0.42; -0.1); CD: (-0.57; -0.25) / P = 0.10 (DiD)	Decreased more in the CD-group than in the MD-group. -
Gender	Luo, 2022	Female	TAR (%)	6	Within group comparison	MD: 0.01; TJD: -0.47; CD: -0.25	MD: (-0.35; 0.37); TJD: (-0.83; -0.11); CD: (-0.69; 0.18) / P = 0.17 (DiD)	Decreased in the CD-group and increased in the MD-group. -
**Gender**	**Luo, 2022**	**Male**	**TBR, <3.9 mmol/l (%)**	**6**	**Within group comparison**	**MD: 0.4; TJD: 0.3; CD: 1.14**	**MD: (-0.06; -0.85); TJD: (-0.15; -0.74); CD: (0.7; 1.58) / P = 0.029 (DiD)**	**Increased less in the MD-group than in the CD-group. +**
Gender	Luo, 2022	Female	TBR, <3.9 mmol/l (%)	6	Within group comparison	MD: -0.69; TJD: 1.77; CD: 0.16	MD: (-1.73; 0.36); TJD: (0.73; 2.81); CD: (-1.11; 1.43) / P = 0.44 (DiD)	Decreased in the MD-group and increased in the CD-group. +
**Gender**	**Luo, 2022**	**Male**	**TBR level 1, 3-3.9 mmol/l (%)**	**6**	**Within group comparison**	**MD: 0.4; TJD: 0.34; CD: 1.16**	**MD: (-0.02; 0.82); TJD: (-0.08; 0.75); CD: (0.76; 1.57) / P = 0.013 (DiD)**	**Increased less in the MD-group than in the CD-group. +**
Gender	Luo, 2022	Female	TBR level 1, 3-3.9 mmol/l (%)	6	Within group comparison	MD: -0.52; TJD: 1.74; CD: 0.24	MD: (-1.52; 0.48); TJD: (0.74; 2.74); CD: (-0.98; 1.45) / P = 0.47 (DiD)	Decreased in the MD-group and increased in the CD-group. +
Gender	Luo, 2022	Male	TBR level 2, <3 mmol/l (%)	6	Within group comparison	MD: 0.04; TJD: -0.01; CD: 0.11	MD: (-0.19; -0.28); TJD: (-0.24; -0.22); CD: (-0.11; -0.34) / P = 0.98 (DiD)	Increased less in the MD-group than in the CD-group. +
Gender	Luo, 2022	Female	TBR level 2, <3 mmol/l (%)	6	Within group comparison	MD: -0.45; TJD: 0.36; CD: -0.16	MD: (-0.86; -0.04); TJD: (-0.06; 0.78); CD: (-0.68; 0.37) / P = 0.47 (DiD)	Decreased more in the MD-group than in the CD-group. +

Gender	Luo, 2022	Male	Nighttime SD (mmol/l)	6	Within group comparison	MD: -0.01; TJD: -0.14; CD: -0.11	MD: (-0.08; 0.05); TJD: (-0.21; -0.08); CD: (-0.17; -0.04) / P = 0.067 (DiD)	Decreased more in the CD-group than in the MD-group. -
Gender	Luo, 2022	Female	Nighttime SD (mmol/l)	6	Within group comparison	MD: -0.1; TJD: -0.05; CD: 0	MD: (-0.21; 0.01); TJD: (-0.16; 0.05); CD: (-0.14; 0.14) / P = 0.81 (DiD)	Decreased more in the MD-group than in the CD-group. +
Gender	Luo, 2022	Male	Weight (kg)	6	Within group comparison	MD:-5.82; TJD:-5.25; CD:-5.4	MD: (-6.59; -5.05); TJD: (-6; -4.5); CD: (-6.14;-4.65) / P = 0.44 (DiD)	Decreased more in the MD-group than in the CD-group. +
Gender	Luo, 2022	Female	Weight (kg)	6	Within group comparison	MD: -5.23; TJD: -3.74; CD: -5.56	MD: (-6.85; -3.61); TJD: (-5.44; -2.03); CD: (-7.47; -3.65) / P = 0.24 (DiD)	Decreased more in the CD-group than in the MD-group. -
Education	Block, 2015	Postgraduate or professional vs. lower educational level	N/A	6	N/A	N/A	N/A	"However, it is notable that the subgroup with postgraduate or professional degrees achieved less improvement in glycemic markers than those with lower educational levels (data not shown.)"
**Age**	**Ben-Yacov, 2021**	**≤50 years**	**Glucose level time above 140 mg/dL (hour/day)**	**6**	**DiD**	**-0.97**	**(-1.35; -0.59) / P <0.001**	**Decreased more in the PPT-diet group than in the MED-diet group. +**
**Age**	**Ben-Yacov, 2021**	**50 years <**	**Glucose level time above 140 mg/dL (hour/day)**	**6**	**DiD**	**-1.06**	**(-1.53;-0.59) / P <0.001**	**Decreased more in the PPT-diet group than in the MED-diet group. +**
Age	Ben-Yacov, 2021	≤50 years	HbA1c (%)	6	DiD	-0.075	(-0.16; 0.01) / P = n.s.	Decreased more in the PPT-diet group than in the MED-diet group. +
**Age**	**Ben-Yacov, 2021**	**50 years <**	**HbA1c (%)**	**6**	**DiD**	**-0.085**	**(-0.16; -0.01) / P <0.05**	**Decreased more in the PPT-diet group than in the MED-diet group. +**
Age	Ben-Yacov, 2021	≤50 years	OGTT (mmol/mol)	6	DiD	-0.052	(-1.0395; 0.935) / P = n.s.	Decreased more in the PPT-diet group than in the MED-diet group. +
Age	Ben-Yacov, 2021	50 years <	OGTT (mmol/mol)	6	DiD	-0.250	(-1.166; 0.6655) / P = n.s.	Decreased more in the PPT-diet group than in the MED-diet group. +
**Age**	**Katula, 2022**	**19 - 64**	**Body weight (%)**	**12**	**DiD**	**-2.815**	**(-3.91; -1.72) / P <0.001**	**Decreased more in the intervention group than in the control group. +**
**Age**	**Katula, 2022**	**65 ≤**	**Body weight (%)**	**12**	**DiD**	**-4.675**	**(-6.35; -3) / P < 0.001**	**Decreased more in the intervention group than in the control group. +**

**Age**	**Katula, 2022**	**19 - 64**	**Body weight (kg)**	**12**	**DiD**	**-2.835**	**(-4.02; -1.65) / P <0.001**	**Decreased more in the intervention group than in the control group.**<br>+
**Age**	**Katula, 2022**	**65 ≤**	**Body weight (kg)**	**12**	**DiD**	**-4.515**	**(-6.32; -2.71) / P < 0.001**	**Decreased more in the intervention group than in the control group.**<br>+
Age	Katula, 2022	19 - 64	HbA1c (%)	12	DiD	-0.06	(-0.11; -0.01) / P = 0.02	Decreased more in the intervention group than in the control group.<br>+
**Age**	**Katula, 2022**	**65 ≤**	**HbA1c (%)**	**12**	**DiD**	**-0.135**	**(-0.21; -0.06) / P<0.001**	**Decreased more in the intervention group than in the control group.**<br>+
Age	Katula, 2022	19 - 64	HbA1c (mmol/mol)	12	DiD	-0.66	(-1.19; -0.13) / P = 0.02	Decreased more in the intervention group than in the control group.<br>+
**Age**	**Katula, 2022**	**65 ≤**	**HbA1c (mmol/mol)**	**12**	**DiD**	**-1.485**	**(-2.31; -0.66) / P < 0.001**	**Decreased more in the intervention group than in the control group.**<br>+
**BMI**	**Ben-Yacov, 2021**	**BMI ≤ 25**	**Glucose level time above 140 mg/dL (hour/day)**	**6**	**DiD**	**-1.61**	**(-2.9; -0.32) / P <0.05**	**Decreased more in the PPT-diet group than in the MED-diet group.**<br>+
**BMI**	**Ben-Yacov, 2021**	**25 < BMI ≤ 30**	**Glucose level time above 140 mg/dL (hour/day)**	**6**	**DiD**	**-1.095**	**(-1.63; -0.56) / P <0.001**	**Decreased more in the PPT-diet group than in the MED-diet group.**<br>+
**BMI**	**Ben-Yacov, 2021**	**30 < BMI ≤ 35**	**Glucose level time above 140 mg/dL (hour/day)**	**6**	**DiD**	**-0.63**	**(-1.17; -0.09) / P <0.05**	**Decreased more in the PPT-diet group than in the MED-diet group.**<br>+
**BMI**	**Ben-Yacov, 2021**	**BMI > 35**	**Glucose level time above 140 mg/dL (hour/day)**	**6**	**DiD**	**-0.98**	**(-1.51; -0.45) / P <0.001**	**Decreased more in the PPT-diet group than in the MED-diet group.**<br>+
BMI	Ben-Yacov, 2021	BMI ≤ 25	HbA1c (%)	6	DiD	0.000	(-0.15; 0.15) / P = n.s.	Null effect
**BMI**	**Ben-Yacov, 2021**	**25 < BMI ≤ 30**	**HbA1c (%)**	**6**	**DiD**	**-0.15**	**(-0.25; -0.05) / P <0.01**	**Decreased more in the PPT-diet group than in the MED-diet group.**<br>+

BMI	Ben-Yacov, 2021	30 < BMI ≤ 35	HbA1c (%)	6	DiD	-0.05	(-0.15; 0.05) / P = n.s.	Decreased more in the PPT-diet group than in the MED-diet group. +
BMI	Ben-Yacov, 2021	BMI > 35	HbA1c (%)	6	DiD	-0.055	(-0.19; 0.08) / P = n.s.	Decreased more in the PPT-diet group than in the MED-diet group. +
BMI	Ben-Yacov, 2021	BMI ≤ 25	OGTT (mmol/mol)	6	DiD	-0.89	(-3.003; 1.2155) / P = n.s.	Decreased more in the PPT-diet group than in the MED-diet group. +
BMI	Ben-Yacov, 2021	25 < BMI ≤ 30	OGTT (mmol/mol)	6	DiD	0.11	(-1.287; 1.507) / P = n.s.	Increased more in the PPT-diet group than in the MED-diet group. -
BMI	Ben-Yacov, 2021	30 < BMI ≤ 35	OGTT (mmol/mol)	6	DiD	0.105	(-0.759; 0.968) / P = n.s.	Increased more in the PPT-diet group than in the MED-diet group. -
BMI	Ben-Yacov, 2021	BMI > 35	OGTT (mmol/mol)	6	DiD	-0.646	(-2.068; 0.7755) / P = n.s.	Decreased more in the PPT-diet group than in the MED-diet group. +
BMI	Luo, 2022	Overweight (24≤ BMI＜ 28 kg/m^2)	0-h Glucose (mmol/l)	6	Within group comparison	MD: -0.35; TJD: -0.34; CD: -0.25	MD: (-0.47; -0.23); TJD: (-0.47; -0.21); CD: (-0.37; -0.12) / P = 0.37 (DiD)	Decreased more in the MD-group than in the CD-group. +
BMI	Luo, 2022	Obese (BMI ≥ 28 kg/m^2)	0-h Glucose (mmol/l)	6	Within group comparison	MD: -0.41; TJD: -0.35; CD: -0.3	MD: (-0.52; -0.3); TJD: (-0.46; -0.24); CD: (-0.41; -0.19) / P = 0.46 (DiD)	Decreased more in the MD-group than in the CD-group. +
BMI	Luo, 2022	Overweight (24≤ BMI＜ 28 kg/m^2)	0-h insulin (uIU/mL)	6	Within group comparison	MD: -3.2; TJD: -3.55; CD: -3.41	MD: (-4.38; -2.01); TJD: (-4.81; -2.28); CD: (-4.62; -2.21) / P = 0.84 (DiD)	Decreased more in the CD-group than in the MD-group. -
BMI	Luo, 2022	Obese (BMI ≥ 28 kg/m^2)	0-h insulin (uIU/mL)	6	Within group comparison	MD: -4.91; TJD: -4.58; CD: -4.19	MD: (-6.81; -3.01); TJD: (-6.49; -2.68); CD: (-6.05; -2.33) / P = 0.33 (DiD)	Decreased more in the MD-group than in the CD-group. +
BMI	Luo, 2022	Overweight (24≤ BMI＜ 28 kg/m^2)	AUC Glucose (mmol*h/L)	6	Within group comparison	MD: -0.72; TJD: 0.03; CD: -0.23	MD: (-1.34; -0.09); TJD: (-0.62; 0.69); CD: (-0.84; 0.39) / P = 0.92 (DiD)	Decreased more in the MD-group than in the CD-group. +
BMI	Luo, 2022	Obese (BMI ≥ 28 kg/m^2)	AUC Glucose (mmol*h/L)	6	Within group comparison	MD: -0.91; TJD: -0.23; CD: -0.45	MD: (-1.48; -0.33); TJD: (-0.8; 0.34); CD: (-1; 0.1) / P = 0.89 (DiD)	Decreased more in the MD-group than in the CD-group. +

BMI	Luo, 2022	Overweight (24≤ BMI< 28 kg/m^2)	AUC insulin (uIU*h/mL)	6	Within group comparison	MD: -18.2; TJD: -20.7; CD: -10.6	MD: (-39.7; 3.39); TJD: (-43.5; 2.21); CD: (-31.8; 10.6) / P = 0.11 (DiD)	Decreased more in the MD-group than in the CD-group. +
BMI	Luo, 2022	Obese (BMI ≥ 28 kg/m^2)	AUC insulin (uIU*h/mL)	6	Within group comparison	MD: -31.2; TJD: -26.5; CD: -23.0	MD: (-52.6; -9.8); TJD: (-47.8; -5.17); CD: (-43.2; -2.84) / P = 0.46 (DiD)	Decreased more in the MD-group than in the CD-group. +
BMI	Luo, 2022	Overweight (24≤ BMI< 28 kg/m^2)	Mastuda Index (N/A)	6	Within group comparison	MD: 4.94; TJD: 3.6; CD: 4.59	MD: (2.92; 6.96); TJD: (1.44; 5.77); CD: (2.63; 6.55) / P = 0.80 (DiD)	Decreased more in the MD-group than in the CD-group. +
BMI	Luo, 2022	Obese (BMI ≥ 28 kg/m^2)	Mastuda Index (N/A)	6	Within group comparison	MD: 5.31; TJD: 3.94; CD: 4.05	MD: (3.45; 7.17); TJD: (2.11; 5.77); CD: (2.33; 5.78) / P = 0.79 (DiD)	Increased more in the MD-group than in the CD-group. +
**BMI**	**Luo, 2022**	**Overweight (24≤ BMI< 28 kg/m^2)**	**Mean Glucose (mmol/l)**	**6**	**Within group comparison**	**MD: -0.32; TJD: -0.45; CD: -0.66**	**MD: (-0.47; -0.16); TJD: (-0.62; -0.29); CD: (-0.81; -0.5) / P = 0.015 (DiD)**	**Decreased more in the CD-group than in the MD-group. -**
BMI	Luo, 2022	Obese (BMI ≥ 28 kg/m^2)	Mean Glucose (mmol/l)	6	Within group comparison	MD: -0.42; TJD: -0.38; CD: -0.67	MD: (-0.57; -0.27); TJD: (-0.53; -0.24); CD: (-0.82; -0.53) / P = 0.70 (DiD)	Decreased more in the CD-group than in the MD-group. -
BMI	Luo, 2022	Overweight (24≤ BMI< 28 kg/m^2)	TAR (%)	6	Within group comparison	MD: -0.26; TJD: -0.37; CD: -0.46	MD: (-0.42; -0.11); TJD: (-0.54; -0.2); CD: (-0.62; -0.3) / P = 0.072 (DiD)	Decreased more in the CD-group than in the MD-group. -
BMI	Luo, 2022	Obese (BMI ≥ 28 kg/m^2)	TAR (%)	6	Within group comparison	MD: -0.21; TJD: -0.26; CD: -0.41	MD: (-0.37; -0.04); TJD: (-0.42; -0.1); CD: (-0.57; -0.25) / P = 0.94 (DiD)	Decreased more in the CD-group than in the MD-group. -
**BMI**	**Luo, 2022**	**Overweight (24≤ BMI< 28 kg/m^2)**	**TBR, <3.9 mmol/l (%)**	**6**	**Within group comparison**	**MD: 0.16; TJD: 0.52; CD: 0.93**	**MD: (-0.32; 0.63); TJD: (0.02; 1.02); CD: (0.46; 1.4) / P = 0.045 (DiD)**	**Increased less in the MD-group than in the CD-group. +**
BMI	Luo, 2022	Obese (BMI ≥ 28 kg/m^2)	TBR, <3.9 mmol/l (%)	6	Within group comparison	MD: 0.4; TJD: 0.3; CD: 1.14	MD: (-0.06; 0.85); TJD: (-0.15; 0.74); CD: (0.7, 1.58) / P = 0.30 (DiD)	Increased less in the MD-group than in the CD-group. +
**BMI**	**Luo, 2022**	**Overweight (24≤ BMI< 28 kg/m^2)**	**TBR level 1, 3-3.9 mmol/l (%)**	**6**	**Within group comparison**	**MD: 0.18; TJD: 0.54; CD: 0.99**	**MD: (-0.26; 0.62); TJD: (0.07; 1); CD: (0.55; 1.42) / P = 0.033 (DiD)**	**Increased less in the MD-group than in the CD-group. +**
BMI	Luo, 2022	Obese (BMI ≥ 28 kg/m^2)	TBR level 1, 3-3.9 mmol/l (%)	6	Within group comparison	MD: 0.4; TJD: 0.34; CD: 1.16	MD: (-0.02; 0.82); TJD: (-0.08; 0.75); CD: (0.76; 1.57) / P = 0.23 (DiD)	Increased less in the MD-group than in the CD-group. +

BMI	Luo, 2022	Overweight (24≤ BMI< 28 kg/m^2)	TBR level 2, <3 mmol/l (%)	6	Within group comparison	MD: -0.04; TJD: 0.08; CD: 0.02	MD: (-0.27; 0.2); TJD: (-0.18; 0.33); CD: (-0.22; 0.26) / P = 0.69 (DiD)	Decreased in the MD-group and increased in the CD-group. +
BMI	Luo, 2022	Obese (BMI ≥ 28 kg/m^2)	TBR level 2, <3 mmol/l (%)	6	Within group comparison	MD: 0.04; TJD: -0.01; CD: 0.11	MD: (-0.19; 0.28); TJD: (-0.24; 0.22); CD: (-0.11; 0.34) / P = 0.99 (DiD)	Increased less in the MD-group than in the CD-group. +
BMI	Luo, 2022	Overweight (24≤ BMI< 28 kg/m^2)	Nighttime SD (mmol/l)	6	Within group comparison	MD: -0.02; TJD: -0.1; CD: -0.1	MD: (-0.09; 0.05); TJD: (-0.17; -0.03); CD: (-0.17; -0.03) / P = 0.22 (DiD)	Decreased more in the CD-group than in the MD-group. -
BMI	Luo, 2022	Obese (BMI ≥ 28 kg/m^2)	Nighttime SD (mmol/l)	6	Within group comparison	MD: -0.01; TJD: -0.14; CD: -0.11	MD: (-0.08; 0.05); TJD: (-0.21; -0.08); CD: (-0.17; -0.04) / P = 0.49 (DiD)	Decreased more in the CD-group than in the MD-group. -
BMI	Luo, 2022	Obese (BMI ≥ 28 kg/m^2)	Weight (kg)	6	Within group comparison	MD: -5.82; TJD: -5.25; CD: -5.4	MD: (-6.59; -5.05); TJD: (-6; -4.5); CD: (-6.14; -4.65) / P = 0.24 (DiD)	Decreased more in the MD-group than in the CD-group. +
BMI	Luo, 2022	Overweight (24≤ BMI< 28 kg/m^2)	Weight (kg)	6	Within group comparison	MD: -4.87; TJD: -4.86; CD: -4.64	MD: (-5.58; -4.16); TJD: (-5.59; -4.13); CD: (-5.36; -3.92) / P = 0.44 (DiD)	Decreased more in the MD-group than in the CD-group. +
**Top 80% compliance**	**Ben-Yacov, 2021**	**Top 80% compliance**	**Glucose level time above 140 mg/dL (hour/day)**	**6**	**DiD**	**-1.135**	**(-1.5; -0.77) / P < 0.001**	**Decreased more in the PPT-diet group than in the MED-diet group. +**
**Top 80% compliance**	**Ben-Yacov, 2021**	**Top 80% compliance**	**HbA1c (%)**	**6**	**DiD**	**-0.105**	**(-0.17; -0.04) / P <0.01**	**Decreased more in the PPT-diet group than in the MED-diet group. +**
Top 80% compliance	Ben-Yacov, 2021	Top 80% compliance	OGTT (mmol/mol)	6	DiD	-0.349	(-0.107; 0.368) / P = n.s.	Decreased more in the PPT-diet group than in the MED-diet group. +

Notes: Statistically significant results are written in **bold**. In Luo et al, 2022 the main comparison is between MD as intervention group and CD as control group.
† Signs with + or – indicated if the intervention had better or worse effects respectively in the intervention in comparison with the control group.
‡ Results shown are of the main population of the study which included diabetic and prediabetic patients.
Abbreviations: CD: control diet; CI: confidence interval; DiD: difference between means from baseline to follow-up; MD: mediterranean diet; N/A: not available; n.s.: not statistically significant; OGTT: oral glucose tolerance test; TAR: time above range; TBR: time below range; TJD: Traditional Jiangnan diet.

Appendix S28: Leave-one-out forest plots

Weight (kg)

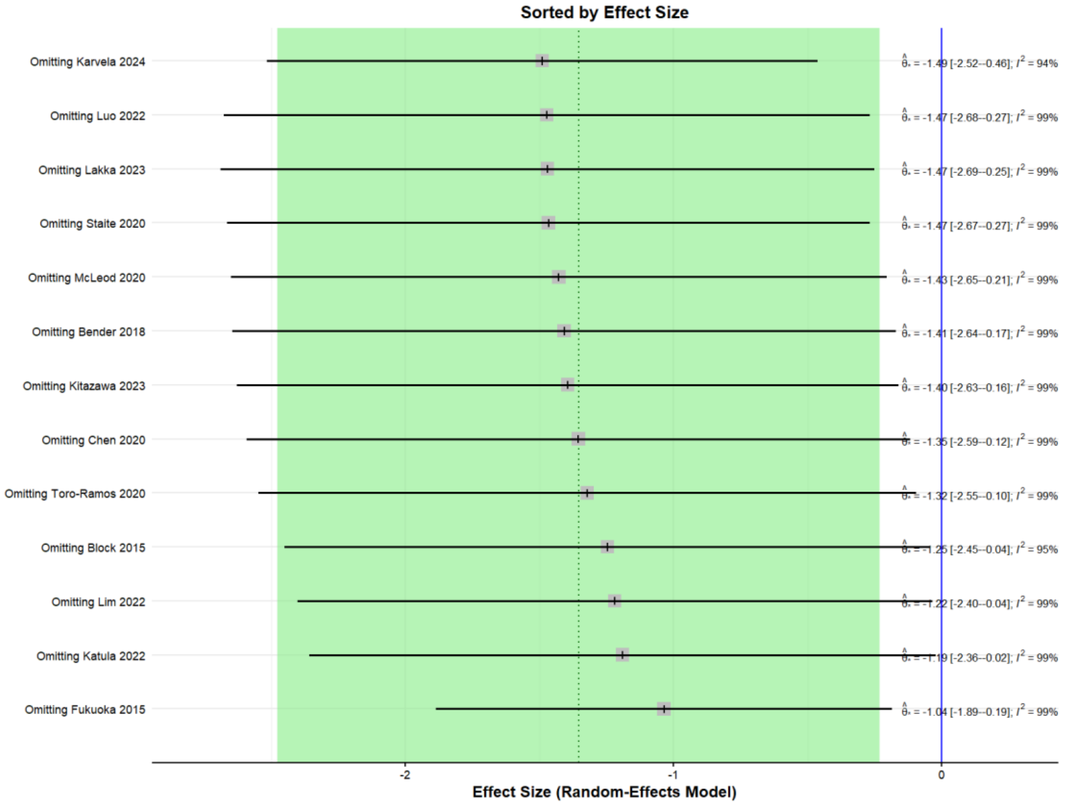

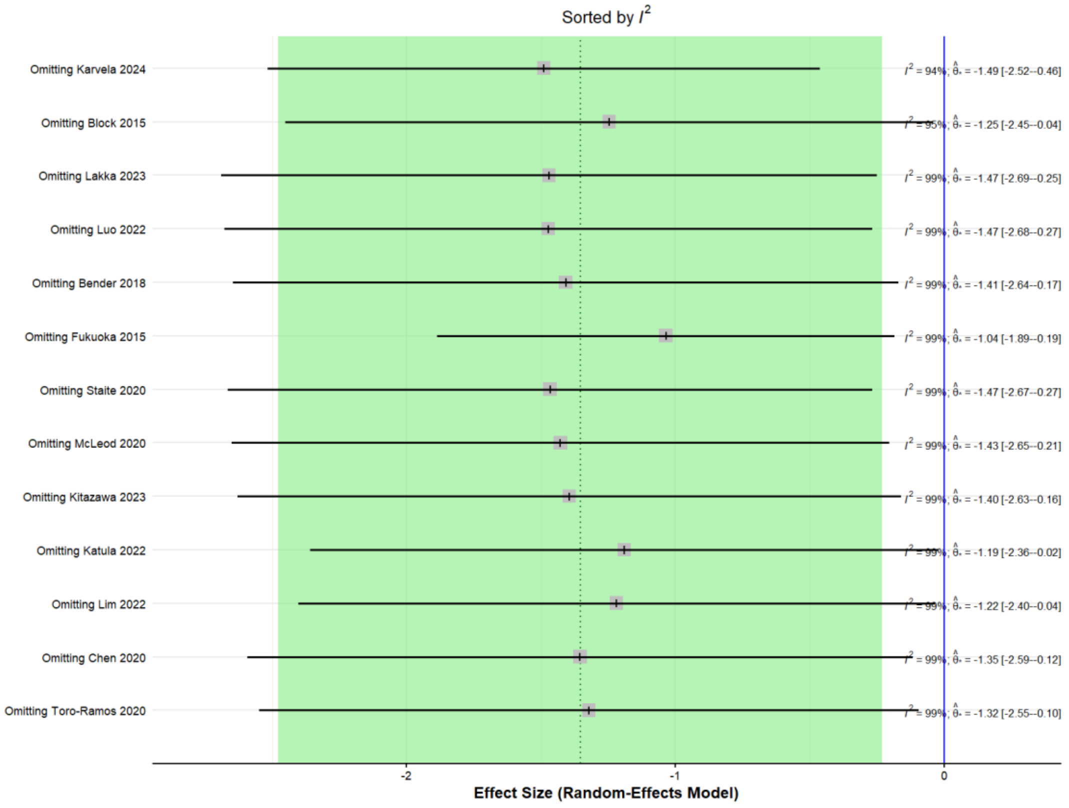

BMI

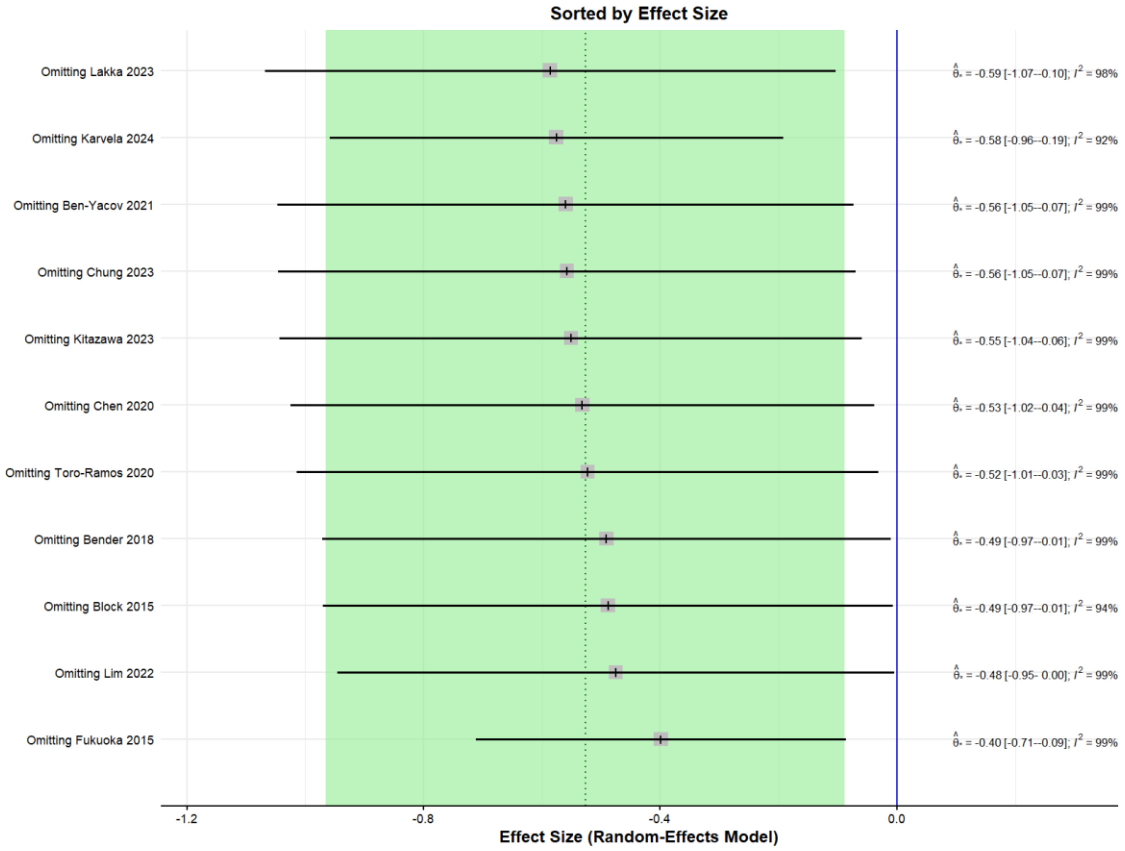
Sorted by Effect Size
Omitting Lakka 2023
Omitting Karvela 2024
Omitting Ben-Yacov 2021
Omitting Chung 2023
Omitting Kitazawa 2023
Omitting Chen 2020
Omitting Toro-Ramos 2020
Omitting Bender 2018
Omitting Block 2015
Omitting Lim 2022
Omitting Fukuoka 2015
θ̂ = -0.59 [-1.07--0.10]; I2 = 98%
θ̂ = -0.58 [-0.96--0.19]; I2 = 92%
θ̂ = -0.56 [-1.05--0.07]; I2 = 99%
θ̂ = -0.56 [-1.05--0.07]; I2 = 99%
θ̂ = -0.55 [-1.04--0.06]; I2 = 99%
θ̂ = -0.53 [-1.02--0.04]; I2 = 99%
θ̂ = -0.52 [-1.01--0.03]; I2 = 99%
θ̂ = -0.49 [-0.97--0.01]; I2 = 99%
θ̂ = -0.49 [-0.97--0.01]; I2 = 94%
θ̂ = -0.48 [-0.95- 0.00]; I2 = 99%
θ̂ = -0.40 [-0.71--0.09]; I2 = 99%
-1.2
-0.8
-0.4
0.0
Effect Size (Random-Effects Model)

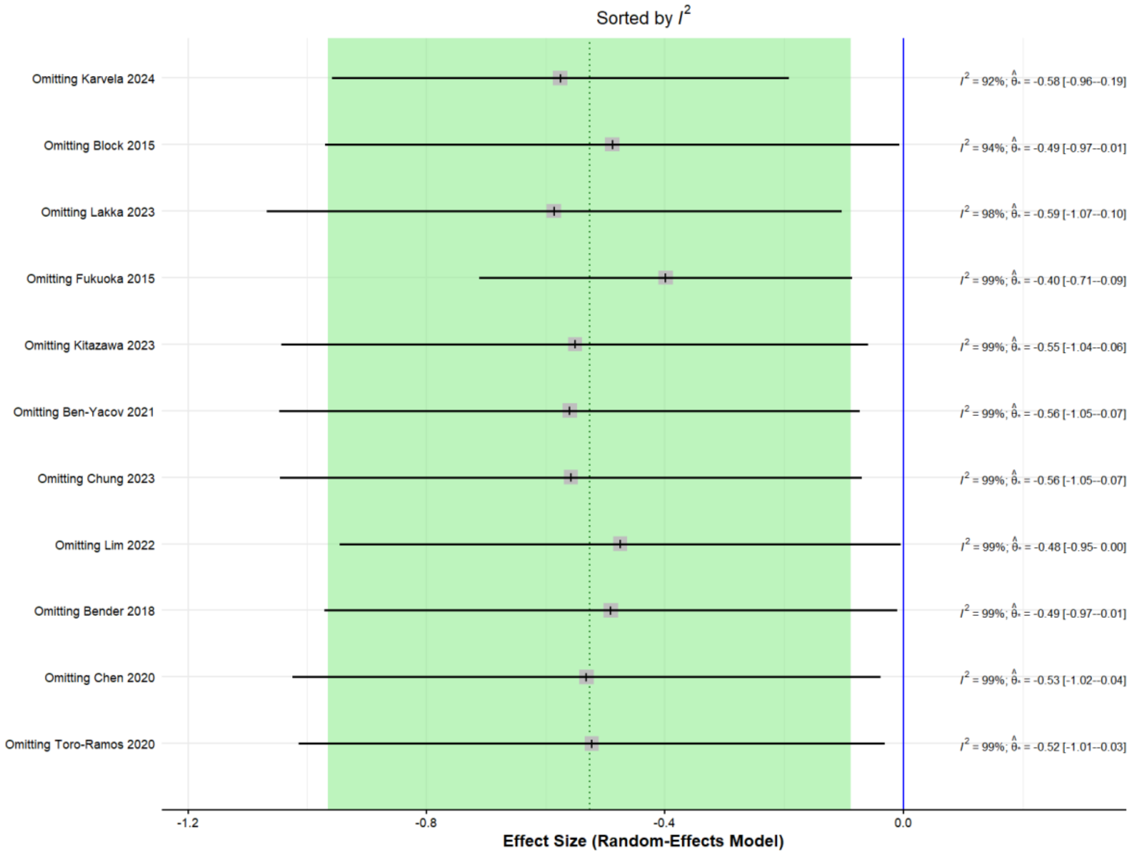
Sorted by I2
Omitting Karvela 2024
Omitting Block 2015
Omitting Lakka 2023
Omitting Fukuoka 2015
Omitting Kitazawa 2023
Omitting Ben-Yacov 2021
Omitting Chung 2023
Omitting Lim 2022
Omitting Bender 2018
Omitting Chen 2020
Omitting Toro-Ramos 2020
I2 = 92%; θ̂ = -0.58 [-0.96--0.19]
I2 = 94%; θ̂ = -0.49 [-0.97--0.01]
I2 = 98%; θ̂ = -0.59 [-1.07--0.10]
I2 = 99%; θ̂ = -0.40 [-0.71--0.09]
I2 = 99%; θ̂ = -0.55 [-1.04--0.06]
I2 = 99%; θ̂ = -0.56 [-1.05--0.07]
I2 = 99%; θ̂ = -0.56 [-1.05--0.07]
I2 = 99%; θ̂ = -0.48 [-0.95- 0.00]
I2 = 99%; θ̂ = -0.49 [-0.97--0.01]
I2 = 99%; θ̂ = -0.53 [-1.02--0.04]
I2 = 99%; θ̂ = -0.52 [-1.01--0.03]
-1.2
-0.8
-0.4
0.0
Effect Size (Random-Effects Model)

HbA1c (%)

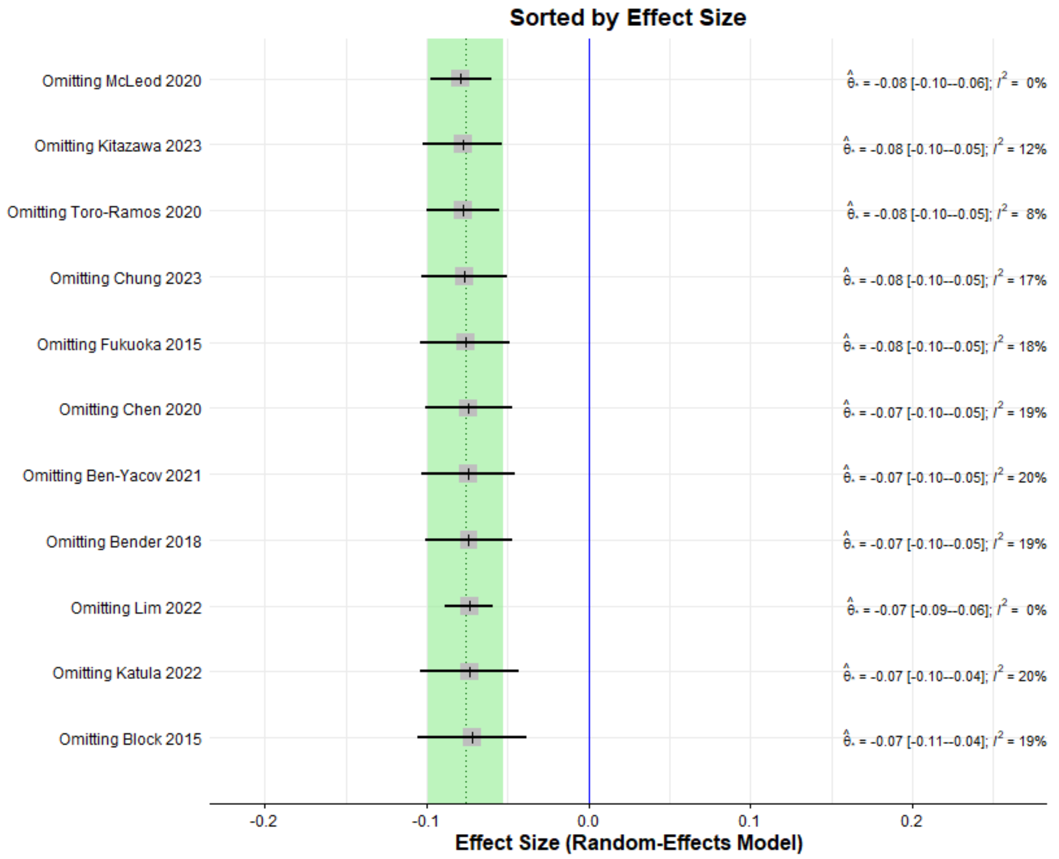
Sorted by Effect Size
Omitting McLeod 2020
Omitting Kitazawa 2023
Omitting Toro-Ramos 2020
Omitting Chung 2023
Omitting Fukuoka 2015
Omitting Chen 2020
Omitting Ben-Yacov 2021
Omitting Bender 2018
Omitting Lim 2022
Omitting Katula 2022
Omitting Block 2015
θ̂. = -0.08 [-0.10--0.06]; I² = 0%
θ̂. = -0.08 [-0.10--0.05]; I² = 12%
θ̂. = -0.08 [-0.10--0.05]; I² = 8%
θ̂. = -0.08 [-0.10--0.05]; I² = 17%
θ̂. = -0.08 [-0.10--0.05]; I² = 18%
θ̂. = -0.07 [-0.10--0.05]; I² = 19%
θ̂. = -0.07 [-0.10--0.05]; I² = 20%
θ̂. = -0.07 [-0.10--0.05]; I² = 19%
θ̂. = -0.07 [-0.09--0.06]; I² = 0%
θ̂. = -0.07 [-0.10--0.04]; I² = 20%
θ̂. = -0.07 [-0.11--0.04]; I² = 19%
-0.2
-0.1
0.0
0.1
0.2
Effect Size (Random-Effects Model)

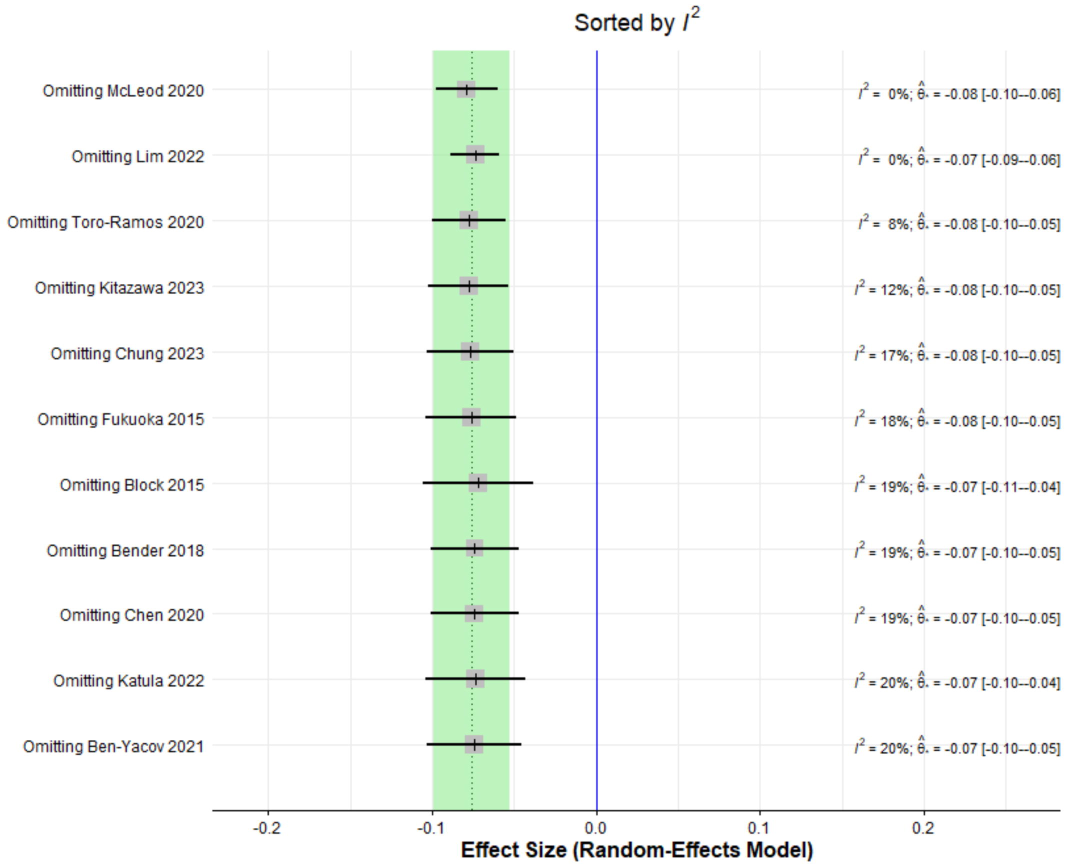
Sorted by I²
Omitting McLeod 2020
Omitting Lim 2022
Omitting Toro-Ramos 2020
Omitting Kitazawa 2023
Omitting Chung 2023
Omitting Fukuoka 2015
Omitting Block 2015
Omitting Bender 2018
Omitting Chen 2020
Omitting Katula 2022
Omitting Ben-Yacov 2021
I² = 0%; θ̂. = -0.08 [-0.10--0.06]
I² = 0%; θ̂. = -0.07 [-0.09--0.06]
I² = 8%; θ̂. = -0.08 [-0.10--0.05]
I² = 12%; θ̂. = -0.08 [-0.10--0.05]
I² = 17%; θ̂. = -0.08 [-0.10--0.05]
I² = 18%; θ̂. = -0.08 [-0.10--0.05]
I² = 19%; θ̂. = -0.07 [-0.11--0.04]
I² = 19%; θ̂. = -0.07 [-0.10--0.05]
I² = 19%; θ̂. = -0.07 [-0.10--0.05]
I² = 20%; θ̂. = -0.07 [-0.10--0.04]
I² = 20%; θ̂. = -0.07 [-0.10--0.05]
-0.2
-0.1
0.0
0.1
0.2
Effect Size (Random-Effects Model)

SBP

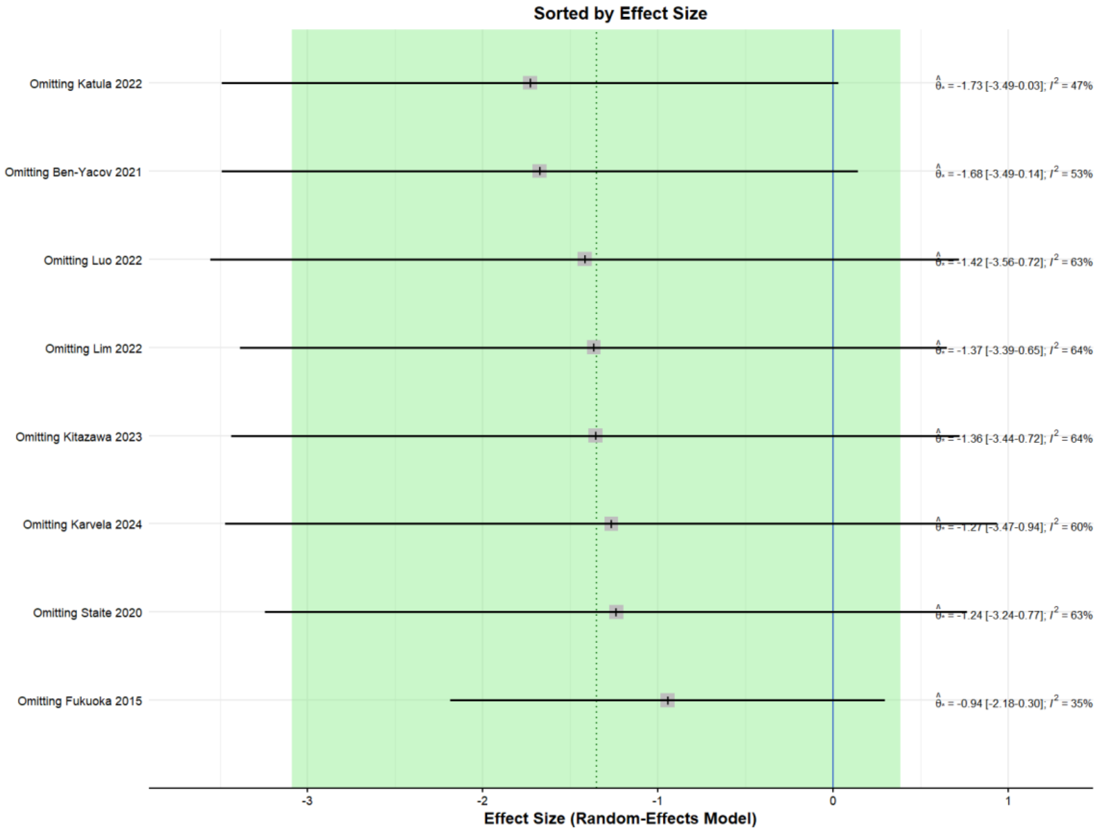

Sorted by Effect Size
Omitting Katula 2022
Omitting Ben-Yacov 2021
Omitting Luo 2022
Omitting Lim 2022
Omitting Kitazawa 2023
Omitting Karvela 2024
Omitting Staite 2020
Omitting Fukuoka 2015
θ̂ = -1.73 [-3.49-0.03]; I² = 47%
θ̂ = -1.68 [-3.49-0.14]; I² = 53%
θ̂ = -1.42 [-3.56-0.72]; I² = 63%
θ̂ = -1.37 [-3.39-0.65]; I² = 64%
θ̂ = -1.36 [-3.44-0.72]; I² = 64%
θ̂ = -1.27 [-3.47-0.94]; I² = 60%
θ̂ = -1.24 [-3.24-0.77]; I² = 63%
θ̂ = -0.94 [-2.18-0.30]; I² = 35%
-3
-2
-1
0
1
Effect Size (Random-Effects Model)

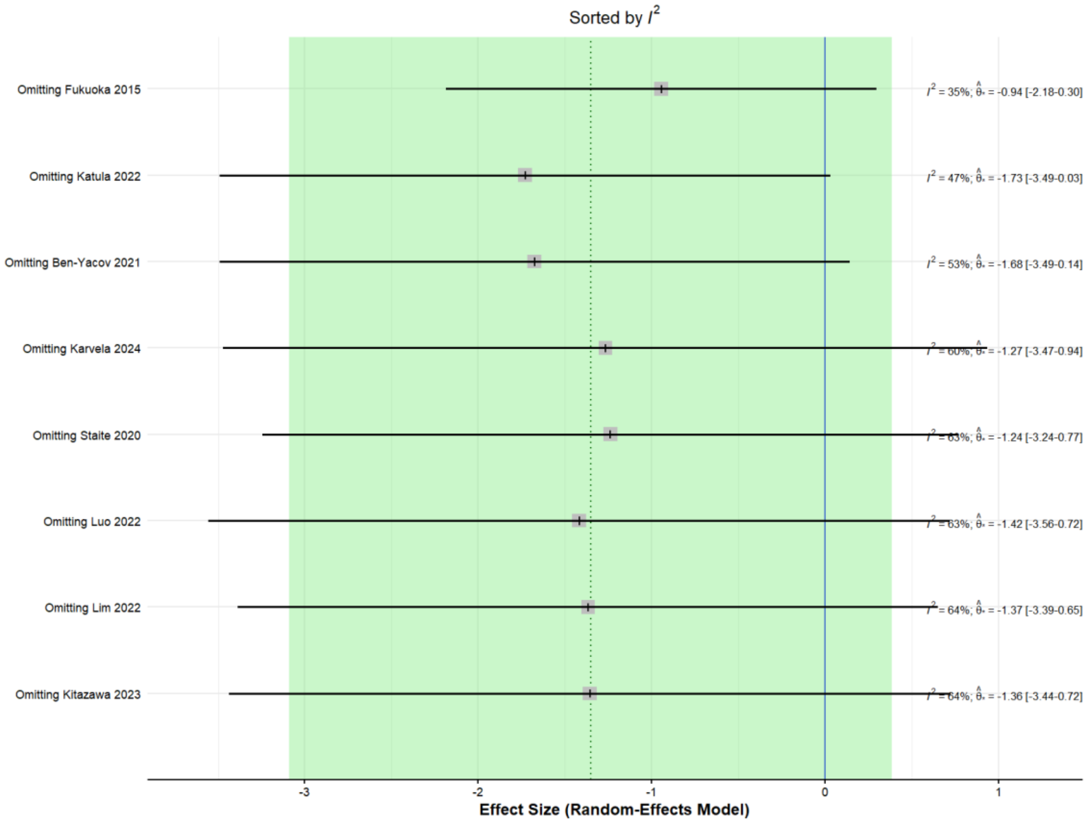

Sorted by I²
Omitting Fukuoka 2015
Omitting Katula 2022
Omitting Ben-Yacov 2021
Omitting Karvela 2024
Omitting Staite 2020
Omitting Luo 2022
Omitting Lim 2022
Omitting Kitazawa 2023
I² = 35%; θ̂ = -0.94 [-2.18-0.30]
I² = 47%; θ̂ = -1.73 [-3.49-0.03]
I² = 53%; θ̂ = -1.68 [-3.49-0.14]
I² = 60%; θ̂ = -1.27 [-3.47-0.94]
I² = 63%; θ̂ = -1.24 [-3.24-0.77]
I² = 63%; θ̂ = -1.42 [-3.56-0.72]
I² = 64%; θ̂ = -1.37 [-3.39-0.65]
I² = 64%; θ̂ = -1.36 [-3.44-0.72]
-3
-2
-1
0
1
Effect Size (Random-Effects Model)

DBP

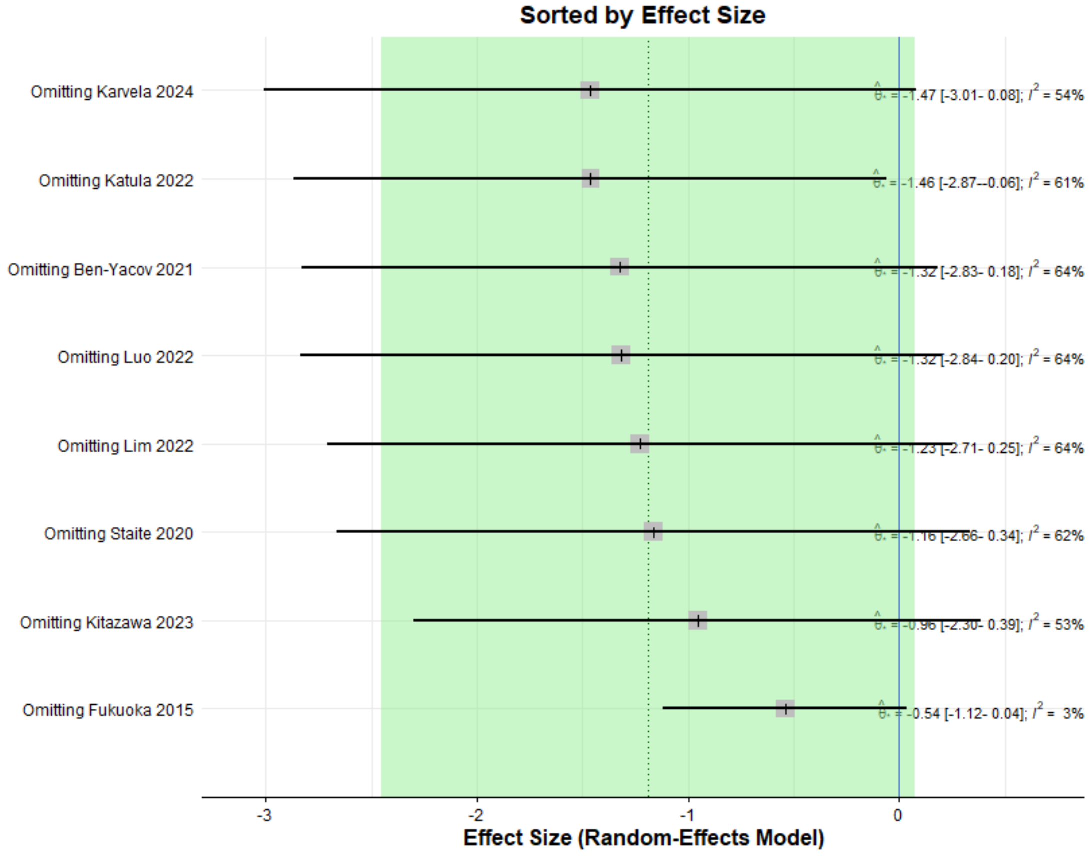

Sorted by Effect Size
Omitting Karvela 2024
Omitting Katula 2022
Omitting Ben-Yacov 2021
Omitting Luo 2022
Omitting Lim 2022
Omitting Staite 2020
Omitting Kitazawa 2023
Omitting Fukuoka 2015
θ̂ = -1.47 [-3.01- 0.08]; I² = 54%
θ̂ = -1.46 [-2.87--0.06]; I² = 61%
θ̂ = -1.32 [-2.83- 0.18]; I² = 64%
θ̂ = -1.32 [-2.84- 0.20]; I² = 64%
θ̂ = -1.23 [-2.71- 0.25]; I² = 64%
θ̂ = -1.16 [-2.66- 0.34]; I² = 62%
θ̂ = -0.96 [-2.30- 0.39]; I² = 53%
θ̂ = -0.54 [-1.12- 0.04]; I² = 3%
-3
-2
-1
0
Effect Size (Random-Effects Model)

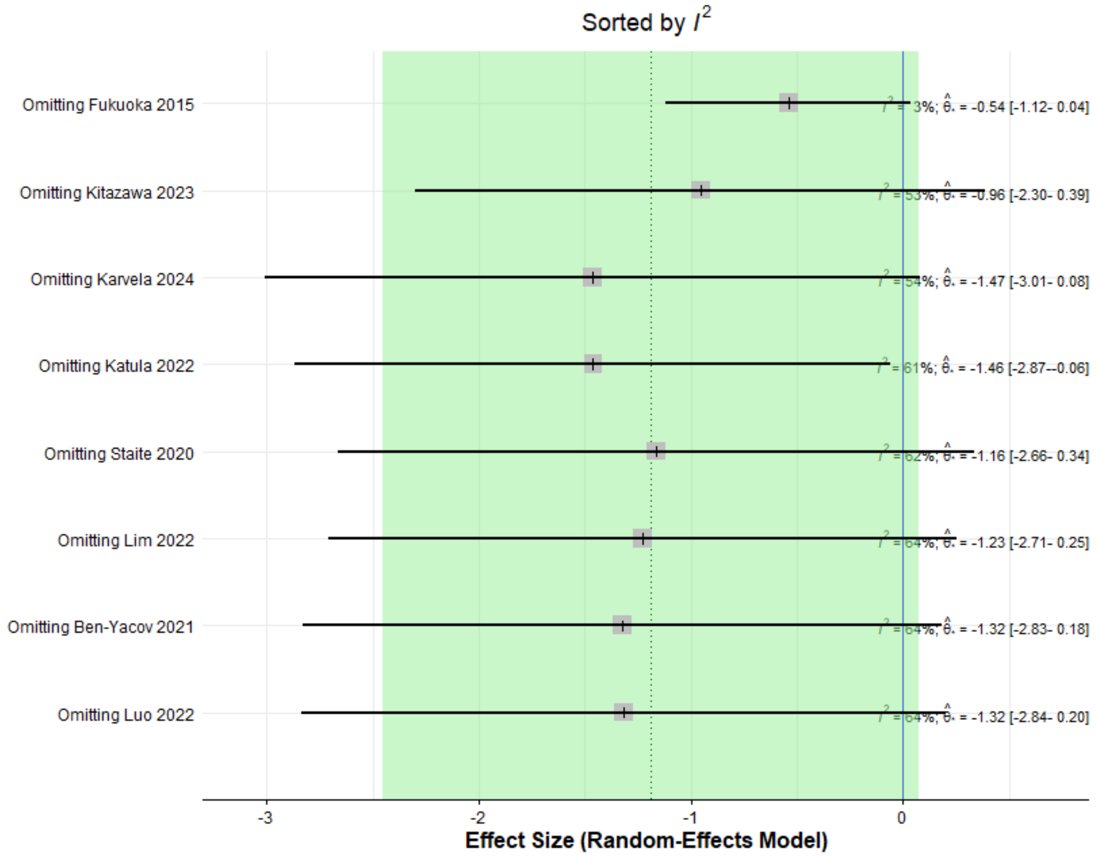

Sorted by I²
Omitting Fukuoka 2015
Omitting Kitazawa 2023
Omitting Karvela 2024
Omitting Katula 2022
Omitting Staite 2020
Omitting Lim 2022
Omitting Ben-Yacov 2021
Omitting Luo 2022
I² = 3%; θ̂ = -0.54 [-1.12- 0.04]
I² = 53%; θ̂ = -0.96 [-2.30- 0.39]
I² = 54%; θ̂ = -1.47 [-3.01- 0.08]
I² = 61%; θ̂ = -1.46 [-2.87--0.06]
I² = 62%; θ̂ = -1.16 [-2.66- 0.34]
I² = 64%; θ̂ = -1.23 [-2.71- 0.25]
I² = 64%; θ̂ = -1.32 [-2.83- 0.18]
I² = 64%; θ̂ = -1.32 [-2.84- 0.20]
-3
-2
-1
0
Effect Size (Random-Effects Model)

FPG

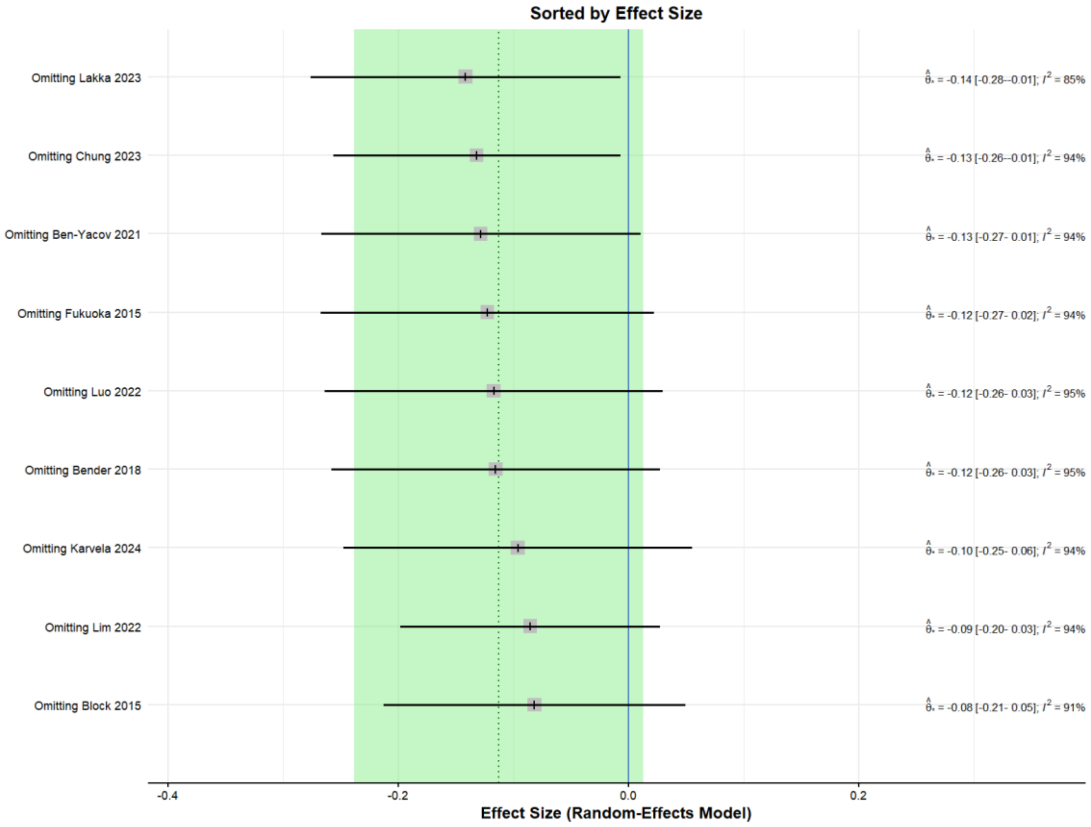

Sorted by Effect Size
Omitting Lakka 2023
Omitting Chung 2023
Omitting Ben-Yacov 2021
Omitting Fukuoka 2015
Omitting Luo 2022
Omitting Bender 2018
Omitting Karvela 2024
Omitting Lim 2022
Omitting Block 2015
θ̂* = -0.14 [-0.28--0.01]; I² = 85%
θ̂* = -0.13 [-0.26--0.01]; I² = 94%
θ̂* = -0.13 [-0.27- 0.01]; I² = 94%
θ̂* = -0.12 [-0.27- 0.02]; I² = 94%
θ̂* = -0.12 [-0.26- 0.03]; I² = 95%
θ̂* = -0.12 [-0.26- 0.03]; I² = 95%
θ̂* = -0.10 [-0.25- 0.06]; I² = 94%
θ̂* = -0.09 [-0.20- 0.03]; I² = 94%
θ̂* = -0.08 [-0.21- 0.05]; I² = 91%
-0.4
-0.2
0.0
0.2
Effect Size (Random-Effects Model)

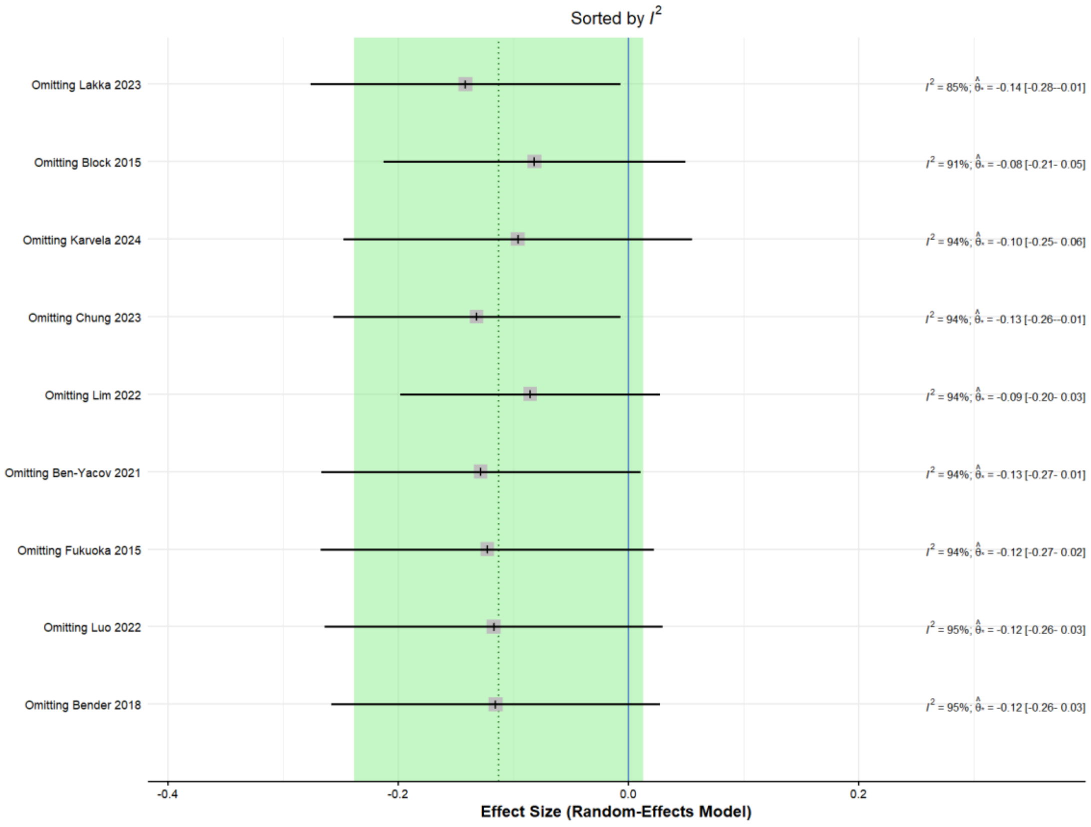

Sorted by I²
Omitting Lakka 2023
Omitting Block 2015
Omitting Karvela 2024
Omitting Chung 2023
Omitting Lim 2022
Omitting Ben-Yacov 2021
Omitting Fukuoka 2015
Omitting Luo 2022
Omitting Bender 2018
I² = 85%; θ̂* = -0.14 [-0.28--0.01]
I² = 91%; θ̂* = -0.08 [-0.21- 0.05]
I² = 94%; θ̂* = -0.10 [-0.25- 0.06]
I² = 94%; θ̂* = -0.13 [-0.26--0.01]
I² = 94%; θ̂* = -0.09 [-0.20- 0.03]
I² = 94%; θ̂* = -0.13 [-0.27- 0.01]
I² = 94%; θ̂* = -0.12 [-0.27- 0.02]
I² = 95%; θ̂* = -0.12 [-0.26- 0.03]
I² = 95%; θ̂* = -0.12 [-0.26- 0.03]
-0.4
-0.2
0.0
0.2
Effect Size (Random-Effects Model)

HDL

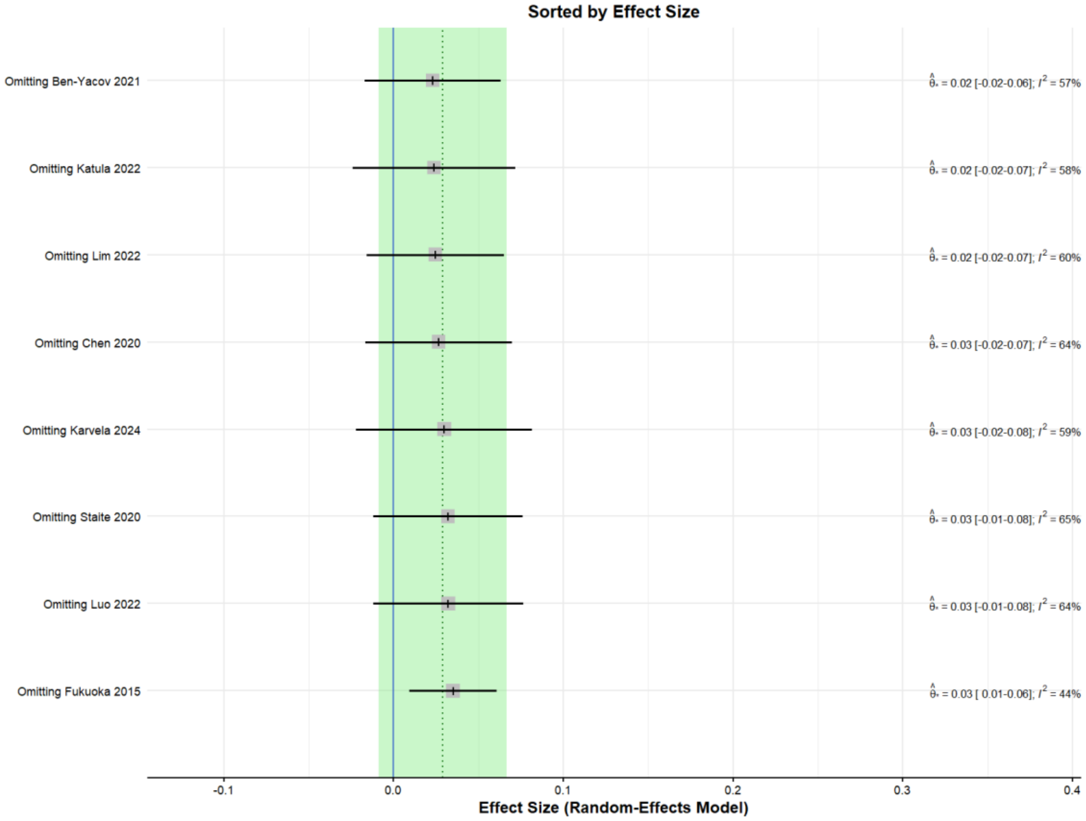
Sorted by Effect Size
Omitting Ben-Yacov 2021
Omitting Katula 2022
Omitting Lim 2022
Omitting Chen 2020
Omitting Karvela 2024
Omitting Staite 2020
Omitting Luo 2022
Omitting Fukuoka 2015
θ̂* = 0.02 [-0.02-0.06]; I² = 57%
θ̂* = 0.02 [-0.02-0.07]; I² = 58%
θ̂* = 0.02 [-0.02-0.07]; I² = 60%
θ̂* = 0.03 [-0.02-0.07]; I² = 64%
θ̂* = 0.03 [-0.02-0.08]; I² = 59%
θ̂* = 0.03 [-0.01-0.08]; I² = 65%
θ̂* = 0.03 [-0.01-0.08]; I² = 64%
θ̂* = 0.03 [0.01-0.06]; I² = 44%
-0.1
0.0
0.1
0.2
0.3
0.4
Effect Size (Random-Effects Model)

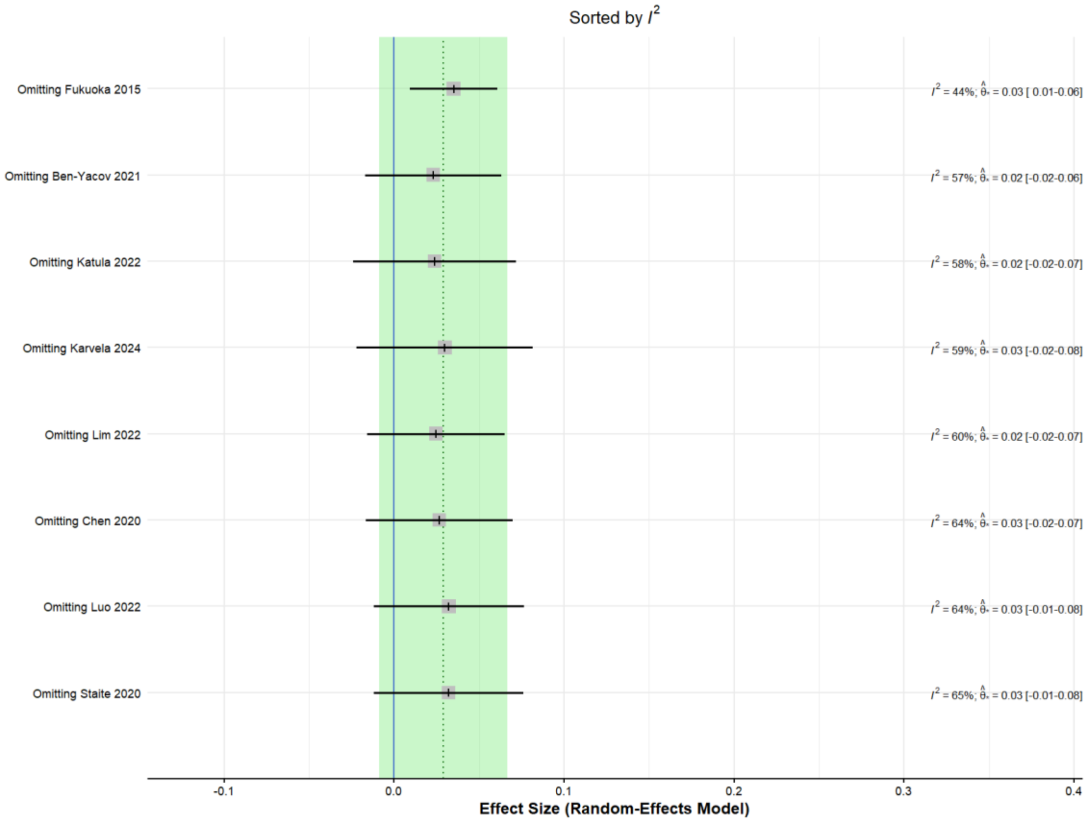
Sorted by I²
Omitting Fukuoka 2015
Omitting Ben-Yacov 2021
Omitting Katula 2022
Omitting Karvela 2024
Omitting Lim 2022
Omitting Chen 2020
Omitting Luo 2022
Omitting Staite 2020
I² = 44%; θ̂* = 0.03 [0.01-0.06]
I² = 57%; θ̂* = 0.02 [-0.02-0.06]
I² = 58%; θ̂* = 0.02 [-0.02-0.07]
I² = 59%; θ̂* = 0.03 [-0.02-0.08]
I² = 60%; θ̂* = 0.02 [-0.02-0.07]
I² = 64%; θ̂* = 0.03 [-0.02-0.07]
I² = 64%; θ̂* = 0.03 [-0.01-0.08]
I² = 65%; θ̂* = 0.03 [-0.01-0.08]
-0.1
0.0
0.1
0.2
0.3
0.4
Effect Size (Random-Effects Model)

Total Cholesterol

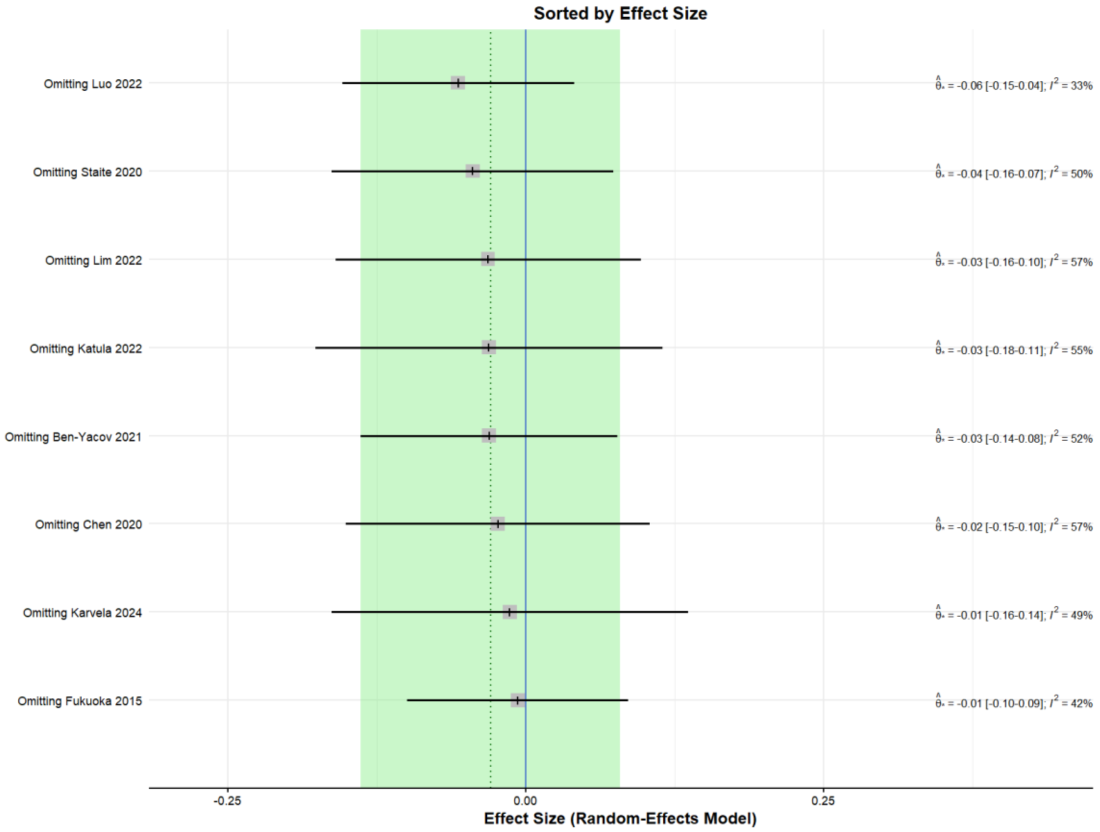

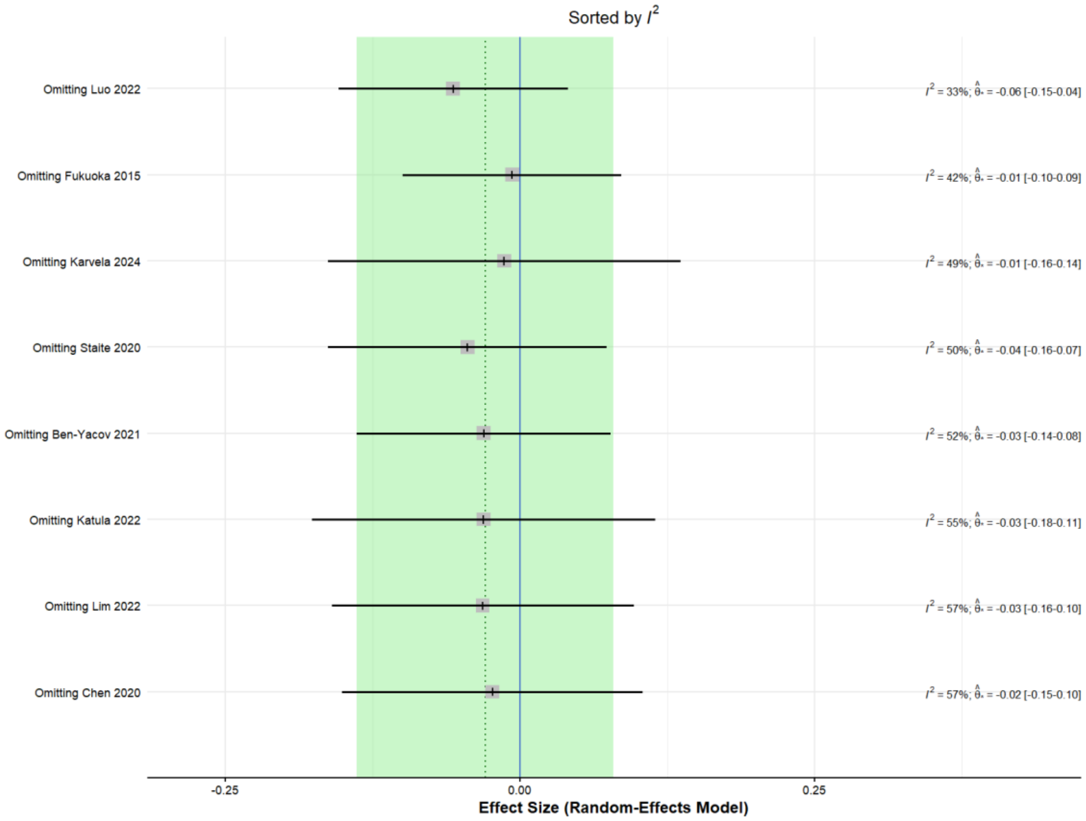

Triglycerides

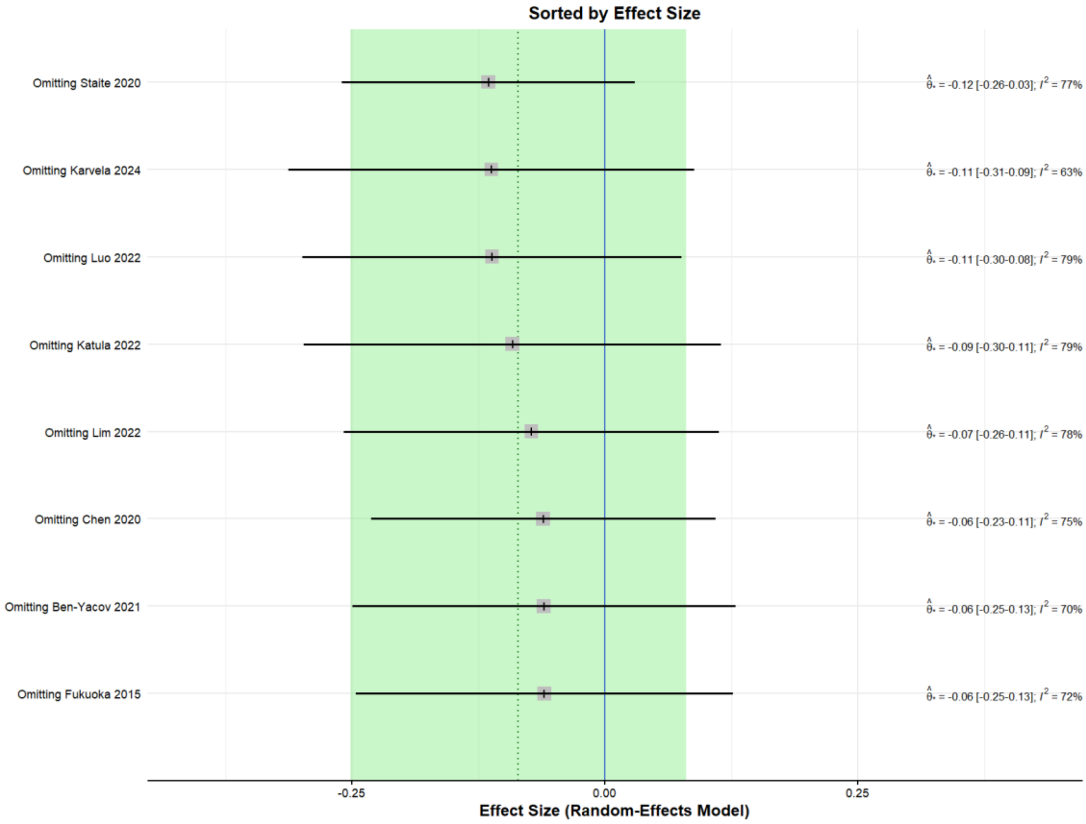

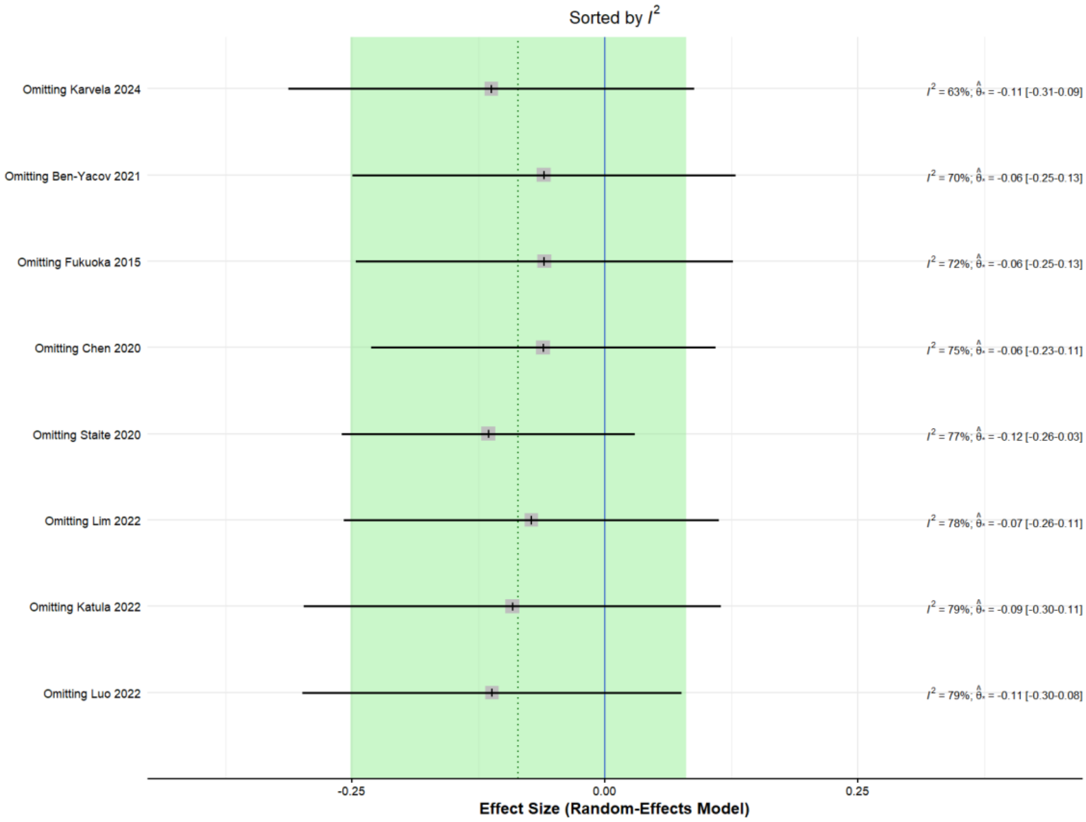

HbA1c (mmol/mol)

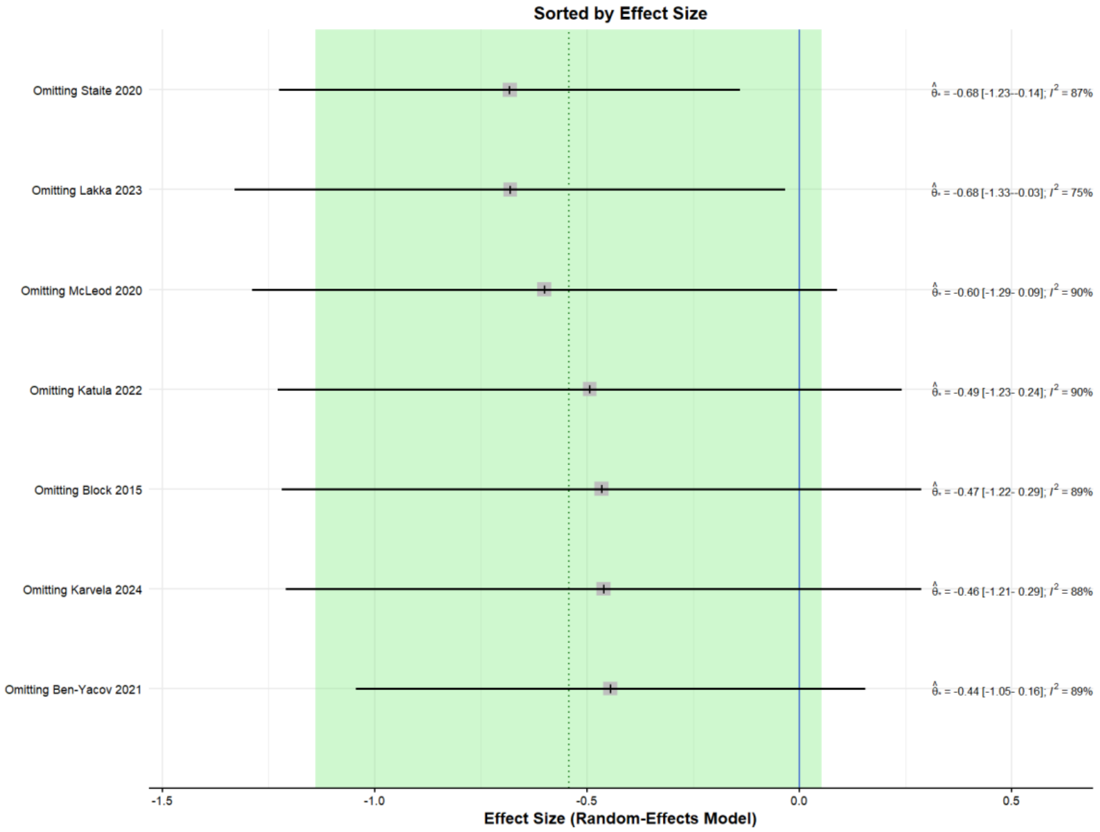

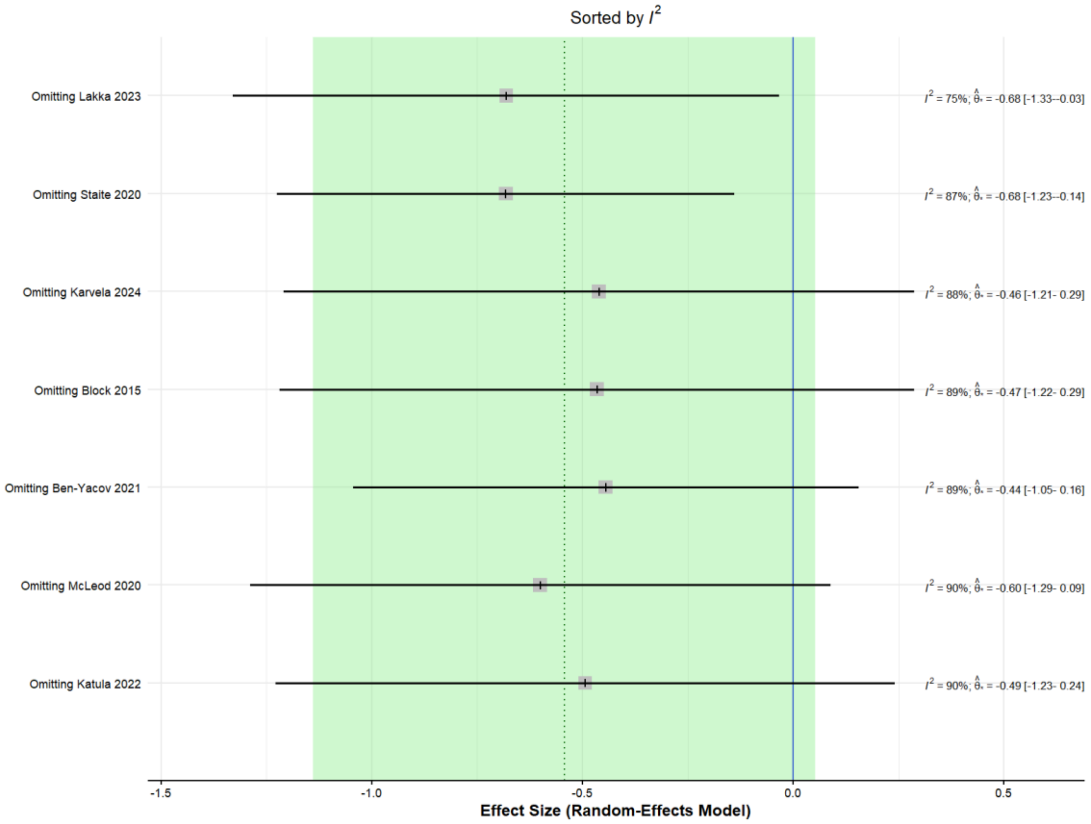

Waist circumference

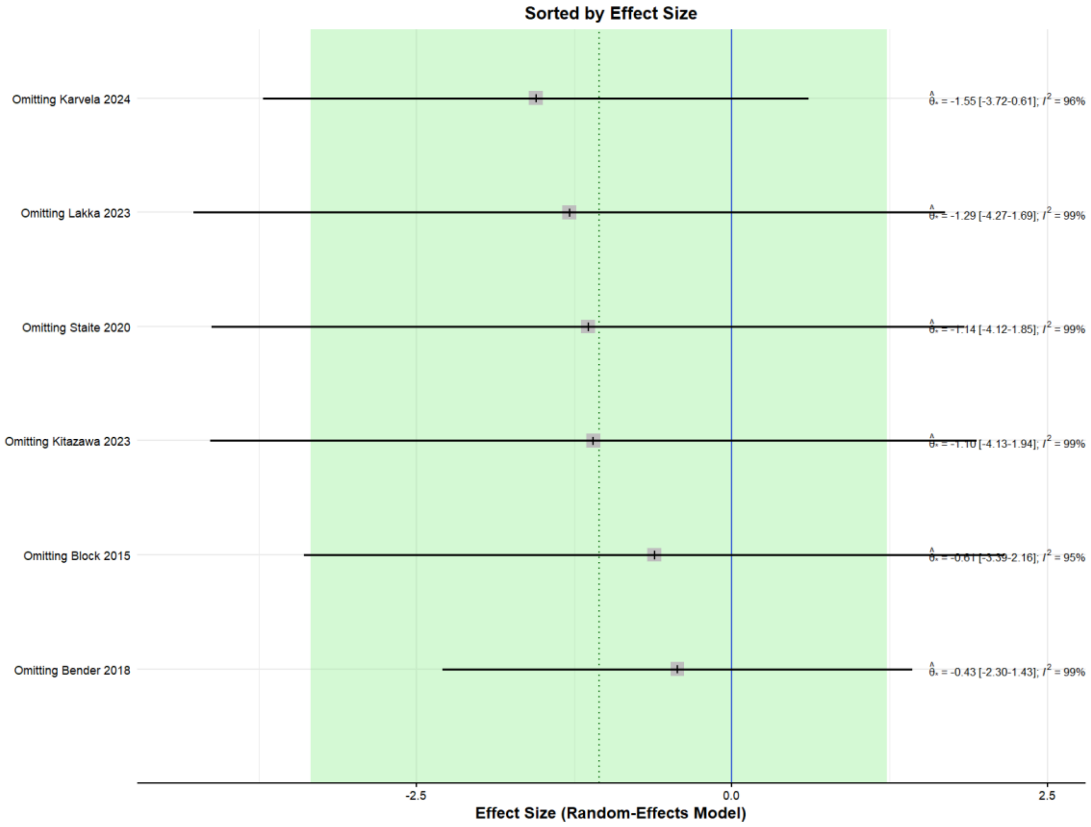

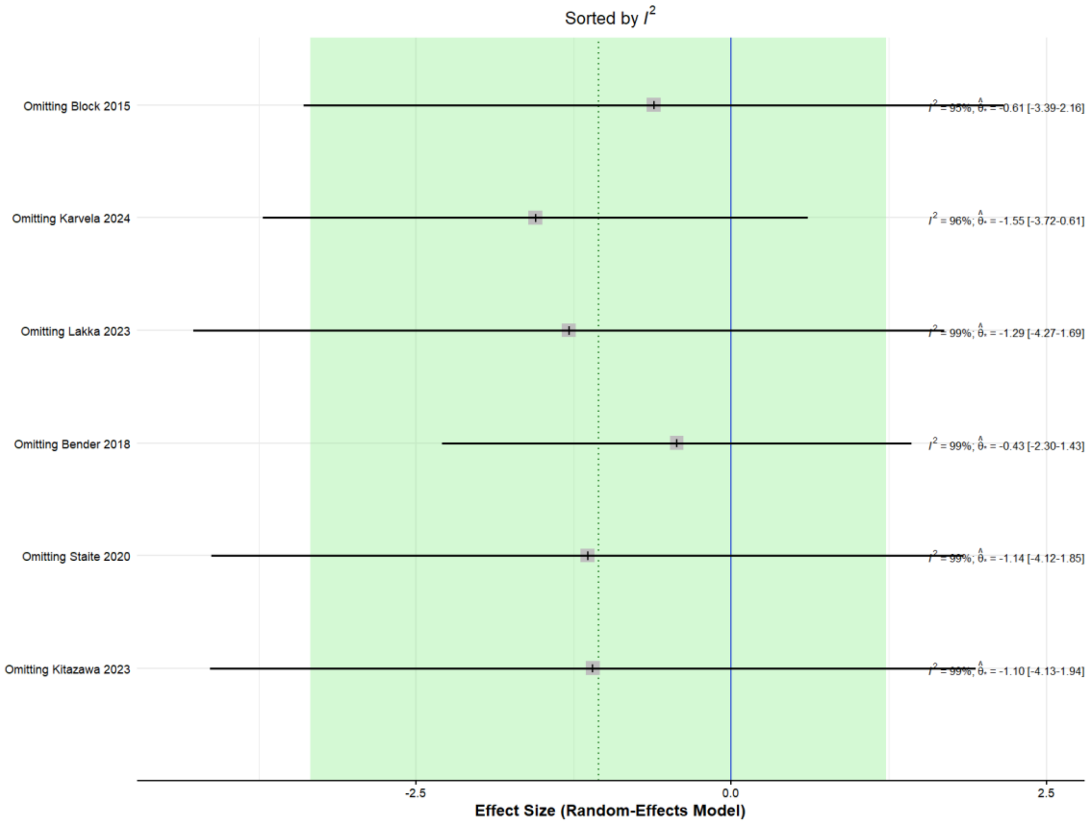

LDL

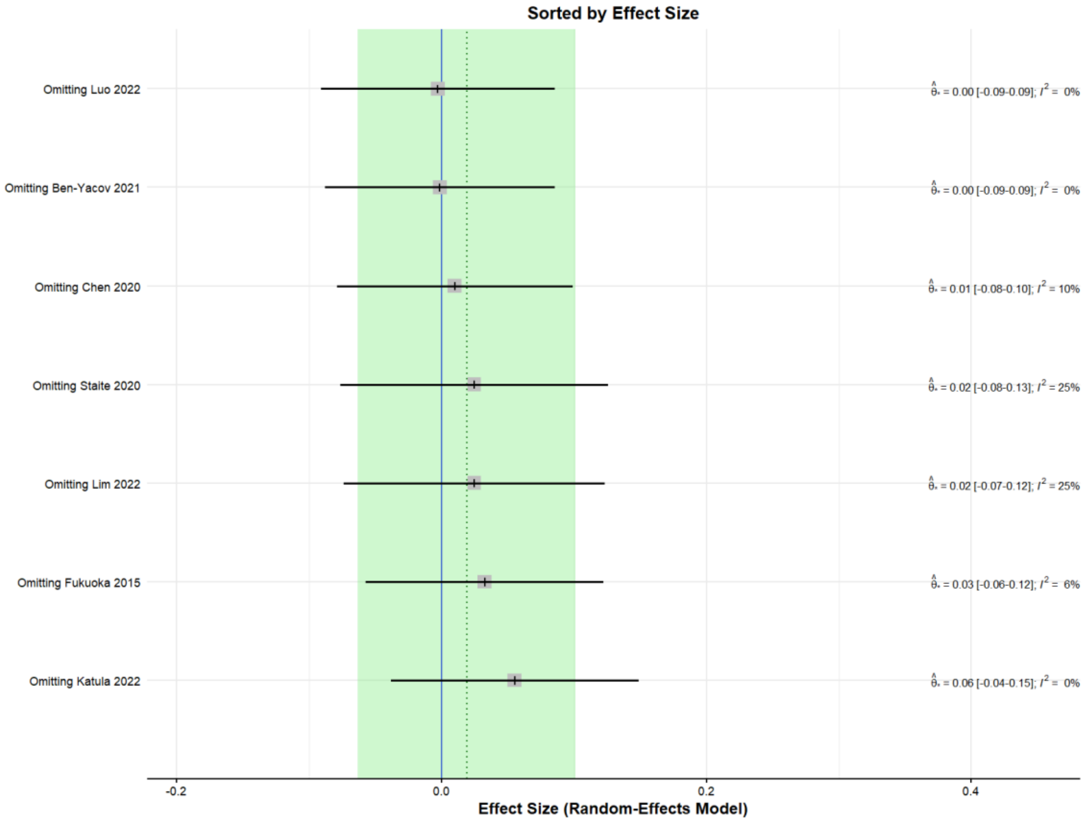
Sorted by Effect Size
Omitting Luo 2022
Omitting Ben-Yacov 2021
Omitting Chen 2020
Omitting Staite 2020
Omitting Lim 2022
Omitting Fukuoka 2015
Omitting Katula 2022
$\hat{\theta}_*$ = 0.00 [-0.09-0.09]; I^2 = 0%
$\hat{\theta}_*$ = 0.00 [-0.09-0.09]; I^2 = 0%
$\hat{\theta}_*$ = 0.01 [-0.08-0.10]; I^2 = 10%
$\hat{\theta}_*$ = 0.02 [-0.08-0.13]; I^2 = 25%
$\hat{\theta}_*$ = 0.02 [-0.07-0.12]; I^2 = 25%
$\hat{\theta}_*$ = 0.03 [-0.06-0.12]; I^2 = 6%
$\hat{\theta}_*$ = 0.06 [-0.04-0.15]; I^2 = 0%
-0.2
0.0
0.2
0.4
Effect Size (Random-Effects Model)

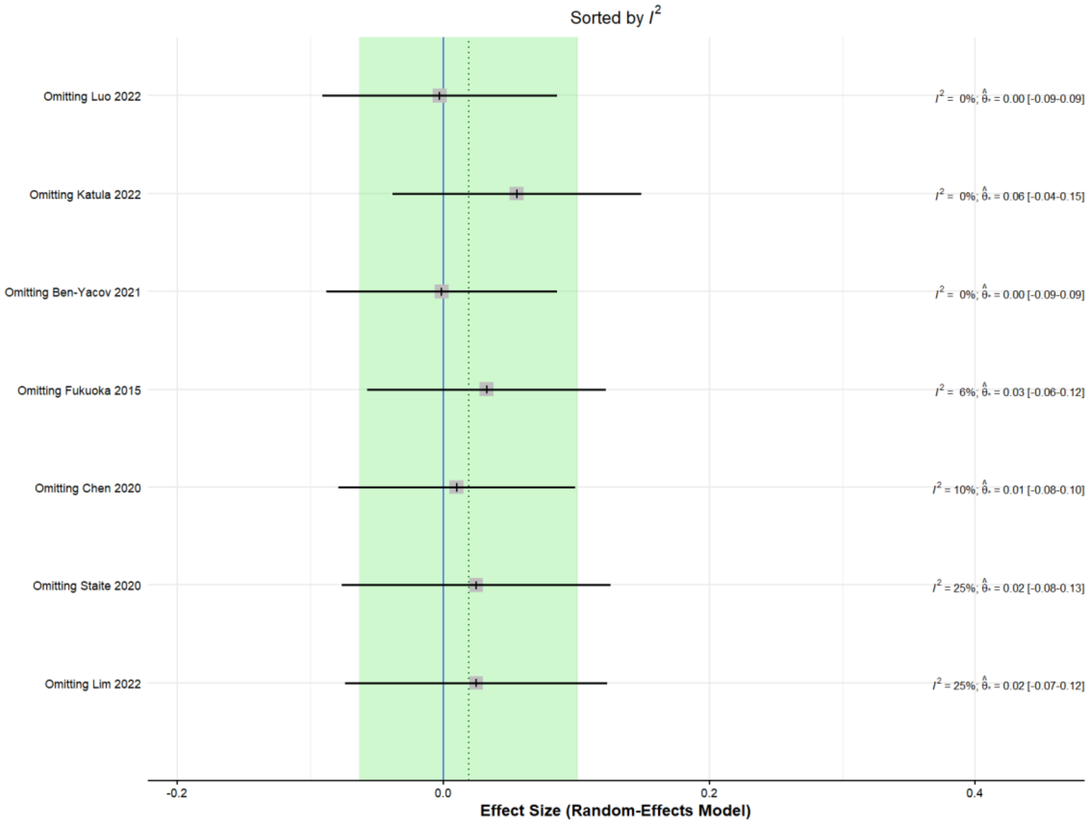
Sorted by I^2
Omitting Luo 2022
Omitting Katula 2022
Omitting Ben-Yacov 2021
Omitting Fukuoka 2015
Omitting Chen 2020
Omitting Staite 2020
Omitting Lim 2022
I^2 = 0%; $\hat{\theta}_*$ = 0.00 [-0.09-0.09]
I^2 = 0%; $\hat{\theta}_*$ = 0.06 [-0.04-0.15]
I^2 = 0%; $\hat{\theta}_*$ = 0.00 [-0.09-0.09]
I^2 = 6%; $\hat{\theta}_*$ = 0.03 [-0.06-0.12]
I^2 = 10%; $\hat{\theta}_*$ = 0.01 [-0.08-0.10]
I^2 = 25%; $\hat{\theta}_*$ = 0.02 [-0.08-0.13]
I^2 = 25%; $\hat{\theta}_*$ = 0.02 [-0.07-0.12]
-0.2
0.0
0.2
0.4
Effect Size (Random-Effects Model)

Weight (%)

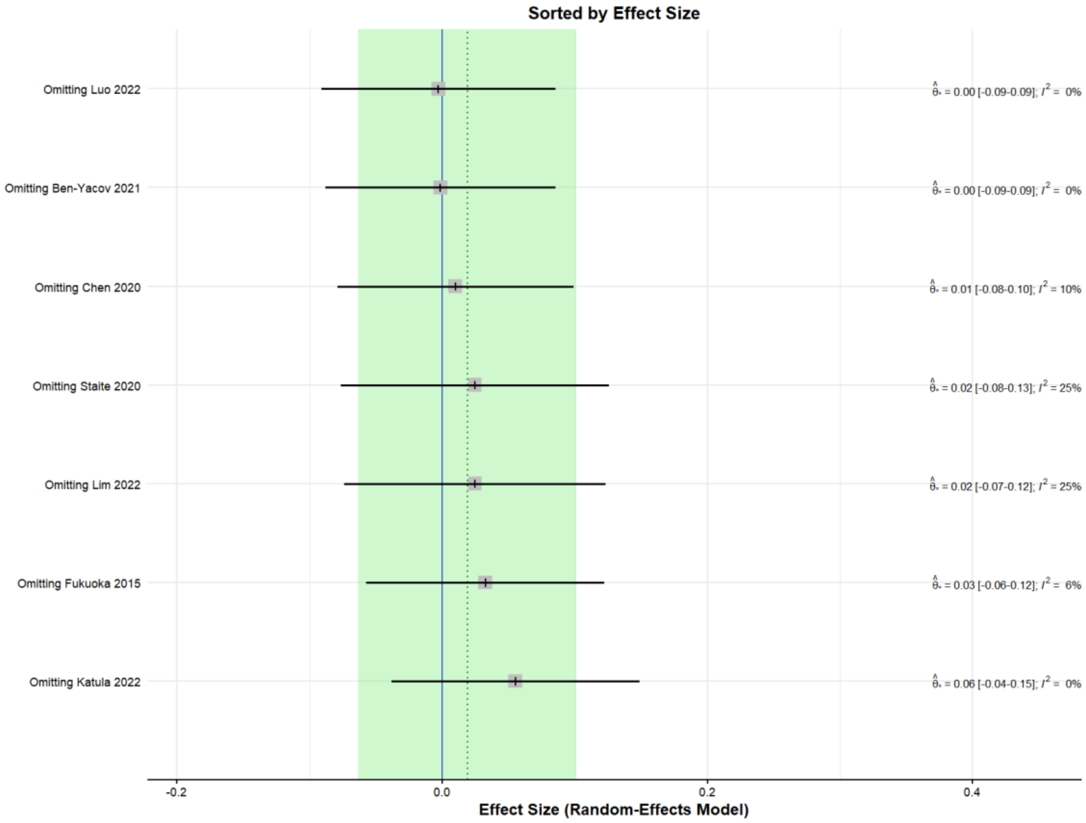

Sorted by Effect Size
Omitting Luo 2022
Omitting Ben-Yacov 2021
Omitting Chen 2020
Omitting Staite 2020
Omitting Lim 2022
Omitting Fukuoka 2015
Omitting Katula 2022
θ̂* = 0.00 [-0.09-0.09]; I² = 0%
θ̂* = 0.00 [-0.09-0.09]; I² = 0%
θ̂* = 0.01 [-0.08-0.10]; I² = 10%
θ̂* = 0.02 [-0.08-0.13]; I² = 25%
θ̂* = 0.02 [-0.07-0.12]; I² = 25%
θ̂* = 0.03 [-0.06-0.12]; I² = 6%
θ̂* = 0.06 [-0.04-0.15]; I² = 0%
-0.2
0.0
0.2
0.4
Effect Size (Random-Effects Model)

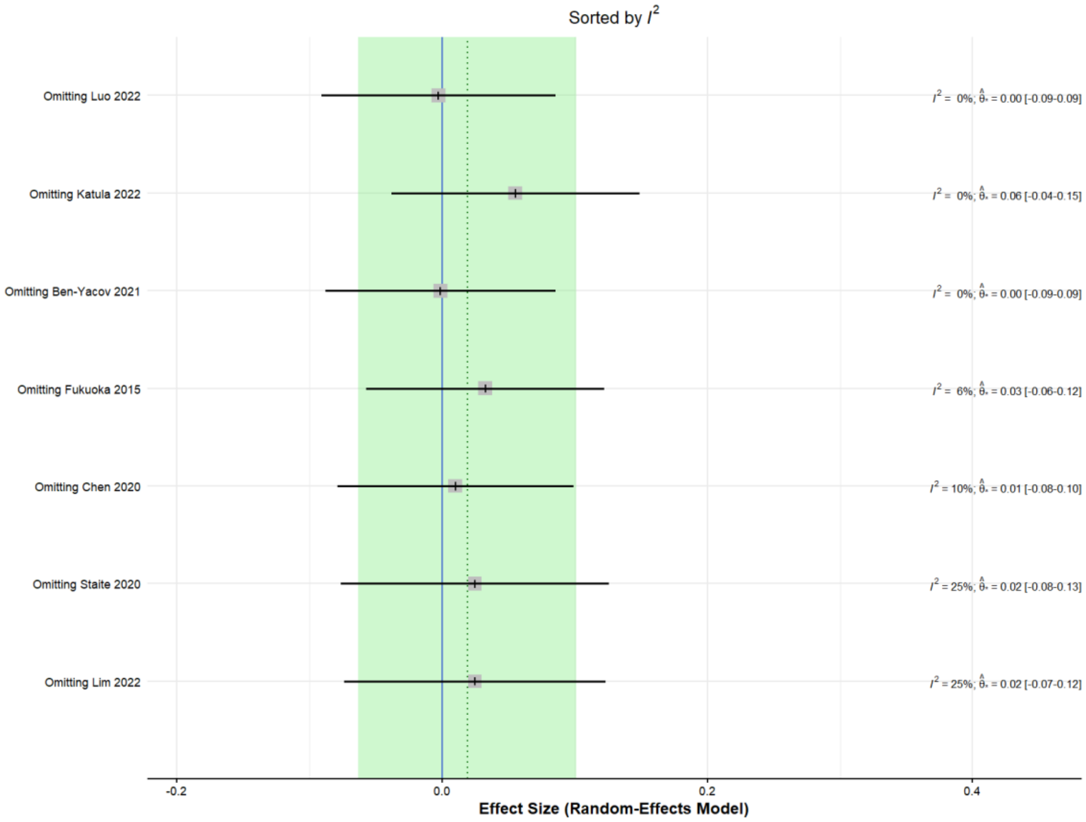

Sorted by I²
Omitting Luo 2022
Omitting Katula 2022
Omitting Ben-Yacov 2021
Omitting Fukuoka 2015
Omitting Chen 2020
Omitting Staite 2020
Omitting Lim 2022
I² = 0%; θ̂* = 0.00 [-0.09-0.09]
I² = 0%; θ̂* = 0.06 [-0.04-0.15]
I² = 0%; θ̂* = 0.00 [-0.09-0.09]
I² = 6%; θ̂* = 0.03 [-0.06-0.12]
I² = 10%; θ̂* = 0.01 [-0.08-0.10]
I² = 25%; θ̂* = 0.02 [-0.08-0.13]
I² = 25%; θ̂* = 0.02 [-0.07-0.12]
-0.2
0.0
0.2
0.4
Effect Size (Random-Effects Model)

Appendix S29: Sensitivity analysis Fukuoka et al.

HbA1c

Correlation coefficient	MD (95% CI)	Tau (95% CI)	I^2
R=0.95	-0.0747 [-0.0976; -0.0517]	0.0117 [0.0000; 0.0686]	12.5%
R=0.9	-0.0755 [-0.0988; -0.0522]	0.0114 [0.0000; 0.0693]	11.1%
R=0.7	-0.0762 [-0.0998; -0.0527]	0.0111 [0.0000; 0.0700]	10.0%
R=0.5	-0.0764 [-0.1000; -0.0528]	0.0110 [0.0000; 0.0700]	9.7%

SBP

Correlation coefficient	MD (95% CI)	Tau (95% CI)	I^2
R=0.95	-1.4905 [-3.3531; 0.3721]	1.5903 [0.4566; 4.2080]	67.5%
R=0.9	-1.3516 [-3.0887; 0.3856]	1.3329 [0.0000; 3.9325]	57.7%
R=0.5	-1.0467 [-2.3557; 0.2623]	1.3329 [0.0000; 3.9325]	57.7%
R=0.7	-1.1062 [-2.5340; 0.3216]	0.9970 [0.0000; 3.4951]	41.6%

DBP

Correlation coefficient	MD (95% CI)	Tau (95% CI)	I^2
R=0.95	-1.3195 [-2.6732; 0.0341]	1.1971 [0.3965; 3.1368]	67.9%
R=0.9	-1.1894 [-2.4552; 0.0764]	0.9863 [0.0000; 2.9525]	58.8%
R=0.7	-0.8877 [-1.8837; 0.1083]	0.6240 [0.0000; 2.6080]	34.1%
R=0.5	-0.7620 [-1.6165; 0.0926]	0.4674 [0.0000; 2.4405]	22.2%

FPG

Correlation coefficient	MD (95% CI)	Tau (95% CI)	I^2
R=0.95	-0.1117 [-0.2368; 0.0133]	0.1246 [0.0779; 0.3109]	93.9%
R=0.9	-0.1126 [-0.2383; 0.0131]	0.1250 [0.0776; 0.3142]	93.8%
R=0.7	-0.1151 [-0.2424; 0.0123]	0.1254 [0.0767; 0.3200]	93.7%
R=0.5	-0.1165 [-0.2449; 0.0118]	0.1254 [0.0760; 0.3225]	93.7%

HDL

Correlation coefficient	MD (95% CI)	Tau (95% CI)	I^2
R=0.95	0.0245 [-0.0193; 0.0683]	0.0298 [0.0162; 0.0976]	69.1%
R=0.9	0.0289 [-0.0087; 0.0665]	0.0247 [0.0087; 0.0902]	59.7%
R=0.7	0.0331 [0.0041; 0.0620]	0.0193 [0.0000; 0.0787]	46.8%
R=0.5	0.0337 [0.0072; 0.0602]	0.0178 [0.0000; 0.0739]	42.7%

LDL

Correlation coefficient	MD (95% CI)	Tau (95% CI)	I^2
R=0.95	0.0145 [-0.0695; 0.0985]	0.0414 [0.0000; 0.1825]	19.9%
R=0.9	0.0191 [-0.0631; 0.1014]	0.0287 [0.0000; 0.1769]	9.6%
R=0.7	0.0232 [-0.0564; 0.1029]	0 [0.0000; 0.1674]	0.0%
R=0.5	0.0252 [-0.0538; 0.1043]	0 [0.0000; 0.1623]	0.0%

Total Cholesterol

Correlation coefficient	MD (95% CI)	Tau (95% CI)	I^2
R=0.95	-0.0383 [-0.1560; 0.0795]	0.0840 [0.0000; 0.5849]	59.9%
R=0.9	-0.0296 [-0.1383; 0.0792]	0.0730 [0.0000; 0.5906]	50.6%
R=0.7	-0.0175 [-0.1111; 0.0761]	0.0614 [0.0000; 0.6022]	40.0%
R=0.5	-0.0146 [-0.1034; 0.0742]	0.0583 [0.0000; 0.6078]	37.1%

Triglycerides

Correlation coefficient	MD (95% CI)	Tau (95% CI)	I^2
R=0.95	-0.0889 [-0.2559; 0.0780]	0.1434 [0.0722; 0.3964]	80.8%
R=0.9	-0.0857 [-0.2515; 0.0801]	0.1361 [0.0675; 0.3939]	76.3%
R=0.7	-0.0769 [-0.2421; 0.0883]	0.1284 [0.0582; 0.3932]	71.3%
R=0.5	-0.0724 [-0.2376; 0.0928]	0.1263 [0.0515; 0.3934]	69.9%

Appendix S30: Egger's test results and funnel plots

Outcome	N RCTs	Intercept	Confidence Interval	t	p
Weight (kg)	13	3.90	[-0.58; 8.38]	1.70	0.12
BMI (kg/m^2)	11	3.33	[-1.70; 8.37]	1.30	0.23
HbA1c (%)	11	0.32	[-0.80; 1.45]	0.56	0.59

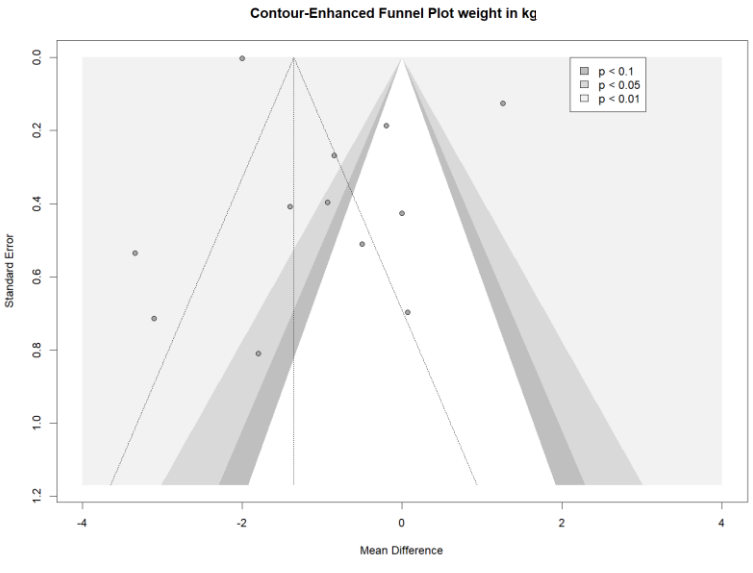

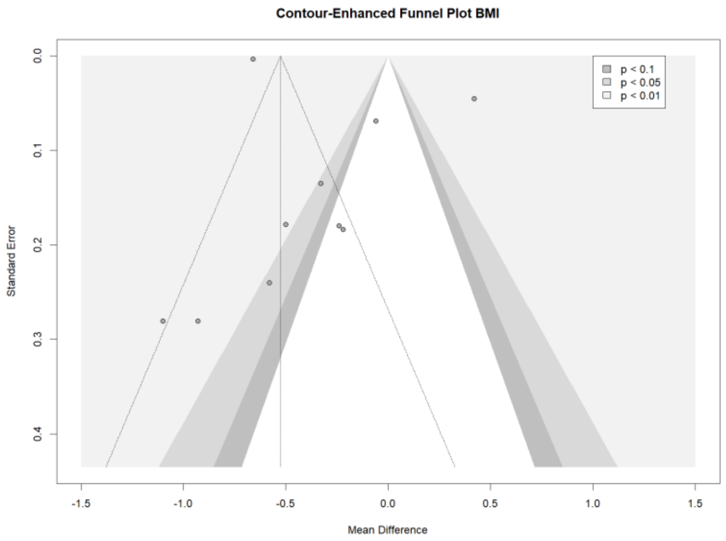

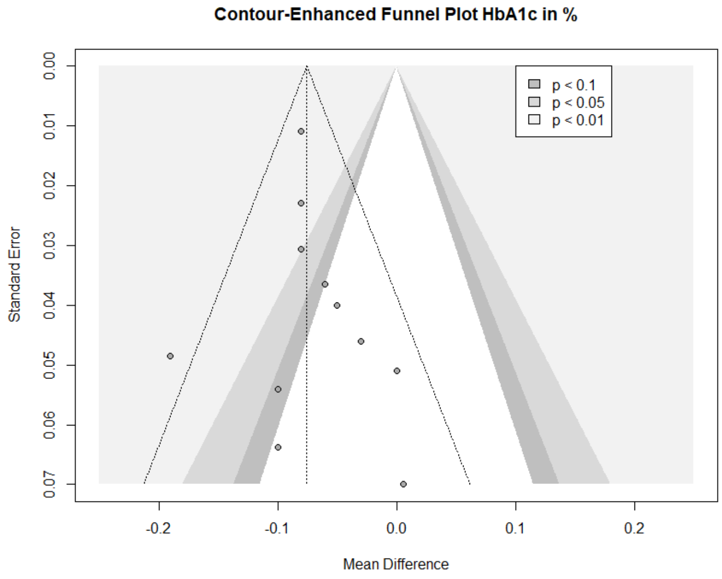

References

1. Glasgow RE, Harden SM, Gaglio B, Rabin B, Smith ML, Porter GC, et al. RE-AIM planning and evaluation framework: adapting to new science and practice with a 20-year review. Frontiers in public health. 2019;7:64.
2. Pfadenhauer LM, Gerhardus A, Mozygemba K, Lysdahl KB, Booth A, Hofmann B, et al. Making sense of complexity in context and implementation: the Context and Implementation of Complex Interventions (CICI) framework. Implementation science. 2017;12:1-17.
3. Goryakin Y, Suhlrie L, Cecchini M. Impact of primary care-initiated interventions promoting physical activity on body mass index: systematic review and meta-analysis. Obesity Reviews. 2018;19(4):518-28.
4. Higgins JP, Li T, Deeks JJ. Choosing effect measures and computing estimates of effect. Cochrane handbook for systematic reviews of interventions. 2019:143-76.
